# Transylvanian Grape Pomaces as Sustainable Sources of Antioxidant Phenolics and Fatty Acids—A Study of White and Red Cultivars

**DOI:** 10.3390/antiox14101152

**Published:** 2025-09-23

**Authors:** Veronica Sanda Chedea, Liliana Lucia Tomoiagă, Mariana Ropota, Gabriel Marc, Floricuta Ranga, Maria Doinița Muntean, Alexandra Doina Sîrbu, Ioana Sorina Giurca, Maria Comșa, Ioana Corina Bocsan, Anca Dana Buzoianu, Hesham Kisher, Raluca Maria Pop

**Affiliations:** 1 Research Station for Viticulture and Enology Blaj (SCDVV Blaj), Gheorhe Barițiu Street, No 2, 515400 Blaj, Romania; tomoiagaliliana@yahoo.com (L.L.T.); maria.doinita@gmail.com (M.D.M.); sirbu.alexandra@ymail.com (A.D.S.); tirnovean.ioana@gmail.com (I.S.G.); comsamaria1970@gmail.com (M.C.); 2 Laboratory of Chemistry and Nutrition Physiology, National Research Development Institute for Animal Biology and Nutrition (IBNA Balotesti), Balotesti, 077015 Ilfov, Romania; m.ropota@yahoo.com; 3 Department of Organic Chemistry, “Iuliu Hațieganu” University of Medicine and Pharmacy, 41 Victor Babeș Street, 400012 Cluj-Napoca, Romania; marc.gabriel@umfcluj.ro; 4 Food Science and Technology, Department of Food Science, University of Agricultural Science and Veterinary Medicine Cluj-Napoca, Calea Mănăștur, No 3-5, 400372 Cluj-Napoca, Romania; flori-cutza_ro@yahoo.com; 5 Pharmacology, Toxicology and Clinical Pharmacology, Department of Morphofunctional Sciences, “Iuliu Haţieganu” University of Medicine and Pharmacy, Victor Babeș, No 8, 400012 Cluj-Napoca, Romania; bocsan.corina@umfcluj.ro (I.C.B.); abuzoianu@umfcluj.ro (A.D.B.); 6 School of Applied Sciences, University of the West of England, Bristol BS16 1QY, UK; hesham.kisher@uwe.ac.uk

**Keywords:** white grape pomace, red grape pomace, phenolics, fatty acids, antioxidant activity

## Abstract

Grape pomace (GP), a significant by-product of winemaking, is gaining increasing recognition for its potential as a source of bioactive compounds with antioxidant and cardioprotective properties. This study aimed to characterize the polyphenolic profile, fatty acid composition, and antioxidant activity of 17 GP samples from Transylvanian cultivars. Polyphenolic content was determined using the Folin–Ciocalteu method and high-performance liquid chromatography coupled with diode array detection and electrospray ionization mass spectrometry (HPLC–DAD–ESI MS) analysis. Fatty acid composition was analyzed using gas chromatography with flame ionization detection (GC–FID). Antioxidant capacity was assessed using five methods, which included the 2,2-diphenyl-1-picrylhydrazyl (DPPH) radical scavenging, 2,2′-azino-bis (3-ethylbenzothialzoline-6-sulfonic acid) (ABTS) radical scavenging, ferric-reducing antioxidant power (FRAP), cupric ion reducing antioxidant capacity (CUPRAC), and reducing power (RP) assays. Additionally, all extracts were analyzed by Fourier transform infrared (FTIR) spectroscopy to identify the presence of functional groups and chemical bonds associated with bioactive compounds. The results showed that Neuburger (NE), Radames (RA), and Regent (RE) cultivars had the highest phenolic concentrations, particularly of catechin, epicatechin, and procyanidin dimers. NE and Feteascǎ Regalǎ (FR) exhibited the greatest radical scavenging and electron transfer activities across multiple antioxidant assays. Rose Blaj (RB) and Astra (AS) displayed the most favorable fatty acid profiles, with high unsaturated-to-saturated fatty acid (UFA/SFA) and hypocholesterolemic-to-hypercholesterolemic fatty acid (H/H) ratios, as well as low atherogenicity (AI) and thrombogenicity (TI) indices, suggesting cardioprotective potential. Additionally, RB and NE cultivars also demonstrated a strong chelation of Cu^2+^ and Fe^2+^ ions, enhancing their antioxidant efficacy by mitigating metal-catalyzed oxidative stress. These findings underscore the potential of GP, particularly from NE, RB, RA, and AS cultivars, the last three of which were homologated in Transylvania at SCDVV Blaj, as valuable sources of health-promoting compounds for use in food, nutraceuticals, and other health-related applications.

## 1. Introduction

FAOSTAT reports that global grape output reached 72.5 million tons in 2023, with Romania producing 1.04 million tons [1]. According to the OIV’s State of the World Vine and Wine Sector in 2024, the global vineyard surface area totaled 7.1 million hectares (−0.6% vs. 2023), world wine production reached 226 million hectoliters (−4.8%), and wine consumption was 214 million hectoliters (−3.3%). International trade remained broadly stable at 99.8 million hectoliters exported (−0.1%), with an average export price of 3.6 euros/L (−0.3%) and a total export value of 35.9 billion euros (−0.3%) [2]. Together, these indicators portray a sector that continued to contract in 2024, with modest price softening but relatively steady trade flows.

During the winemaking process, grape pomace (GP) is generated as a by-product and accounts for approximately 10% to 30% of the total mass of the crushed grapes [3,4]. Specifically, in Transylvania, the GP production represents 18–26% of the grapes entering vinification [5]. The yield of GP can vary significantly depending on factors such as moisture content, freshness [6], grape cultivar, and terroir [7,8].

In recent years, the pursuit of sustainable solutions within the agri-food sector has intensified due to the need to reduce industrial waste and increasing environmental concerns. Recent research [9,10,11] has demonstrated that GP valorization may provide significant health benefits, as well as reduce waste production. The valorization of GP has been explored across various contexts, including animal nutrition [11,12,13,14], the food industry [9,11], cosmetics [15], and pharmacology [11,16,17,18]. These applications are largely driven by the presence of bioactive compounds in GP, which are influenced by grapevine cultivar, terroir, and viticultural and winemaking practices [7,19]. GP is rich in nutritional and bioactive constituents, including carbohydrates (~12–40%), fibers (~17–88%), proteins (~4–15%), lipids (~2–14%), vitamins and minerals (~2–7%), and polyphenols (~0.2–9%) [3].

Both white and red GP are known for their high polyphenol content, including cinnamic acids (e.g., p-coumaric acid), benzoic acids (e.g., syringic, gallic, protocatechuic, and 4-hydrohxybenzoic acids), flavan-3-ols (e.g., catechin, epicatechin), proanthocyanidins, and flavonols (e.g., myricetin, quercetin, kaempferol) [6,9,18]. These compounds have been reported to exhibit a range of pharmacological properties, such as anti-inflammatory, antifungal, antibacterial, and antioxidant activities [17,20,21,22]. The antioxidant activity is among the most extensively studied, as phenolic compounds can act as hydrogen donors, metal chelators, free radical scavengers, and singlet oxygen quenchers [23,24], contributing to their potential health-protective properties.

A core constituent of GP is the seed fraction, which is rich in fatty acids, primarily unsaturated fatty acids [25]. The predominant fatty acids in GP include oleic acid (C18:1), cis-linoleic acid (C18:2), and linolenic acid (C18:3). However, it is important to note that fatty acid composition varies between cultivars [25]. Cis-linoleic and linolenic acids are essential fatty acids that cannot be synthesized in the human body but are essential for healthy human metabolism, rendering their dietary consumption essential [26,27].

Located between 46° to 47° N latitude and 23° to 24° E longitude, the Târnave vineyards form part of the viticultural zone 1 in Romania and are situated on the Transylvanian Plateau [28,29,30,31]. This region encompasses the most prominent viticultural area in Transylvania, known as the prestigious Târnave vineyard. The importance of the Târnave vineyard is evident in its cultivated area, the diversity of grapevine cultivars, and the quality of wines produced, which include dry, semi-dry, semi-sweet, semi-aromatic, aromatic, and sparkling wines with Protected Designation of Origin and Protected Geographical Indication status. These wines are derived from established cultivars such as Feteascǎ Albǎ, Feteascǎ Regalǎ, Italian Riesling, Sauvignon Blanc, Muscat Ottonel, and Neuburger [28,29,30,32]. As, during the production of these wines, an important amount of GP is generated, this research is a unique contribution to the circular economy of the Transylvanian winemaking industry.

This work is the first study aiming to provide insights into the composition of 17 GP samples from Transylvanian cultivars, focusing on their polyphenol and fatty acid content, as well as their antioxidant activity. Through evaluating both white and red grape varieties, this research aims to help determine whether the sustainable valorization of GP can provide a source of bioactive compounds with potential benefits to human health.

## 2. Materials and Methods

### 2.1. Chemicals

The following reagents and solvents were used in this investigation: acetonitrile (HPLC grade), acetic acid, ethanol, methanol (MS grade), hexane, and petroleum ether. Other chemicals that were utilized included copper(II) chloride (CuCl_2_), neocuproine (2,9-dimethyl-2,10-phenanthroline, C_14_H_12_N_2_), potassium hexacyanoferrate(III) (K_3_[Fe(CN)_6_])), trichloroacetic acid (Cl_3_CCOOH), iron(III) chloride (FeCl_3_), and ferrozine (3-(2-pyridyl)-5,6-diphenyl-1,2,4-triazzine-p,p’-disulfonic acid monosodium salt hydrate (C_8_H_8_N_6_O_6_·xH_2_O). Murexide (5,5′-nitrilodibarbituric acid monoammonium salt, C_8_H_8_N_6_O_6_, and the Supelco 37 component FAME Mix were sourced from Merck (Darmstadt, Germany). Additional reagents, including Folin–Ciocalteu reagent, diphenyl-1-picrylhydrazyl (DPPH), Trolox, ABTS (2,2′-azinobis-(3-ethylbenzthiazolin-6-sulfonic acid)), FRAP reagent, quercetin, catechin, and gallic acid (all ≥99% HPLC grade), were obtained from Sigma Co. (St. Louis, MO, USA). Sodium carbonate (Na_2_CO_3_), anhydrous sodium sulfate (Na_2_SO_4_), and sulfuric acid (H_2_SO_4_) were purchased from Amex (Bucharest, Romania).

### 2.2. GP Generation and Conditioning

GP, comprising the stems, skins, and seeds of white and red wine grapes (*Vitis vinifera* L.), was sourced from the SCDVV Blaj winery, located in the Târnave Wine Center, Romania. All grapevine cultivars, both white and red, were grown in the Crăciunelu de Jos vineyard (Târnave vineyard, Alba County, Romania), and the grapes were harvested between 12 and 18 September 2019. The white grape cultivars included Blasius (BL), Rhine Riesling (RR), Roze Blaj (RB), Astra (AS), Traminer roz (TR), Johaniter (JO), Neuburger (NE), Rubin (RU), Sauvignon Blanc (SB), Fetească Regală (FR), Radames (RA), Brumăriu (BR), Selena (SE), and Muscat Ottonel (MO) (Table 1). The red grape cultivars included Regent (RE), Syrah (SH), and Amurg (AM) (Table 1). Among these, Blasius (BL), Roze Blaj (RB), Astra (AS), Rubin (RU), Radames (RA), Brumăriu (BR), Selena (SE), and Amurg (AM) were created and homologated at SCDVV Blaj (Table 1). All the grapevine cultivars have been approved [33] and were taken in this study due to the need to valorize the GP generated from all of them during the winemaking process.

Following grape pressing, the resulting pomace was collected and air-dried in a well-ventilated room at ambient temperature (23 °C, 50% humidity). The dried samples were subsequently stored in paper bags under controlled conditions (reduced light exposure and refrigeration at 4 °C) to minimize oxidative degradation for a period of three months prior to extraction.

### 2.3. GP Polyphenols Extraction

The raw GP material was ground in a Cyclone Mill-MC5 (Tecator, Höganäs, Sweden) until the particle size was 1 mm. A quantity of 0.1 g of GP powder was extracted at room temperature (20 °C) in a 3 mL solvent mixture of water:ethanol (30:70, *v*/*v*) using foil-wrapped vessels to prevent light exposure. The mixtures were stirred at 600 RPM for 2 h and filtered through Whatman filter paper. The obtained extracts were preserved at −80 °C until analysis. The obtained GP extracts were labeled according to the cultivar as follows: BL, RR, RB, AS, TR, JO, NE, RU, SB, FR, RA, BR, SE, MO, RE, SH, AM.

### 2.4. Total Polyphenol Content (TPC) of GP Polyphenol Extracts

The TPC of the GP extracts was determined using the Folin–Ciocalteu method, as described by Pop et al. [35]. Accordingly, 25 μL of each GP extract was mixed with 125 μL of 0.2 N Folin–Ciocalteu reagent and 100 μL of sodium carbonate solution (7.5% *w*/*v*). The mixture was homogenized and incubated in the dark at 25 °C for 2 h. Absorbance at 760 nm was recorded using a Synergy HT Multi-Detection Microplate Reader (BioTek Instruments, Inc., Winooski, VT, USA). Gallic acid was used to construct the calibration curve (R^2^ = 0.9945) (Figure S1), and results were expressed as milligrams of gallic acid equivalents per g dry weight (dw) GP (mg GAE/g GP). All measurements were performed in triplicate (*n* = 3), and the data presented as means and standard deviations.

### 2.5. Fourier Transform Infrared Spectroscopy (FTIR) Analysis

All 17 GP extracts were analyzed using Fourier transform infrared (FTIR) spectroscopy with a Shimadzu IR Prestige-21 spectrophotometer (Shimadzu Handelsgesellschaft mbH, Bucharest, Romania), equipped with a horizontal attenuated total reflectance diamond accessory featuring single reflection (PIKE Technologies, Fitchburg, WI, USA). The water:ethanol (30:70, *v*/*v*) solvent mixture was used as a background reference. Spectra were recorded in the range of 4000–600 cm^−1^ at a resolution of 4 cm^−1^, with 64 scans performed. Post-acquisition baseline correction was applied to minimize residual contributions from solvent and atmospheric H_2_O/CO_2_, ensuring the clearer identification of extract-specific functional groups. Characteristic absorption bands corresponding to various chemical bonds and functional groups were identified. The primary spectral data were further processed and analyzed using the IR solution software overview, version 1.30 (Shimadzu, Northampton, Handelsgesellschaft mbH, Bucharest, Romania) and Origin^®^ 7SR1 Software (OriginLab Corporation, Northampton, MA, USA).

### 2.6. Liquid Chromatography–Diode Array Detection–Electro-Spray Ionization Mass Spectrometry (HPLC–DAD–ESI MS) Analysis

The qualitative and quantitative analysis of phenolic compounds in GP extracts was carried out using an Agilent 1200 HPLC with a diode array detector (DAD) and coupled with an Agilent 6110 single-quadrupole mass spectrometer (MS), following the methodology described by Pop et al. [36] and Chedea et al. [37]. Chromatographic separation was performed on an Eclipse XDB-C18 column (4.6 × 150 mm, 5 μm particle size; Agilent Technologies, Santa Clara, CA, USA) and two mobile phases, solvent A (0.1% acetic acid/acetonitrile (99:1) in distilled water (*v*/*v*)) and solvent B (0.1% acetic acid in acetonitrile (*v*/*v*)) were run for 30 min at a column temperature of 25 °C at a flow of 0.5 mL/min. The injection volume was 20 μL GP extract. The used gradient (expressed as % B) was 0 min, 5% B; 0–2 min, 5% B; 2–18 min, 5–40% B; 18–20 min, 40–90% B; 20–24 min, 90% B; 24–25 min, 90–5% B; 25–30 min, 5% B. The chromatograms were registered at λ values equal to 280, 340, and 520 nm, while full UV–Vis spectra were recorded in the 200–600 nm wavelength range. All analyses were conducted in triplicate (*n* = 3).

Mass spectrometric detection was performed using an electrospray ionization in positive mode (ESI+) with a capillary voltage of 3000 V, a source temperature of 300 °C, and a nitrogen gas flow rate of 7 L/min. Full-scan MS data were acquired in the *m/z* range of 100–1200. Chromatograms, spectra acquisition, and analysis were acquired and processed using Agilent ChemStation software (Rev B.04.02 SP1, Palo Alto, CA, USA). Phenolic compounds were identified based on their retention times, UV–Vis spectra, and mass spectral data. Quantification was achieved using calibration curves (Figure S1 and Table S1) prepared from standard solutions of catechin (R^2^ = 0.9985; LOD = 0.18 μg/mL, LOQ = 0.72 μg/mL), gallic acid (R^2^ = 0.9978; LOD = 0.36 μg/mL, LOQ = 1.44 μg/mL), rutin (R^2^ = 0.9981; LOD = 0.21 μg/mL, LOQ = 0.84 μg/mL), and cyanidin (R^2^ = 0.9951; LOD = 0.36 μg/mL, LOQ = 1.44 μg/mL). Hydroxybenzoic acids were quantified as gallic acid equivalents, flavanols as catechin equivalents, flavonols as rutin equivalents, and anthocyanins as cyanidin equivalents.

### 2.7. Gas Chromatography with Flame Ionization Detector (GC–FID Analysis)

The total lipid contents were extracted from dried GP using petroleum ether in a Foss Soxtec 2055 extraction system (Effretikon, Switzerland). The extracted lipids were then subjected to transesterification in methanol containing 3% concentrated sulfuric acid at 80 °C for 4 h to convert the fatty acids into their corresponding methyl esters (FAMEs). FAMEs were analyzed by injecting 1 μL of a sample into a Perkin Elmer Clarus 500 gas chromatograph (Bucharest, Romania) equipped with a BPX70 capillary column (60 m × 0.25 mm i.d., 0.25 μm film thickness) and a flame ionization detector (FID). Hydrogen was used as a carrier gas at a flow rate of 35 cm/s at 180 °C, while air served as the combustion gas at a flow rate of 420 mL/min. The split ratio was set at 1:100. The column temperature was adjusted by 5 °C from 180 °C to 220 °C, and the injector and detector temperatures were 250 °C and 260 °C, respectively. FAMEs were separated based on chain length, degree of unsaturation, and double-bond geometry. A control sample, n-hexane, and a certified reference material (Supelco 37 Component FAME Mix) were analyzed in parallel with each sample batch, as described by Habeanu et al. [38]. Identification of FAMEs was performed by comparing retention times with those of known standards. Results were expressed as grams of fatty acid per 100 g of total fatty acids. All measurements were conducted in duplicate (*n* = 2).

The calculated oxidizability (COX) values, atherogenicity index (AI), thrombogenicity index (TI), and ratio of hypocholesterolemeic-to-hypercholesterolemic fatty acids (H/H) were determined according to the methodology described in a previous study [24].

### 2.8. Antioxidant Properties

#### 2.8.1. Antiradical Assays

• Measurement of Relative DPPH Radical Scavenging Capacity.

The radical scavenging activity of each GP extract (BL, RR, RB, AS, TR, JO, NE, RU, SB, FR, RA, BR, SE, MO, RE, SH, AM) was evaluated using the 2,2-diphenyl-1-picrylhydrazyl assay following the methodology described by Pop et al. [36]. A 250 μL volume of each extract was mixed with 170 μL of a 0.02 mg/mL DPPH solution prepared in methanol. The mixtures were incubated at room temperature for 30 min in the dark. Absorbance was measured at 517 nm using a Synergy HT Multi-Detection Microplate Reader (BioTek Instruments, Inc., Winooski, VT, USA). Trolox was used to generate a calibration curve (R^2^ = 0.9942) (Figure S1), and the results are expressed as Trolox equivalents per milliliter of extract (TE/mL). The DPPH radical scavenging activity of the GP extracts was calculated and expressed as median with an interquartile range (25th–75th percentile) based on triplicate measurements (*n* = 3).

• Measurement of ABTS Cation Radical Scavenging Capacity (ABTS).

The ABTS˙^+^ (2,2′-Azinobis-(3-Ethylbenzthiazolin-6-Sulfonic Acid)) radical scavenging assay was performed according to the adapted protocol previously described by Re et al. [39] and Marc et al. [40]. A volume of 10 µL of 1:4 diluted GP extract was mixed with 90 µL of methanol and 3900 of ABTS˙^+^ solution, prepared as previously reported. The decrease in the absorbance of the resulting solution was measured spectrophotometrically (UV–Vis Jasco V-530 spectrophotometer, Jasco International Co., Tokyo, Japan) at 734 nm, using a blank composed of 100 µL methanol and 3900 µL ABTS˙^+^ reagent. A calibration curve was constructed using gallic standards, prepared by mixing various volumes of a gallic acid stock solution with methanol and processing them under the same conditions as the pomace extract samples. The calibration curve (Figure S1) was produced using gallic acid as the standard (R^2^ = 0.9956), and the results were calculated and expressed as milligrams of gallic acid equivalents per milliliter of extract (mg GAE/mL), reported as median with an interquartile range (25th–75th percentile) (*n* = 3).

#### 2.8.2. Electron Transfer Assays

• Measurement of Cupric Ion Reducing Antioxidant Capacity (CUPRAC).

For the CUPRAC (cupric ion-reducing antioxidant capacity) assay, 50 µL of GP extract, previously diluted 1:4 in methanol, was mixed with 1000 µL of 7.5 mM neocuproine, 1000 µL CuCl_2_ of 0.01 M CuCl_2_, and 1000 µL of 1 M ammonium acetate buffer. The mixture was thoroughly shaken and incubated in the dark for 30 min to allow the formation of a stable orange-colored complex, which exhibited a maximum absorbance at 450 nm (UV–Vis Jasco V-530 spectrophotometer, Jasco International Co., Tokyo, Japan). The calibration curve (Figure S1) was produced using gallic acid as the standard (R^2^ = 0.9969), and the results were calculated and expressed as milligrams of gallic acid equivalents per milliliter of extract (mg GAE/mL), reported as median with an interquartile range (25th–75th percentile) (*n* = 3).

• Measurement of Ferric-Reducing Antioxidant Potential (FRAP).

The ferric-reducing antioxidant potential (FRAP) assay was conducted using a modified version of the method originally developed by Benzie and Strain [41]. A 10 µL volume of GP extract, previously diluted 1:4 in methanol, was mixed with 2000 µL of 0.3 M acetate buffer (pH 3.6) and 1000 µL of freshly prepared FRAP reagent. The absorbance of the resulting solution was measured at λ = 593 nm (UV–Vis Jasco V-530 spectrophotometer, Jasco International Co., Tokyo, Japan) to assess antioxidant capacity. The calibration curve (Figure S1) was produced using gallic acid (R^2^ = 0.9936) as standard. The results were calculated and expressed as milligrams of gallic acid equivalents per milliliter of extract (mg GAE/mL) and reported as median with an interquartile range (25th–75th percentile) (*n* = 3).

• Measurement of Reducing Power (RP).

The reducing power (RP) assay was performed by mixing 10 µL of GP extract, previously diluted 1:4 in methanol, with 400 of 0.2 M phosphate buffer (pH 6.6) and 400 of 1% (*w*/*v*) potassium ferricyanide [K_3_Fe(CN)_6_] solution in test tubes. The tubes were sealed and incubated in a water bath at 50 °C for 20 min. After cooling to room temperature, the reaction was stopped by the addition of trichloroacetic acid. Finally, ferric chloride (FeCl_3_) was added to form Perl’s Prussian blue complex, which exhibits a maximum absorbance at 593 nm (UV–Vis Jasco V-530 spectrophotometer, Jasco International Co., Tokyo, Japan). The same assay protocol was applied to the reference compound gallic acid. The absorbance of all resulting mixtures was measured spectrophotometrically against a blank, and the results are presented as the average of three independent determinations (*n* = 3). The calibration curve (Figure S1) was produced using gallic acid as the standard (R^2^ = 0.9977), and the results are expressed as milligrams of gallic acid equivalents per milliliter of extract (mg GAE/mL), reported as median with an interquartile range (25th–75th percentile) (*n* = 3).

#### 2.8.3. Transition Metal Ion (Ferrous Fe^2+^ and Cupric Cu^2+^) Chelation Assays

The chelation potential of GP extracts for ferrous and cupric ions was determined spectrophotometrically (UV–Vis Jasco V-530 spectrophotometer, Jasco International Co., Tokyo, Japan) based on adapted protocols previously reported in several publications [40,42,43]. The presence of metal chelators in the sample leads to a reduction in absorbance due to the disruption of the chromogenic metal–ligand complex.

For ferrous ion chelation, the assay was based on the method described by Dinis et al. [44]. In this assay, 200 µL of GP extract was mixed with 500 µL of 0.125 mM FeSO_4_ and 500 µL of 0.315 mM ferrozine. After a 10 min incubation at room temperature, the absorbance was measured at λ = 562 nm against a blank. The intensity of the resulting red complex is directly proportional to the concentration of non-chelated ferrous ions in the solution.

The cupric ion chelation activity was assessed similarly, using a method adapted from Wu et al. [45]. In this assay, 100 µL of GP extract was combined with 400 µL of 3 mM CuSO_4_ prepared in a hexamine buffer (10 mM hexamine and 10 mM KCl). After 5 min, 75 µL of 1 mM murexide and 2 mL of distilled water were added. The mixture was incubated for an additional 5 min at room temperature. Absorbance was then recorded at 485 nm and 520 nm using a UV–Vis spectrophotometer (Jasco V-530 spectrophotometer, Jasco International Co., Tokyo, Japan). The two wavelengths correspond to the absorbance of the murexide–copper(II) complex and free murexide, respectively. The ratio of these absorbances is directly proportional to the concentration of unchelated copper(II) ions in the solution. Since free murexide exhibits a characteristic absorbance peak at 520 nm, this was considered in the analysis to ensure more accurate quantification, as described by Cesari et al. [46]. The formation of the purple murexide–copper complex indicates the presence of unchelated cupric ions, with a decrease in absorbance reflecting the chelating activity of the extract. The copper chelation capacity of the compounds was calculated using the following equation:

(1) $$cooper\;chelation\;\left(\%\right)=\frac{{\left(\frac{A_{485}}{A_{520}}\right)}_{control}-{\left(\frac{A_{485}}{A_{520}}\right)}_{sample}}{{\left(\frac{A_{485}}{A_{520}}\right)}_{control}}\times 100$$

The calibration curve for Fe^2+^ (Figure S1) and Cu^2+^ chelation was produced using EDTA as the standard (R^2^ = 0.9902), and results were expressed as micromolar EDTA equivalents per milliliter of extract (µM EE/mL), reported as median with an interquartile range (25th–75th percentile) (*n* = 3).

### 2.9. Statistical Analysis

Firstly, data analysis was performed using the software IBM SPSS Statistics, version 20 (SPSS Inc., Chicago, IL, USA). The normality of the data was assessed using normality tests and Q–Q plots. For variables that followed a normal distribution (total polyphenolic content), differences between groups were evaluated using one-way ANOVA, followed by Tukey’s post hoc test to identify specific group differences. For variables that did not meet the normality assumption (polyphenolic and fatty acid composition and antioxidant activity), the Kruskal–Wallis test, a non-parametric alternative, was applied. The significance values were adjusted using Bonferroni correction for multiple tests. Data were presented as mean ± standard deviation for normally distributed variables and as median with an interquartile range (25th–75th percentile) for non-normally distributed variables. A significance level of *p* < 0.05 was considered statistically significant in all analyses. In addition, multivariate analysis was performed using principal component analysis (PCA) with Unscrambler software (version 10.1, CAMO Software AS, Oslo, Norway) to explore patterns and clustering among samples. Prior to PCA, variables were preprocessed by averaging replicates and applying mean-centering to standardize the data, ensuring that all variables contributed equally to the analysis. Correlation analysis was carried out using Spearman’s rank correlation coefficient in SPSS to assess associations between variables. Furthermore, hierarchical cluster analysis was performed in Orange Data Mining (version 3.39.0, University of Ljubljana, Slovenia) using normalized data, Euclidean distance as the similarity measure, and average linkage as the clustering method, with the results displayed as dendrograms and clustered heat maps to identify similarity patterns among samples.

## 3. Results and Discussions

### 3.1. TPC of GP Polyphenol Extracts

The TPC of the 17 analyzed GP samples varied significantly among cultivars, decreasing in the following order: TR > JO > NE > FR > RE > RA> SH > SB > SE > MO > RB > RR > BR > AM > BL > AS > RU (Table 2).

Among these, TR, JO, and NE exhibited significantly higher TPC values compared to all other samples (Table 2). Conversely, the lowest statistically significant TPC values were observed in RU, AS, and BL (Table 2). Among the grapevine cultivars developed at SCDVV Blaj [16], RA showed the highest TPC, while RU had the lowest (Table 2). Within the red cultivars, RE recorded the highest TPC, whereas AM had the lowest (Table 2).

Several white grape cultivars grown in the Târnave and SCDVV Blaj vineyards, such as NE, TR, JO, and FR, contained higher TPC values compared to the red cultivars RE, SH, and AM (Table 2). This trend could be attributed to the climatic conditions of the Târnave region, which are particularly favorable for cultivating white grape varieties. In several years, relatively colder temperatures may not be sufficient for red cultivars to accumulate optimal levels of sugar and phenolic compounds [16].

In our dataset, the highest TPC for GP reached 73–79 mg GAE/g (e.g., TR, JO, NE, FR; Table 2), which positions these samples above typical apple pomace values (5–9 mg GAE/g; e.g., 5.78 mg GAE/g; 8.56 mg GAE/g; up to 22 mg GAE/g under optimized extraction) [47,48,49]. Relative to olive pomace, where TPC frequently falls in the 30–50 mg GAE/g range and extracts are notable for hydroxytyrosol/oleuropein enrichment, our top GP samples are broadly comparable on a TPC basis though with a distinct phenolic profile [50,51,52].

Berry pomaces provide an additional benchmark: blackcurrant pomace typically reports 24–37 mg GAE/g but can span 9–73 mg GAE/g depending on extraction, while blueberry pomace often falls around 13–17 mg GAE/g, placing our best GP lots at or above these ranges [53,54,55]. Pomegranate peel is an “gold-standard” by-product, often reporting very high TPC (≈100–300+ mg GAE/g) depending on cultivar and extraction; thus, even strong GP lots typically trail pomegranate peel in absolute TPC [56].

Overall, our data position GP as competitive with apple/citrus pomaces, broadly comparable to olive pomace (with different lead phenolics), and below pomegranate peel but with favorable availability and techno–economic feasibility for circular-economy valorization.

In the study conducted by De la Cerda-Carrasco et al. [57], GP samples from the Viña de Santa Alicia vineyard in the Maipo Valley, Chile, revealed that white GP varieties exhibited higher TPC than red varieties. This observation aligns with the understanding that red winemaking involves extended maceration, which facilitates a more complete extraction of polyphenols into the wine, leaving less in the pomace [57,58,59]. Notably, Sauvignon Blanc demonstrated significantly higher TPC compared to Chardonnay, Cabernet Sauvignon, and Carménère [57].

In contrast, white wine production typically excludes solid matter during fermentation, resulting in a greater retention of polyphenols in the grape skins and, consequently, in the pomace [60], thus resulting in higher concentrations of polyphenols left in white grape skins [57]. In a study published by Álvarez-Casas et al. [61], the concentration of polyphenolic compounds in GP derived from autochthonous white monovarietal *Vitis vinifera* grapes cultivated in Galicia (northwestern Spain) was found to range between 22 and 44 mg GAE/g dry weight of pomace.

The TPC values obtained for the Italian Riesling samples from Transylvania were consistent with those reported for the same cultivar in other regions. For example, Riesling GP from Moravia harvested in October 2018 contained 47.94 mg GAE/g [62], while white wine GP (*Vitis vinifera* L. cv. “Weisser Riesling”) from Baden-Baden-Neuweier, Germany, yielded a maximum TPC of 50.95 g GAE/kg dry material in 70% ethanol extract [63]. In another study, GP from Pinot Blanc and Riesling, as well as red cultivars such as Dornfelder, Pinot Noir, and Portugais Bleu from a wintery in Bingen (Rhineland-Palatinate), revealed TPC values ranging from 44 mg GAE/g (Riesling) to 65 mg GAE/g (Portugais Bleu) [64]. The Italian Riesling GP from Teremia Mare Winery in Western Romania (Timiș county) had a TPC of 92.99 mg GAE/g [65]. Similarly, samples from the Bajilo and Agner vineyards on the Fruška Gora Mountain in northern Serbia showed microclimate-dependent differences: 24.10 ± 0.13 mg GAE/g and 16.01 ± 0.43 mg GAE/g dried extract, respectively. Italian Riesling from Pietroasa-Isrița (southern Romania) had a TPC of 24.00 mg GAE/g [66].

Although the determined TPC values are in accordance with those reported in the literature, these may vary based on harvest year, terroir variability, and potential matrix interferences in the Folin–Ciocalteu assay. Limiting GP samples to a single harvest year means the TPC values may not represent the full range of possible phenolic contents across different years, potentially leading to an incomplete or biased understanding of a plant’s overall quality or medicinal potential. Terroir influences the composition of the GP, which in turn affects the total phenolic content measured by the Folin–Ciocalteu assay. Factors like climate, soil, and vineyard management, all components of terroir, significantly impact the types and amounts of phenolic compounds that accumulate in the grape, and subsequently in the pomace after winemaking [37]. TPC is also subject to matrix interferences in GP analysis, particularly from reducing sugars and ascorbic acid, which can cause an overestimation of total phenolic content. Other oxidizable compounds in the GP matrix can also interfere.

### 3.2. FTIR Analysis of GP Extracts

**Figure 1:**
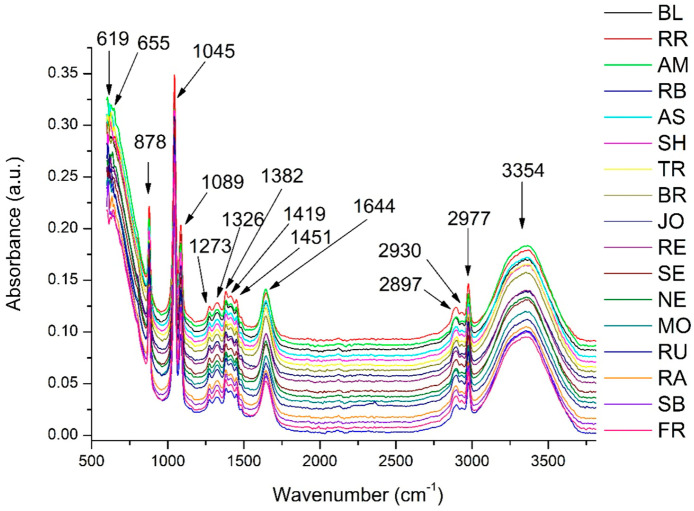
The Fourier transform infrared (FTIR) spectra of the 17 grape pomace (GP) extracts were recorded in the range of 650–3750 cm^−1^. The white grape cultivars analyzed included Blasius (BL), Rhine Riesling (RR), Roze Blaj (RB), Astra (AS), Traminer roz (TR), Johaniter (JO), Neuburger (NE), Rubin (RU), Sauvignon Blanc (SB), Feteascǎ Regalǎ (FR), Radames (RA), Brumăriu (BR), Selena (SE), and Muscat Ottonel (MO). The red grape cultivars included Regent (RE), Syrah (SH), and Amurg (AM).

Figure 1 presents the FTIR spectra of the 17 analyzed GP extracts. The observed absorption bands are consistent with those reported in previous studies [35,36,67,68,69,70]. The first broad absorption band, with a maximum at 3354 cm^−1^, is attributed to the O–H stretching vibrations of hydroxyl groups bonded to aromatic rings, characteristic of phenolic compounds [35,71].

In the spectral region of 3600 to 3200 cm^−1^, the FTIR spectra were found to mostly be attributed to the stretching vibrations of hydroxyl groups (-OH), indicating that alcohols and phenols are present [35,71]. This broad band also indicates that residual sugars are present [68,69], particularly in white GP, for which the traditional winemaking processes exclude pomace maceration, permitting more sugars to persist. Furthermore, the broad absorption from 3600 to 3000 cm^−1^ is associated with O–H stretching and N–H stretching vibrations, indicative of lignocellulosic components in the pomace [70,72].

The absorption bands in the 2980 to 2900 cm^−1^ range, with prominent peaks at 2977, 2930, and 2897 cm^−1^ (Figure 1), correspond to symmetric and asymmetric C–H stretching vibrations of aliphatic −CH_3_ and −CH_2_ groups [35,69,70,73,74]. These are commonly associated with lipid components and lignin-derived structures [36,70,75].

The bands at 1644, 1451, and 1419 cm^−1^ (Figure 1) are associated with aromatic skeletal vibrations [69]. The 1644 cm^−1^ band could also be attributed to asymmetric and symmetric stretching of the carboxylate groups (−COO^−^, often found in hydroxybenzoic acids present in GP [35,73,76]. Peaks between 1320 and 1460 cm^−1^ are associated with non-symmetric scissoring and bending vibrations of CH_3_ groups from aliphatic compounds [77].

In the 1280 to 1000 cm^−1^ region, the observed peaks correspond to C–O stretching vibrations in water-soluble components such as polysaccharides [36,69,70] and to C−6 vibrations of cellulose [70,72,78].

A sharp peak at 878 cm^−1^ (Figure 1) is attributed to C–H out-of-plane deformation in pyranoside rings and mannose structures [35,71]. This is consistent with the known composition of GP, which includes monosaccharides such as rhamnose, xylose, mannose, arabinose, galactose, glucose, and uronic acids [10].

**Figure 2:**
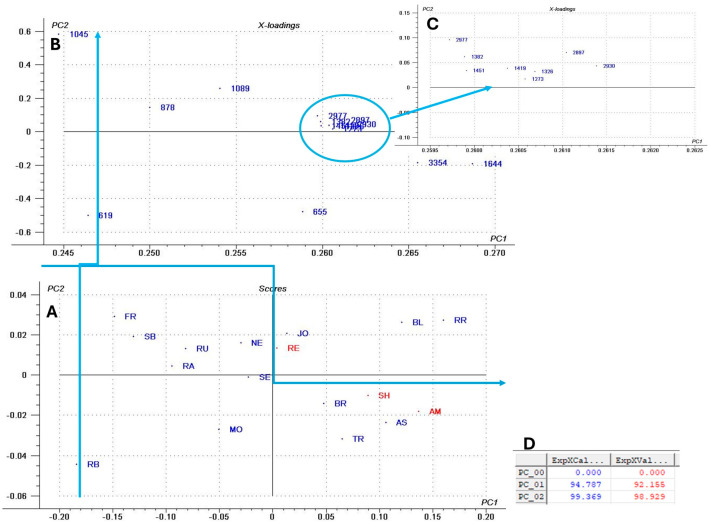
Score plot of the first two principal components, PC1 and PC2 (**A**), based on the peak areas identified using Fourier transform infrared (FTIR) analysis of the 17 grape pomace extracts; (**B**) loading plot of PC1 and PC2 showing how the analyzed peak areas contribute to grape pomace samples distribution among the principal component; (**C**) zoom of loading variables in the loading score plot; (**D**) explained variance of PCA model. The peak areas considered for PCA analysis were 619 cm^−1^; 655 cm^−1^; 878 cm^−1^; 1045 cm^−1^; 1089 cm^−1^; 1273 cm^−1^; 1326 cm^−1^; 1382 cm^−1^; 1419 cm^−1^; 1451 cm^−1^; 1644 cm^−1^; 2897 cm^−1^; 2930 cm^−1^; 2977 cm^−1^, and 3354 cm^−1^. The white grape cultivars analyzed included Blasius (BL), Rhine Riesling (RR), Roze Blaj (RB), Astra (AS), Traminer roz (TR), Johaniter (JO), Neuburger (NE), Rubin (RU), Sauvignon Blanc (SB), Feteascǎ Regalǎ (FR), Radames (RA), Brumăriu (BR), Selena (SE), and Muscat Ottonel (MO). The red grape cultivars included Regent (RE), Syrah (SH), and Amurg (AM).

Next, a multivariate analysis was performed using principal component analysis (PCA) (Figure 2B) based on the area of previously identified peaks to determine which variables most influenced GP discrimination. The PCA score plot (Figure 2B) of the first two principal components (PCs) revealed a clear separation between GP varieties, explaining 99% of the total sample variance. The PCA loading plots (Figure 2B,C) identified the bands at 1644 cm^−1^, 3354 cm^−1^, and 655 cm^−1^ as the main contributors to clustering along the PC1 axis. Higher area values of these peaks (positive correlation) characterized the RR, AM, BL, SH, BR, and TR varieties, whereas lower area values (negative correlation) were associated with RB, FR, and SB. As previously discussed, the band at 1644 cm^−1^ corresponds to carboxylate groups (–COO^−^), typically linked to hydroxybenzoic acids present in GP. The broad absorption between 3600 and 3000 cm^−1^ reflects O–H and N–H stretching vibrations, indicative of lignocellulosic components in the pomace. Finally, the peak at 655 cm^−1^ is assigned to C–H out-of-plane bending vibrations of aromatic rings, which are characteristic of phenolic compounds.

These discriminating bands emphasize the central role of phenolic compounds, particularly aromatic structures and carboxylate groups, in driving varietal separation. Although polysaccharide and lignocellulosic contributions are evident, the predominance of phenolic-associated peaks highlights their importance.

### 3.3. HPLC–DAD–ESI MS Analysis of GP Extracts

The LC–MS analysis (Figures S2 and S3) revealed the phenolic compound profiles of the 17 pomaces. Four types of phenolic compounds were identified: phenolic acids, flavanols (Table S2), flavonols, and anthocyanins (Table S3). Hydroxybenzoic and hydroxycinnamic acids are the two types of phenolic acids generally found in grapes, wine, and GP. In this study, only two hydroxybenzoic acids, 2-hydroxybenzoic acid and gallic acid, were identified and quantified in the 17 pomaces (Table S2). RE has the highest content of hydroxybenzoic acid (173.2 μg/mL), followed by NE (144.6 μg/mL) (Table S2). The lowest concentration of hydroxybenzoic acid (51.2) was found in MO GP extract (Table S2). NE has the highest content of gallic acid (59.1 μg/mL), followed by JO (41.9 μg/mL) (Table S2). Four GP extracts, RR (14.2 μg/mL), RB (15.9 μg/mL), BL (14.9 μg/mL), and SE (15.1 μg/mL) had the lowest concentrations of gallic acid (Table S2). NE has the highest content of total phenolic acids (203.7 μg/mL), followed by RE (199.3 μg/mL) (Table S2). The lowest concentration of total phenolic acids (69.1 μg/mL) was found in MO GP extract (Table S2). In our previous study on Muscat Ottonel grapes from the Blaj, Târnave vineyard, 2-hydroxybenzoic acid was also found together with the gallate of gallic acid [37]. Other than these, three hydroxycinnamic acids, chlorogenic, caffeic, and ferric acids, were separated [37].

Only hydroxycinnamic, caftaric, and coutaric acids, constituted the non-flavonoid components in Spanish Moscatel grapes [79]. Cheng et al. [80] identified and quantified only one hydroxybenzoic acid, the hexose ester of vanillic acid, in the grape berry skins of an Italian Riesling cultivar from a Chinese vineyard [80]. In Riesling grapes from Serbia, five hydroxybenzoic acids were identified, as well as two hydroxycinnamic acids (chlorogenic and caffeic acid) in seeds; gallic acid, protocatechuic acid, and ellagic acid in skin samples and three hydroxybenzoic acids (chlorogenic, caffeic, and ferric acid); and three hydroxycinnamic acids (gallic, protocatechuic and gentisic) in pulp [81]. Concentrations of gallic acid in the Serbian Riesling seeds were 54.66 mg/kg dry weight, 4.47 mg/kg dry weight in skins, and 0.49 mg/kg dry weight in pulp [81].

Flavan-3-ols were identified as the most abundant phenolic compounds in the GP extracts, consistent with previously published findings [14,23,24,66,82]. In Italian Riesling pomace from wineries located on the Fruška Gora Mountain in northern Serbia, only catechin and epicatechin were reported, with concentrations of 139 ± 0.85 mg/kg fresh weight and 132 ± 1.19 mg/kg for the Agner winery and 110 ± 1.08 mg/kg fw and 94.7 ± 0.76 mg/kg fresh weight for the Bajilo winery, respectively [66].

In contrast, this present study identified a broader spectrum of flavan-3-ols across all GP samples, including catechin, epicatechin, epicatechin gallate, a catechin derivative, and three pyocyanidin dimers (B1, B2, and B3). Although their concentrations varied significantly, all extracts contained these compounds.

SB exhibited the highest concentration of procyanidin dimer B1 (40.8 μg/mL), followed by FR (37.3 μg/mL), RE (34.6 μg/mL), and BL (31.3 μg/mL) (Table S2). The lowest levels were found in MO (17.4 μg/mL) and SE (17.7 μg/mL) (Table S2). RE had the highest concentration of procyanidin dimer B2 (134.3 μg/mL), with BL (40.4 μg/mL) and RU (40.1 μg/mL) also showing somewhat high concentrations (Table S2). The lowest concentrations of B2 were observed in RB (11.9 μg/mL), AS (13.5 μg/mL), MO (13.7 μg/mL), and AM (14.7 μg/mL) (Table S2). SH had the highest concentration of procyanidin dimer B3 (42.1 μg/mL), followed by RE (40.1 μg/mL), JO (39.0 μg/mL), BL (37.8 μg/mL), and NE (37.3 μg/mL) (Table S2). The lowest B3 concentrations were observed in AS (15.7 μg/mL), AM (15.7 μg/mL), and MO (15.9 μg/mL) (Table S2).

NE had the highest catechin content with a concentration of 217.9 μg/mL, followed by RA (205.9 μg/mL), while SE had the lowest concentration (32.8 μg/mL) (Table S2). NE also had the highest epicatechin concentration (279.2 μg/mL), followed by RA (230.3 μg/mL) and BR (220.1 μg/mL) (Table S2). The lowest epicatechin concentrations were observed in MO (55.9 μg/mL) and SE (58.9 μg/mL) (S1). RA exhibited the highest epicatechin gallate content (236.2 μg/mL), followed by BR (112.0 μg/mL), with MO showing the lowest (18.6 μg/mL) (Table S2). NE also had the highest concentration of catechin derivative (398.0 μg/mL), followed by RE (293.2 μg/mL), while AS had the lowest (86.8 μg/mL) (S1). Regarding total flavan-3-ol content, NE again ranked highest (1019.4 /mL), followed by RA (936.2 μg/mL), RE (866.3 μg/mL), and BR (730.4 μg/mL) (S1). The lowest concentration of flavan-3-ol was found in MO, which contained 326.5 μg/mL (Table S2).

RE exhibited the highest concentration of quercetin–glucoside (16.5 μg/mL), followed by AS (12.4 μg/mL) (Table S3). In contrast, the lowest concentrations were detected in the SE and RA extracts, both of which contained 17.5 μg/mL. The highest kaempferol-glucoside content was observed in AS (5.9 μg/mL) and AM (5.7 μg/mL), while the lowest concentrations were found in BL (0.03 μg/mL) and RR (0.09 μg/mL) (Table S3). Regarding total flavonol content, RE again showed the highest value (21.1 μg/mL), followed by AS (18.4 μg/mL). The lowest total flavonol concentration was recorded in SE (2.0 μg/mL) (Table S3).

Four anthocyanins were identified in the red GP extracts: malvidin–caffeoyl–glucoside, malvidin–glucoside, malvidin–acetyl–glucoside, and malvidin–coumaroyl–glucoside. Malvidin–caffeoyl–glucoside was detected exclusively in RE, with a concentration of 6.2 (Table S3). RE also exhibited the highest levels of malvidin–glucoside (6.1 μg/mL), malvidin–acetyl–glucoside (5.0 μg/mL), and malvidin–coumaroyl–glucoside (10.1 μg/mL), followed by AM, which contained 4.9 μg/mL, 3.6 μg/mL, and 4.5 μg/mL of these compounds, respectively (Table S3).

Although SE is classified as a white grape cultivar in this study, due to its rose-colored skin, malvidin–coumaroyl–glucoside was also detected in its pomace at a concentration of 3.2 μg/mL (Table S3). Regarding the total anthocyanin content, RE had the highest concentration (27.4 μg/mL), followed by AM (13.0 μg/mL) and SH (9.5 μg/mL). The lowest total anthocyanin content was found in SE (3.2 μg/mL) (Table S3).

The flavonol composition of grape skins is known to vary significantly depending on cultivar and growing conditions [83,84]. Italian Riesling berry skin cultivated in Manas County, Shihezi City, in the wine-producing region of Xinjiang Province, P. R. China, had a percentage of quercetin derivatives (88.37%) and kaempferol derivatives (11.63%) [80] in accordance with our actual results. Mattivi et al. [85] also reported that quercetin was the predominant flavonol in white cultivars, with a mean content of 81.35% (range: 72.46–96.90%), followed by kaempferol (mean: 16.91%; range: 2.33–26.34%) and isorhamnetin (mean: 1.74%; range: 0–5.38%) [85]. Similarly, Castillo-Muñoz et al. [86] found that quercetin derivatives represented 60.8–90.7% (mean: 77.2 ± 7.3%) of total flavonols, kaempferol ranged from 8.8 to 38.3% (mean: 21.4 ± 7.4%), and isorhamnetin averaged 1.5 ± 1.2% [86]. Contrary to the above findings, the Muscat Ottonel from Blaj (Târnave vineyard, Romania) revealed a different flavonol profile, with quercetin derivatives comprising only 50.84% and kaempferol comprising 11.00%, with a notably elevated isorhamnetin content of 38.16% [37]. These compounds were only identified in skin samples, and they were absent in seeds [37]. Flavonols such as quercetin and isorhamnetin accumulate in grape skins, where they serve as UV–B protective agents by blocking wavelengths that damage DNA through their strong photoprotective properties [87,88].

**Figure 3:**
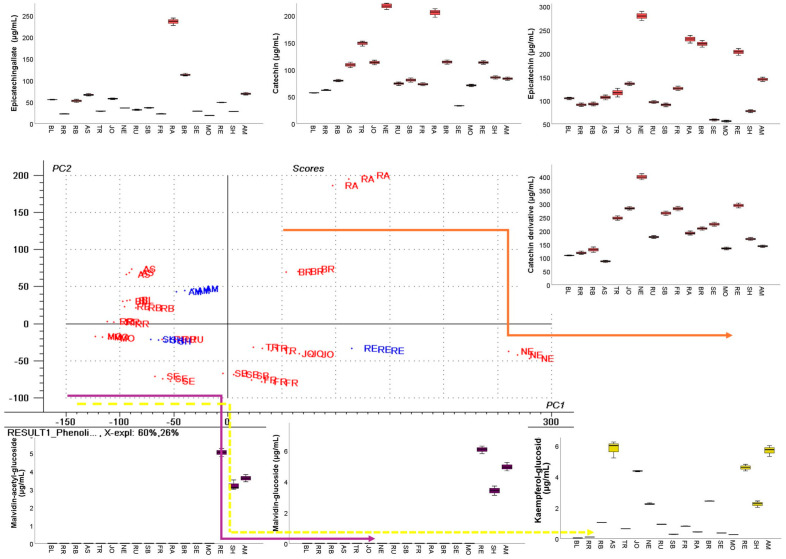
Score plot of the first two principal components (PC1 and PC2) based on the phenolic compound concentrations determined by HPLC−DAD−ESI MS in 17 grape pomace extracts. Loadings are represented on the PCA plot to indicate the contribution of individual phenolic compounds to sample distribution using boxplots. Boxplots illustrate the main discriminating compounds: flavanols (catechin derivative, epicatechin, catechin, and epicatechin gallate; orange line), which influenced separation along the right side of PC1; anthocyanins (malvidin−acetyl−glucoside and malvidin−glucoside; purple line), which influenced separation along the left side of PC1; and the flavonol kaempferol−glucoside (yellow line), also contributing to separation along the left side of PC1. The analyzed samples included fourteen white grape cultivars—Blasius (BL), Rhine Riesling (RR), Roze Blaj (RB), Astra (AS), Traminer roz (TR), Johaniter (JO), Neuburger (NE), Rubin (RU), Sauvignon Blanc (SB), Feteascǎ Regalǎ (FR), Radames (RA), Brumăriu (BR), Selena (SE), and Muscat Ottonel (MO)—and three red grape cultivars—Regent (RE), Syrah (SH), and Amurg (AM).

In our dataset, flavan-3-ols (catechin, epicatechin, epicatechin gallate, procyanidin B1, B2, B3) dominate GP extracts across cultivars but flavonols are also present, although in lower concentrations, and red pomaces carried anthocyanins too (Tables S2 and S3). These patterns match other reviewed GP profiles [23], where catechin, epicatechin, and B-type procyanidins occur in other Romanian and Argentinian GPs [7,89]. The PCA multivariate analysis (Figure 3) also highlights that the sample distribution along the PC1 and PC2 score plot is influenced mostly by the flavanols.

Accordingly, cultivars NE, RA, RE, and BR were positively correlated with increased concentrations of the catechin derivative, epicatechin, catechin, and epicatechingallate, while the negatively correlated cultivars like MO, RR, BL, RB, and AS had lower concentrations of these compounds.

### 3.4. GC–FID Analysis of GP Extracts

In total, twenty-two different fatty acids were identified and quantified in the analyzed GP samples, as shown in Table S4. The ten saturated fatty acids (SFAs) identified included caprylic acid (C8:0), capric acid (C10:0), lauric acid (C12:0), myristic acid (C14:0), pentadecanoic acid (C15:0), palmitic acid (C16:0), heptadecanoic acid (C17:0), stearic acid (C18:0), arachidic acid (C20:0), and lignoceric acid (C24:0) (Table S4). In addition, three monounsaturated fatty acids (MUFAs) were identified: penadecenoic acid (C15:1), palmitoleic acid (C16:1), and oleic acid (C18:1) (Table S4). The polyunsaturated fatty acids (PUFAs) consisted of nine compounds, including four ω-3 fatty acids, specifically, linolenic acid (C18:3n3), octadecatetraenoic acid (C18:4n3), eicosatrienoic acid (C20:3n3), and eicosapentaenoic acid (C20:5n3), as well as five ω-6 fatty acids, which included cis-linoleic acid (C18:2n6), eicosadienoic acid (C20:2n6), eicosatrienoic acid (C20:3n6), arachidonic acid (C20:4n6), and docosadienoic acid (C22:2n6) (Table S4).

This profile of fatty acids is more comprehensive than those presented in previously published works. For example, Ferreira et al. [90] identified three SFAs, two MUFAs, and two PUFAs, while Carmona-Jiménez et al. [25] declared that they detected six SFAs, three MUFAs, and two PUFAs. This present study therefore highlights the complex lipid composition of GP and underscores its potential as a valuable source of bioactive fatty acids and benefits to human health.

Palmitic acid (C16:0) was the most abundant SFA identified across the different GP samples, with the highest concentration observed in BL (15.53%) and the lowest in AS (8.92%) and RB (9.07%) (Table S4). Among all samples, BL also exhibited the highest total SFAs content (21.03%), while RB had the lowest (13.55%) (Table S4). These findings are significant, considering that a high dietary intake of SFAs, particularly palmitic acid, is associated with promoting inflammation and contributing to the development of obesity-related disorders and insulin resistance [90,91].

**Figure 4:**
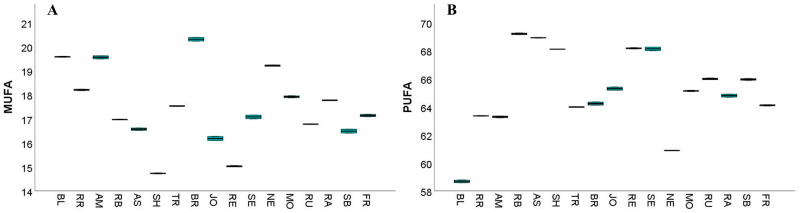
The median values of monounsaturated (MUFA) (**A**) and polyunsaturated (PUFA) (**B**) fatty acids were quantified in the 17 white grape pomace cultivars. In the box plot, the box boundaries correspond to the first (Q1) and third (Q3) quartiles, the central line denotes the median, and the lines extending from the box indicate the minimum and maximum values outside the interquartile range. The analyzed white grape pomace cultivars were Blasius (BL), Rhine Riesling (RR), Roze Blaj (RB), Astra (AS), Traminer roz (TR), Johaniter (JO), Neuburger (NE), Rubin (RU), Sauvignon Blanc (SB), Feteascǎ Regalǎ (FR), Radames (RA), Brumăriu (BR), Selena (SE), and Muscat Ottonel (MO). The red grape cultivars included Regent (RE), Syrah (SH), and Amurg (AM).

The content of unsaturated fatty acids (UFAs) varied between 78.25% in BL and 86.15% in RB (Table S4), aligning with the results previously reported in a study by Carmona-Jiménez et al. [25], who observed an average UFAs content of 83.41% in five Spanish GP samples. MUFAs ranged from 14.73% in SH to 20.30% in BR, while PUFAs were present in concentrations ranging from 58.67% in BL to 69.19% in RB (Table S4, Figure 4A). Furthermore, high PUFAs levels were also observed in AS (68.91%), RE (68.16%), and SH (68.09%) (Table S4, Figure 4B). Therefore, these values are comparable to those previously reported for Spanish GPs, where MUFA content ranged from 16.43% to 18.90% and PUFA content ranged from 64.04% to 66.87% [25].

Previous publications have stated that GP typically contains low quantities of MUFAs, ranging from 14% to 19%, and SFAs, which range between 11% and 12%, while also containing a high content of PUFAs, which account for 69% to 75% of the total fatty acid content. The high PUFA content is attributed to the high quantities of cis-linoleic acid [90,92,93,94,95].

Similarly, this present study found cis-linoleic acid was the predominant fatty acid in all the analyzed GP samples, with concentrations ranging from 55.68% in BL to 67.86% in RB (S3). These values are similar to the values reported for Spanish GPs, where cis-linoleic acid levels ranged between 61.37% and 65.16% [25]. Notably, the red cultivars, including RE, SH, and AM, exhibited slightly higher cis-linoleic acid concentrations than the white cultivars, a trend similar to that observed in the Spanish GP samples studied by Carmona-Jiménez et al. [25].

Oleic acid is the principal MUFA and the second most abundant fatty acid in the analyzed samples, present in concentrations ranging from 13.63% in SH to 18.17% in BR (S3). These values are consistent with those reported by Carmona-Jiménez et al. [25], who found that oleic acid levels were present in concentrations between 16.04% and 18.87% in Spanish GPs.

Linolenic acid, an important ω-3 PUFA, was also detected in all samples, with concentrations ranging from 0.64% in AS to 1.87% in AM. In comparison to the average linolenic acid content in grape seed oil, which commonly ranges between 0.30% and 0.40% [25,90,94,96,97,98,99,100,101,102,103], the quantities found in GP are higher. Carmona-Jiménez et al. [25] described linolenic acid concentrations in Spanish GPs ranging between 0.90% and 4.30%, while Ferreira et al. [90] described a mean value of 1.94% ± 0.32% in Portuguese GP samples.

**Figure 5:**
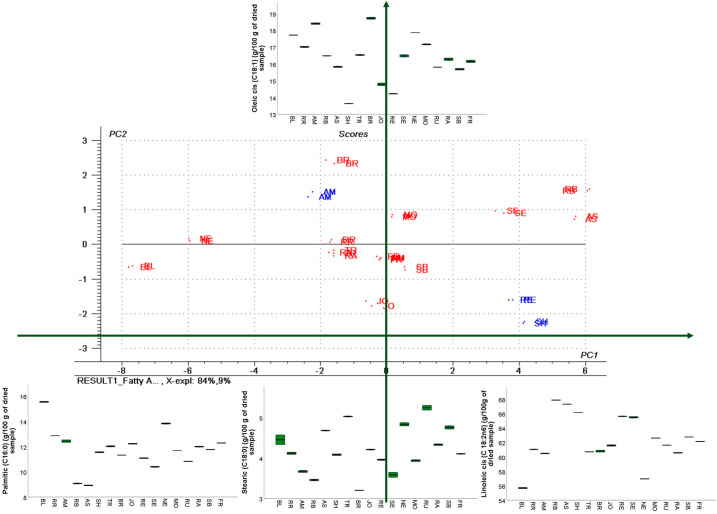
Score plot of the first two principal components (PC1 and PC2) based on the fatty acid concentrations determined with GC−MS in 17 grape pomace extracts. Loadings are represented on the PCA plot to indicate the contribution of individual fatty acid to sample distribution using boxplots. Boxplots illustrate the main discriminating compounds—cis linoleic, stearic, and palmitic acids—that influenced separation along the horizontal PC1 axis and oleic acid, which influenced separation along the vertical PC2 axis. The analyzed samples included 14 white grape cultivars—Blasius (BL), Rhine Riesling (RR), Roze Blaj (RB), Astra (AS), Traminer roz (TR), Johaniter (JO), Neuburger (NE), Rubin (RU), Sauvignon Blanc (SB), Feteascǎ Regalǎ (FR), Radames (RA), Brumăriu (BR), Selena (SE), and Muscat Ottonel (MO)—and three red grape cultivars—Regent (RE), Syrah (SH), and Amurg (AM).

These findings are also supported by the multivariate PCA analysis (Figure 5), which showed that oleic and cis linoleic acids are the principal compounds, as evidenced by the corresponding loading plots that influenced sample distribution along the PC1 and PC2 axis.

The proportion of linolenic acid in GP oil was found to be between three to ten times higher than in corresponding grape seed oils, as previously reported by Carmona-Jiménez et al. [25]. This is particularly notable given that very few vegetable oils contain linolenic acid in appreciable amounts. According to Orsavová et al. [104], the highest linolenic acid content was found in wheat germ, rapeseed, and olive oils, ranging between 1.2% and 1.6%. Bondioli et al. [105] also noted that among commercially available oils, only soybean and rapeseed oils contain up to 10% linolenic acid [25]. However, a high concentration of PUFAs, such as linolenic acid, can increase the susceptibility of oils to oxidation, potentially compromising their stability and promoting their degradation [25,106].

**Figure 6:**
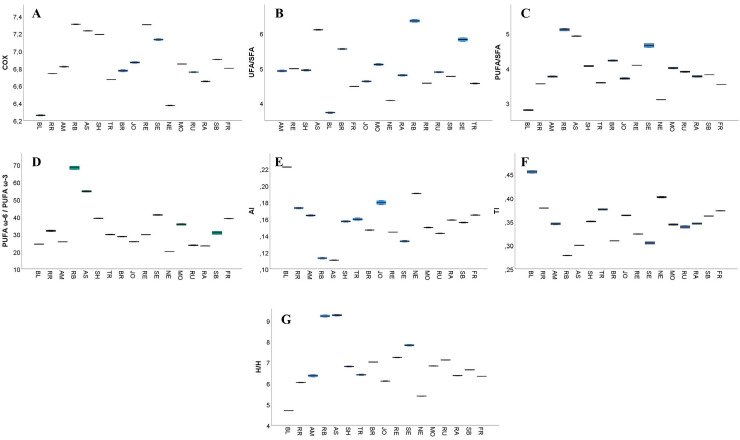
The calculated oxidizability (COX) (**A**), unsaturation ratio (UFA/SFA) (**B**), PUFA/SFA ratio (**C**), ratio of ω-6 to ω-3 polyunsaturated fatty acids (PUFA ω-6/PUFA ω-3) (**D**), atherogenicity index (AI) (**E**), thrombogenicity index (TI) (**F**), and hypo and hypercholesterolemic fatty acids ratio (H/H) (**G**) of the 17 grape pomace extracts. The box plots illustrate the distribution of these indices, where the box boundaries correspond to the first (Q1) and third (Q3) quartiles, the central line indicates the median, and the extending lines represent the minimum and maximum values. The white grape cultivars analyzed included Blasius (BL), Rhine Riesling (RR), Roze Blaj (RB), Astra (AS), Traminer roz (TR), Johaniter (JO), Neuburger (NE), Rubin (RU), Sauvignon Blanc (SB), Feteascǎ Regalǎ (FR), Radames (RA), Brumăriu (BR), Selena (SE), and Muscat Ottonel (MO). The red grape cultivars included Regent (RE), Syrah (SH), and Amurg (AM).

To assess the oxidative stability of the GP fatty acids, the calculated oxidizability (COX) values were determined (Figure 6A).

The profile of unsaturation ratio (UFA/SFA) (Figure 6B), PUFA/SFA ratio (Figure 6C), atherogenicity index (AI) (Figure 6E), thrombogenicity index (TI) (Figure 6F), and hypo and hypercholesterolemic fatty acids ratio (H/H) (Figure 6G) are also presented in Table S4. The COX values for the studied samples ranged from 6.26% in BL to 7.67% in TR (Table S4), aligning with those previously reported for Spanish GP samples (6.87 ± 0.08% to 7.86 ± 0.04%) [25]. As previously discussed, the fatty acid composition of oils extracted from winemaking by-products may serve as indicators of their nutritional and functional properties [25]. One indicator is the unsaturated-to-saturated fatty acid ratio (UFA/SFA), which reflects the balance between health-promoting unsaturated fats and potentially harmful saturated fats [90]. In this present study, UFA/SFA ratios ranged between 3.72 in BL and 6.36 in RB (Table S4), similar to the values reported for Spanish (4.78–6.04) and Portuguese (5.55) GP samples [25,90].

Another critical nutritional index is the PUFA/SFA ratio, which is widely recognized as a reliable measure of dietary fat quality. The British Department of Health recommends a minimum PUFA/SFA ratio of 0.45 for a healthy human diet [25,107]. All GP samples in this study exceeded this threshold, with PUFA/SFA ratios ranging from 2.79 in BL to 5.11 in RB (Table S4). These values are consistent with those reported by Carmona-Jiménez et al. [25], who observed PUFA/SFA ratios between 3.82 and 4.73 in Spanish GP samples.

The ratio of ω-6 to ω-3 polyunsaturated fatty acids (PUFA ω-6/PUFA ω-3) is also important to consider, as optimal health benefits are achieved when the intake of these two fatty acid families is balanced [25,104]. In this study, the PUFA ω-6/PUFA ω-3 ratio ranged from 20.01 in NE to 68.11 in RB (Table S4), with the Portuguese GP sample reported by Ferreira et al. [90] showing a ratio of 37.3. Western diets often include an excessive intake of ω-6 fatty acids and an insufficient intake of ω-3 fatty acids, resulting in an imbalance in the PUFA ω-6/PUFA ω-3 ratio [25]. This imbalance has raised interest in oils and lipid-rich matrices with higher ω-3 content [25,102]. It has been demonstrated that using whole GP for oil extraction significantly increases the linolenic acid content and improves the PUFA ω-6/PUFA ω-3 ratio when compared to using seeds alone [25]. It is also important to note that among the cultivars studied, RB in particular was found to contain high concentrations of both UFAs and PUFAs.

The AI and TI are important indicators for assessing the potential impact of dietary fats on cardiovascular health. In this present study, AI values ranged from 0.11 in RB and AS to 0.22 in BL, while TI values varied from 0.28 in RB to 0.45 in BL (Table S4). These values are similar to those reported for Spanish GP, where AI ranged from 0.11 to 0.16 and TI from 0.30 to 0.35, and for grape seed oil, where AI ranged from 0.09 to 0.11 and TI from 0.28 to 0.31 [25]. Similarly, Portuguese GP exhibited an AI of 0.11 and a TI of 0.30 [90]. The AI values observed in RB and AS (S3) were higher compared to those for linseed oil but lower than those for olive and sesame oils, and TI was higher than the one reported in the literature for linseed and sesame oils but lower than that one of olive oil [108]. In Serbian grape seed oils, AI values ranged from 0.08 to 0.09 for both red and white grape varieties, while TI values ranged from 0.25 to 0.27 for red grape seed oil and 0.26 to 0.27 for white grape seed oil [94]. Elevated AI and TI values are associated with a greater risk of atherogenicity and thrombogenicity, making these indices valuable for assessing the cardiovascular implications of dietary fats [90,109].

The hypocholesterolemic/hypercholesterolemic fatty acid ratio (H/H) is another key nutritional index that reflects the influence of fatty acids on cholesterol metabolism. In this study, H/H values ranged from 4.69 in BL to 9.25 in AS, with a similarly high value observed in RB (9.22) (Table S4). These results are consistent with those reported for Portuguese GP (8.69) and Spanish GP (6.93–9.45) [25,90]. Higher H/H values are considered beneficial for cardiovascular health. However, the values obtained during this study were lower in comparison to those reported for linseed oil (13.24), some Spanish grape seed oils (10.54), and Serbian grape seed oils, which ranged from 11.07 to 12.28 for red grape oils and 11.30 to 12.09 for white grape oils [94,108]. Notably, cultivars such as AS, RB, and SE exhibited higher H/H values than those reported for sesame (7.72) and olive oils (6.14) [108].

The UFA/SFA ratio and the PUFA ω-6/PUFA ω-3 ratio are also widely used to assess the nutritional quality of fats. According to Ahmed et al. [110], a low UFA/SFA ratio and a high PUFA ω-6/PUFA ω-3 ratio are undesirable, as they may contribute to elevated cholesterolemia. Simopoulous [111] further emphasized that an imbalanced PUFA ω-6/PUFA ω-3 ratio is associated with an increased risk of obesity. In addition, elevated AI and TI values are not favorable for cardiovascular health [90]. In this context, the RB and AS cultivars exhibited the most favorable profiles, exhibiting the lowest AI and TI values and the highest H/H and UFA/SFA ratios (Table S4), suggesting a potentially positive impact on cardiovascular health. High H/H and UFA/SFA values are particularly desirable in nutrition, as they reflect a beneficial influence of fatty acids on cholesterol metabolism [90]. The largest analysis to date [112] examining the association of circulating saturated and unsaturated fatty acids challenges the current broad dietary recommendations that focus solely on reducing overall saturated fat intake, confirming that associations between cardiovascular diseases and different types of saturated fatty acids vary significantly.

### 3.5. Antioxidant Capacity

The antioxidant capacity of the extracts was performed based on the different possible mechanisms reported in the literature—radical scavenging, electron transfer, and complementarity by chelation of transition metals (Fe^2+^ and Cu^2+^) [113,114,115,116].

The antioxidant activity of the extract can be attributed to both the abundance of molecules with antioxidant properties and the intrinsic antioxidant properties of the molecules present in the mixture. Because plant extracts are often complex mixtures of compounds, we evaluated the antioxidant potential of the extracts in this research using several methods, given the phytochemically complex nature of the extracts. Another reason why it is advisable to apply several methods to evaluate antioxidant properties is that the applied tests use different experimental conditions, such as pH, temperature, solvent, redox potential, and type of radical. Moreover, previous reports have presented noticeable differences between the results obtained by applying two or more evaluation methods to the same analyzed extract [117,118,119,120].

#### 3.5.1. Antiradical, Electron Transfer, and Chelation Assays

To assess the antioxidant activity of the GP extracts, multiple assays employing different mechanisms of action were performed. These included radical scavenging assays (ABTS˙^+^ and DPPH˙), as well as electron transfer-based assays such as CUPRAC, FRAP, and reducing power (RP). Given the role of transition metal ions, particularly Fe^2+^ and Cu^2+^, in catalyzing free radical formation in biological systems, the metal-chelating capacity of the extracts was also assessed. This mechanism is considered a complementary antioxidant pathway.

##### Antiradical Assays

The antiradical activity of the GP extracts was determined spectrophotometrically by measuring their ability to scavenge DPPH˙ and ABTS˙^+^ radicals, with the results summarized in Table S5. Among the samples, the NE extract exhibited the strongest antiradical activity in both assays, with values of 115.45 µM TE/mL extract for DPPH˙ and 3.87 mg GAE/mL extract for ABTS˙^+^ (Table S5). This extract demonstrated the highest radical scavenging capacity.

**Figure 7:**
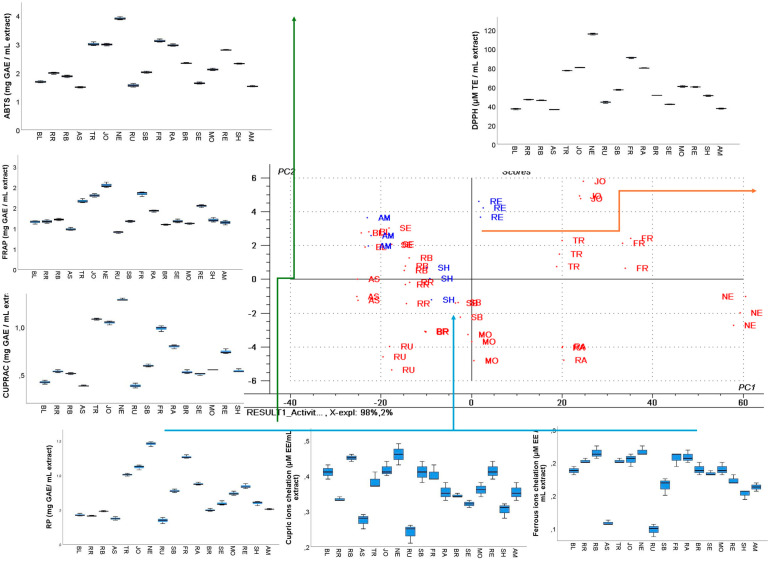
Score plot of the first two principal components (PC1 and PC2) based on the antioxidant activities as determined by (DPPH) radical scavenging, 2,2′−azino−bis (3−ethylbenzothialzoline−6−sulfonic acid) (ABTS) radical scavenging, ferric−reducing antioxidant power (FRAP), cupric ion−reducing antioxidant capacity (CUPRAC), reducing power (RP) assays, cupric ion chelation, and ferrous ion chelation in 17 grape pomace extracts. Loadings are represented on the PCA plot to indicate the contribution of individual phenolic compounds to sample distribution using boxplots. Boxplots illustrate the main discriminating antioxidant activities: DPPH (orange line), which influenced separation along the right side of PC1; ABTS, FRAP, and CUPRAC (green line), which influenced separation along the vertical PC2 axis; and RP, cupric, and ferrous ion chelation (blue line), which also contribute to separation along the left side of PC1. The analyzed samples included 14 white grape cultivarss—Blasius (BL), Rhine Riesling (RR), Roze Blaj (RB), Astra (AS), Traminer roz (TR), Johaniter (JO), Neuburger (NE), Rubin (RU), Sauvignon Blanc (SB), Feteascǎ Regalǎ (FR), Radames (RA), Brumăriu (BR), Selena (SE), and Muscat Ottonel (MO)—and three red grape cultivars—Regent (RE), Syrah (SH), and Amurg (AM).

The FR extract also demonstrated strong activity in both DPPH˙ (90.76 TE/mL) and ABTS˙^+^ (3.09 mg GAE/mL) assays (Table S5). In contrast, the lowest DPPH˙ scavenging activities were observed in AS (36.23 µM TE/mL) and BL (36.93 µM TE/mL), while the weakest ABTS˙^+^ neutralization capacities were recorded for AS (1.49 mg GAE/mL), AM (1.51 mg GAE/mL), and SE (1.62 mg GAE/mL) (Table S5). These results are further supported by the PCA analysis (Figure 7), which revealed that the distribution along the PC1 and PC2 axes, which explained 98% of the total variance, was mostly influenced by the DPPH and ABTS activities. It can be observed that cultivars like NE, FR, JO, TR, and RA are positively correlated with DPPH activity, as observed by the groups plotted along the positive PC1 axis, while AS, BL, and AM are positively correlated with ABTS activity, as observed by the groups plotted along the PC2 axis (Figure 7).

##### Electron Transfer Assays

To assess the electron-donating capacity of the GP extracts, three assays were performed: CUPRAC, FRAP, and RP. These assays were performed following different experimental conditions, providing a broader insight into the antioxidant activity of the samples. The results of the electron transfer assays, which were applied on the studied pomace extracts, are presented in Table S5.

Both the FRAP and RP assays rely on the reduction of ferric ions (Fe^3+^) to ferrous ions (Fe^2+^), though they differ substantially in their reaction environments and oxidants. The FRAP assay is performed at an acidic pH of 3.7 and at room temperature (≈20 °C), where the generated Fe^2+^ forms a blue complex with 2,4,6-tris(2-pyridyl)-s-triazine. In contrast, the RP assay performs best under near-neutral conditions (0.2 M phosphate buffer, pH 6.6) and requires mild heating at 50 °C. In this process, Fe^3+^ is replaced by [Fe(CN)^6^]^3−^ as the oxidant and causes the resulting Fe^2+^ to form Perl’s Prussian blue. The redox potential of the Fe^3+^/Fe^2+^ couple is 0.77 eV, while that of [Fe(CN)_6_]^3−^/[Fe(CN)_6_]^4−^ is significantly lower at 0.37 eV, indicating a weaker oxidizing power of the oxidizing agent from the RP assay [121,122].

Because of the complexation of the ferric ion with six cyanide strong-field ligands in [Fe (CN) 6]^3−^, it leads to a significant difference between oxidation potentials of the two aforementioned oxidizing agents—about 0.4 V—which influences the degree of oxidation of the molecules found in samples, as presented in literature reports. Therefore, Fe^3+^, being a stronger oxidizing agent, will oxidize in the FRAP assay both strong and low antioxidant compounds found in the studied extracts, while [Fe (CN) 6]^3−^ will oxidize in the RP assay only the strong antioxidant compounds found in the studied extracts [123,124,125,126]. In the CUPRAC assay, Cu^2+^ is reduced to Cu^+^, which then forms a stable orange complex with neocuproine. The intensity of this complex is directly proportional to the antioxidant capacity of the sample.

Across all three assays, the NE extract demonstrated the highest electron transfer activity, followed by FR (Table S5). Conversely, AS exhibited the lowest activity in all tests (Table S5). BL and RU showed low CUPRAC values, while RU also had low FRAP activity (Table S5). BL and RR recorded the lowest RP values (Table S5). Although the ranking of the extracts was consistent across assays, the absolute values varied significantly, particularly in terms of gallic acid equivalents (Table S5).

The FRAP assay yielded the highest gallic acid equivalent values, while CUPRAC and RP produced lower values. This discrepancy is attributed to the energetic conditions of each assay. FRAP, with its stronger oxidizing agent and acidic environment, is capable of quantifying both highly active antioxidants and those requiring more vigorous conditions to react. In contrast, CUPRAC and RP operate under milder conditions, primarily detecting compounds with inherently strong antioxidant properties, while leaving less reactive or degradation-dependent compounds unquantified.

The RP assay, despite involving mild heating, produced lower values than FRAP, reinforcing the influence of oxidant strength and assay sensitivity. These findings suggest that RP is particularly suited for evaluating antioxidants with high intrinsic activity that can react with weaker oxidizing agents.

#### 3.5.2. Metal Ion Chelation Assays

The metal-chelating capacity of the GP extracts was evaluated using two colorimetric assays: one for ferrous ions (Fe^2+^) and the other for cupric ions (Cu^2+^). In the ferrous ion assay, chelation was assessed based on the competition between the extract and ferrozine for Fe^2+^ binding. For cupric ion chelation, murexide was used as the chromogenic indicator. In both assays, the absorbance of the resulting solution is inversely proportional to the chelating activity of the extract, where a lower absorbance indicates a greater chelation, as fewer free metal ions remain to form complexes with the indicator dye.

The results of these assays are presented in Table S5. Among the tested samples, RB, JO, NE, FR, and RA exhibited the highest chelating activity toward ferrous ions, while AS and RU showed the lowest (Table S5). In the cupric ion chelation assay, the overall chelating capacity was higher than that observed for ferrous ions. The most effective extracts in chelating Cu^2+^ were RB and NE, whereas AS and RU again demonstrated the weakest activity (Table S5).

The antioxidant activities assessed by radical scavenging, reducing power, and metal-chelating assays (Table S5) further emphasized cultivar-specific differences. Neuburger (NE) and Fetească Regală (FR) exhibited the strongest DPPH and ABTS radical scavenging capacities, as well as superior FRAP, CUPRAC, and reducing power values, significantly surpassing weaker cultivars such as Astra (AS), Rubin (RU), and Amurg (AM) (*p* < 0.05). Among red cultivars, Regent (RE) demonstrated higher antioxidant capacity than Shiraz (SH) and Amurg (AM), consistent with its richer polyphenolic composition. The ferrous and cupric ion chelation activities were generally modest across cultivars, but significant differences were observed, with NE and TR showing higher values compared with AS and RU. Overall, antioxidant capacity did not strictly follow the red–white distinction: while anthocyanin-rich reds (RE, AM, SH) contributed to antioxidant activity, certain white cultivars (NE, FR, JO, TR) matched or even exceeded them, underlining the central role of flavanols and hydroxybenzoic acids in determining antioxidant potential (Figure 7).

These findings indicate that certain GP extracts, particularly RB and NE, possess strong metal-chelating properties, which may contribute to their overall antioxidant potential by limiting the availability of transition metals that catalyze free radical formation.

**Figure 8:**
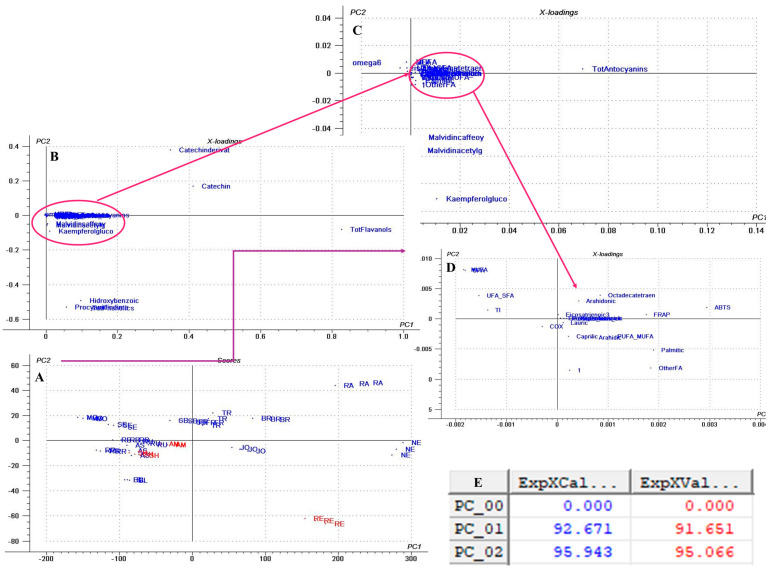
Score plot of the first two principal components (PC1 and PC2) (**A**) based on the phenolic compounds (see Table S2), fatty acid profiles and their derived calculated indices (see Table S4), and antioxidant activities (see Table S5) in 17 grape pomace extracts; (**B**) loading plot of PC1 and PC2 showing how the analyzed variables contributes to grape pomace samples distribution among the principal components; (**C**) zoom 1 of loading variables in the loading score plot; (**D**) zoom 2 of loading variables in the loading score plot to differentiate the superposed variables; (**E**) explained variance of PCA model. The analyzed samples included 14 white grape cultivars—Blasius (BL), Rhine Riesling (RR), Roze Blaj (RB), Astra (AS), Traminer roz (TR), Johaniter (JO), Neuburger (NE), Rubin (RU), Sauvignon Blanc (SB), Feteascǎ Regalǎ (FR), Radames (RA), Brumăriu (BR), Selena (SE), and Muscat Ottonel (MO)—and three red grape cultivars—Regent (RE), Syrah (SH), and Amurg (AM).

**Figure 9:**
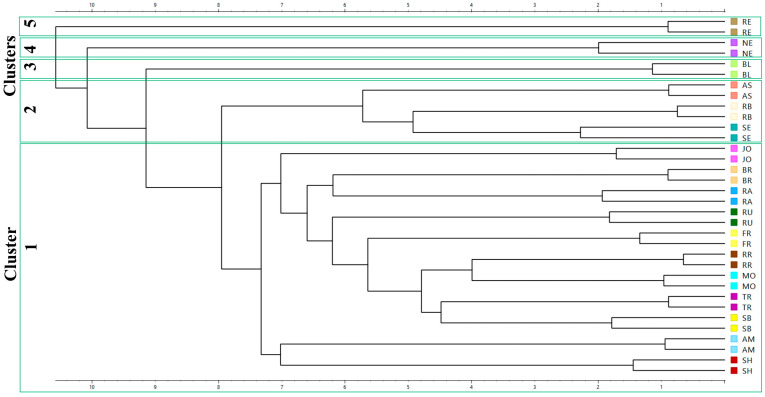
Hierarchical clustering dendrogram (Euclidean distance, average linkage as the clustering method) based on the phenolic compounds (Tables S2 and S3), fatty acid profiles and their derived calculated indices (Table S4), and antioxidant activities (Table S5) in 17 grape pomace extracts. Each row represents the investigated parameter, while each row represents the white grape cultivars analyzed, including Blasius (BL), Rhine Riesling (RR), Roze Blaj (RB), Astra (AS), Traminer roz (TR), Johaniter (JO), Neuburger (NE), Rubin (RU), Sauvignon Blanc (SB), Feteascǎ Regalǎ (FR), Radames (RA), Brumăriu (BR), Selena (SE), and Muscat Ottonel (MO). The red grape cultivars included Regent (RE), Syrah (SH), and Amurg (AM).

**Figure 10:**
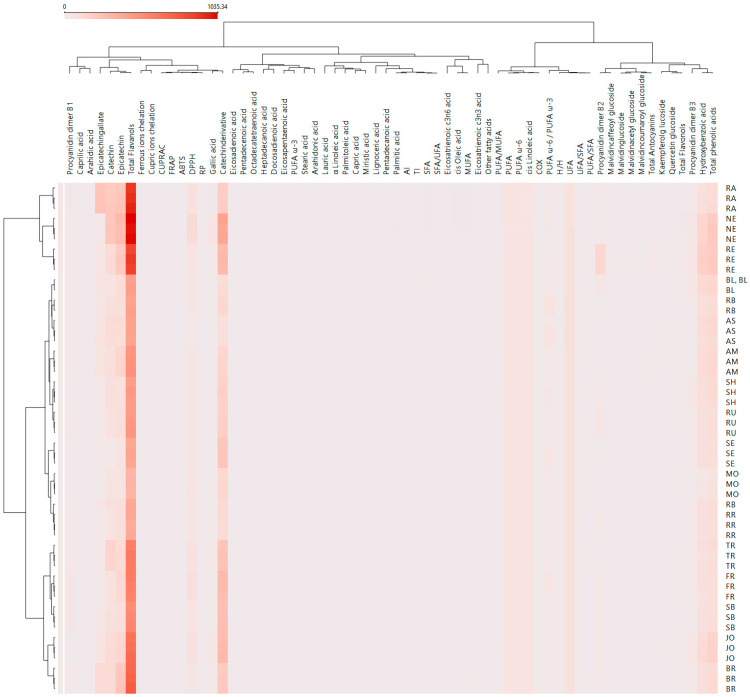
Heat map analysis based on the phenolic compounds (see table), fatty acid profiles and their derived calculated indices (see table), and antioxidant activities (see table) in 17 grape pomace extracts. Each column represents the investigated parameter, while each row represents the white grape cultivars analyzed, including Blasius (BL), Rhine Riesling (RR), Roze Blaj (RB), Astra (AS), Traminer roz (TR), Johaniter (JO), Neuburger (NE), Rubin (RU), Sauvignon Blanc (SB), Feteascǎ Regalǎ (FR), Radames (RA), Brumăriu (BR), Selena (SE), and Muscat Ottonel (MO). The red grape cultivars included Regent (RE), Syrah (SH), and Amurg (AM).

The multivariate analysis PCA (Figure 8), the hierarchical clustering dendrogram (Figure 9), and the heat map (Figure 10) of all the parameters determined in GP extracts characterization were further used in sample grouping to identify which variables were influencing the sample clustering. The first two, PC1 and PC2, explained 94.11% of the total variance, demonstrating the data set’s discriminative power. The corresponding loading plots (Figure 8B–D) identified total flavanols, catechin derivative, epicatechin, and catechin as major compounds influencing the sample clustering among the two axes.

Hierarchical cluster analysis completed the previous PCA analysis, revealed that clusters (5) RE, (4) NE, and (3) BL remained distinct, separating from the other samples at an early stage of clustering, suggesting a unique profile compared to all other groups (Figure 9). Small cluster 2 (AS, RB, and SE) grouped the cultivars, which are characterized by high fatty acid composition (Figure 5). Finally, the biggest cluster (1) grouped the average samples (Figure 9).

Heatmap visualization (Figure 10) provided a complementary overview of the relationships among GP cultivars and the analyzed parameters. The clustering patterns aligned with those observed in PCA and hierarchical clustering.

The Spearman correlation analysis revealed multiple significant associations between the fatty acid composition, antioxidant activities, and phenolic profiles of the 17 grape pomace extracts. Considering the correlation coefficient (R Spearman), a strong positive correlation was observed between hydroxybenzoic acid and total phenolic acids (R= 0.978, *p* < 0.01). Flavanol compounds also showed important relationships: catechin derivatives correlated positively with docosadienoic acid (R = 0.465, *p* < 0.01), suggesting a potential co-occurrence between these flavanols and specific polyunsaturated fatty acids. Moreover, catechin, epicatechin, and procyanidin oligomers exhibited multiple significant intercorrelations as well as positive associations with antioxidant activity assays, confirming the role of flavanols as key determinants of antioxidant potential. Regarding fatty acids, arachidonic acid displayed consistent negative associations with lipid quality and oxidative indices. Significant negative correlations were found with the UFA/SFA ratio (R = –0.482, *p* < 0.01), the PUFA/SFA ratio (R = –0.447, *p* < 0.01), and the COX index (R = –0.561, *p* < 0.01). In addition, the PUFA ω-6/PUFA ω-3 ratio showed negative correlations with hydroxybenzoic acid (R = –0.352, *p* < 0.05), total flavanols (R = –0.621, *p* < 0.01), and total phenolic acids (R = –0.434, *p* < 0.05), suggesting that higher phenolic content is associated with a more favorable PUFA ω-6/PUFA ω-3 lipid balance. The ratio was also inversely associated with several individual fatty acids (e.g., arachidonic, octadecatetraenoic, eicosadienoic, and stearic acids) and MUFA content, while showing a positive correlation with total PUFA (R = 0.613, *p* < 0.01). Lipid quality indices further correlated with antioxidant activities. Both the atherogenic index (AI) and thrombogenic index (TI) were positively correlated with ABTS, FRAP, and DPPH assays (R = 0.37–0.55, *p* < 0.05–0.01), indicating that extracts with higher atherogenic and thrombogenic potential also exhibited stronger antioxidant activity. Conversely, the hypocholesterolemic/hypercholesterolemic ratio (H/H) was negatively correlated with all three assays (R = –0.36 to –0.51, *p* < 0.05–0.01), suggesting that extracts with a more favorable lipid balance displayed lower antioxidant responses. No significant correlations were observed between the COX index and antioxidant activities. Overall, the correlation analysis indicates that the antioxidant capacity of the extracts is strongly related to the phenolic composition, particularly hydroxybenzoic acid and flavanols, while the fatty acid profile, especially arachidonic acid and the PUFA ω-6/PUFA ω-3 ratio, exerts an opposing influence on lipid quality indices. Moreover, indices of lipid nutritional quality (AI, TI, H/H) were also significantly linked to antioxidant responses, highlighting complex interactions between phenolics, fatty acids, and antioxidant potential in the extracts.

The antioxidant capacity of the studied GPs, as determined by their phenolic composition, is consistent with previous research, highlighting the well-established antioxidant properties of phenolic compounds [127]. Antioxidant activity plays a crucial role in mitigating pathological alterations induced by oxidative stress by neutralizing reactive oxygen species. Oxidative stress occurs when there is an imbalance between oxidants and antioxidants, resulting in excessive free radical production that can damage essential biomolecules. Lipids undergo peroxidation, proteins experience peptide chain fragmentation and alterations in electrical charge, and DNA suffers degradation of purine and pyrimidine bases, resulting in mutations, translocations, or deletions [128]. These molecular disruptions are implicated in the pathogenesis of chronic diseases, including atherosclerosis, diabetes, and cancer [76]. Moreover, oxidative stress is known to trigger inflammatory responses that further exacerbate cellular and tissue damage. Previous studies have demonstrated that phenolic compounds exert protective effects by reducing LDL oxidation, suppressing inflammation, and inhibiting platelet activation, all mechanisms that collectively contribute to slowing the progression of atherosclerosis. Additionally, several investigations have reported that grape polyphenols exert beneficial effects on metabolic syndrome, a significant risk factor for numerous chronic conditions [129,130,131,132].

Ramos-Romero et al. (2021) reported significant inter-individual variability in insulin response following GP supplementation in subjects at high cardiometabolic risk, highlighting the potential role of gut microbiota and microRNAs in mediating these effects [133]. Furthermore, GP polyphenols have been associated with reductions in cardiovascular disease risk factors, including trimethylamine N-oxide levels, as demonstrated in a randomized, placebo-controlled, crossover study using a polyphenolic extract (Taurisolo^®^) [134]. Additional evidence supports their beneficial impact on hypertension and hyperglycemia, as shown in studies evaluating grape-pomace-derived seasonings in both high-cardiovascular-risk and healthy individuals, with observed interactions involving the gut microbiome [135].

**Figure 11:**
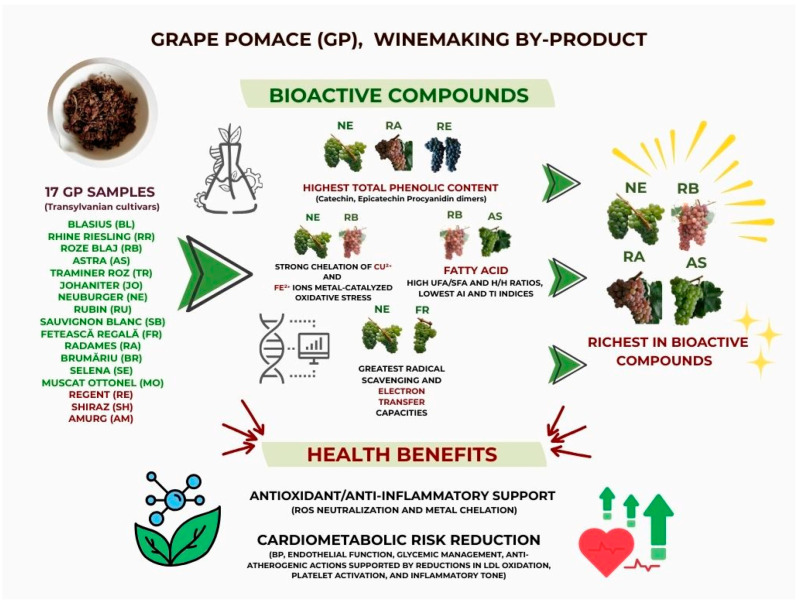
Bioactive profiles and cardioprotective benefits of the most promising studied grape pomaces (GPs)—premises for their sustainable valorization.

In this context, the GPs analyzed in this present study represent a reliable source of polyphenols that could serve as adjuvant therapies in managing pathologies characterized by oxidative stress and inflammatory processes [76] (Figure 11).

## 4. Conclusions

This study provides a comprehensive comparison of 17 GP extracts from cultivars grown in Transylvania, demonstrating their phenolic composition, fatty acid profiles, and antioxidant potential. The findings highlight the significant variability that exists among cultivars, with specific cultivars exhibiting particularly promising profiles in the context of human health.

Among the red and white grape varieties analyzed, cultivars such as NE, RA, and RE, in particular, contained high concentrations of total phenolic compounds, particularly flavan-3-ols, including catechin, epicatechin, and procyanidin dimers. In particular, NE exhibited the highest total flavanol content and antioxidant activity across all the assays that were performed, which included DPPH˙, ABTS˙^+^, FRAP, CUPRAC, and RP. FR also demonstrated strong antioxidant activity. However, cultivars such as SE and MO exhibited the lowest concentrations of phenolics and antioxidant activity.

The analysis of fatty acids revealed that RB and AS cultivars possessed the most favorable nutritional profiles, characterized by high UFA/SFA and H/H ratios and the lowest AI and TI indices. The high UFA/SFA and H/H ratios, along with low AI and TI indices, indicate that these cultivars offer potential benefits for cardiovascular health. Furthermore, RB and NE demonstrated strong metal-chelating activity with regards to Cu^2+^ and Fe^2+^ ions in particular, which could further enhance their antioxidant efficacy by alleviating the occurrence of metal-catalyzed oxidative stress.

Overall, the findings provide evidence to suggest that GP, particularly from cultivars such as NE, RB, RA, and AS, are valuable sources of bioactive compounds featuring antioxidant and cardioprotective properties. Notably, RB, RA, and AS are homologated cultivars in Transylvania at SCDVV Blaj, underscoring their regional significance and potential for local valorization. Therefore, these findings contribute to an increasing body of evidence supporting the sustainable valorization of winemaking by-products that can be utilized in food, nutraceuticals, and other applications to achieve improved human health.

This study has several limitations. First, all samples were collected in a single year (2019), which precludes assessment of interannual climatic variability and its potential influence on metabolite profiles, thereby limiting the generalizability of the findings. Second, the analysis was restricted to GP from Transylvanian cultivars. Although 17 types were included—those available from the SCDVV Blaj research center—this selection may not represent the full diversity of cultivars used in winemaking within the entire region. Third, oxidation markers of GP were not monitored, the dry-weight normalization was not performed for all the results, and practical considerations related to scaling up valorization processes for industrial applications were not presented in this work.

Future research should address these gaps by evaluating year-to-year variability in GP bioactive compound content under different climatic conditions, monitoring oxidation parameters during storage, and exploring strategies for extract standardization to enable industrial-scale valorization in the food and nutraceutical sectors. Additionally, in vivo studies using animal models should be conducted to investigate the cardioprotective potential of GP extracts in conditions such as induced myocardial infarction, hypertension, and dyslipidemia, with a focus on anti-inflammatory and antioxidant effects.

## Figures and Tables

**Table 1:** Characteristics of the grapevine cultivars taken into study [34].

Cultivar	Skin Color	Usage	Genetic Origin	
Blasius (BL), created and homologated at SCDVV Blaj, 1994	White	Grapevine cultivar for white wine	*Vitis vinifera* ssp. sativa L. (Traminer roz x Iordană) (Raisin de Saint Piere x Perlă de Csaba)	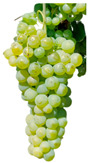
Riesling de Rhin (RR)	White	Grapevine cultivar for white wine	*Vitis vinifera* ssp. sativa H. ? x Heunisch Weiss	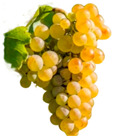
Roze Blaj (RB), created and homologated at SCDVV Blaj, 2020	Rosé	Grapevine cultivar for white wine	Sexuate intercrossing of two elites 8-33-44 (Iordană x Traminer roz) x 51-19 (Raisin de Saint Pierre x Perla de Csaba).	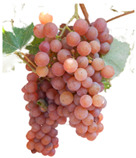
Astra (AS), created and homologated at SCDVV Blaj, 1995	White	Grapevine cultivar for white wine	*Vitis vinifera* ssp. sativa L.; Fetească regală x Pinot gris	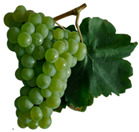
Traminer roz (TR)	Rosé	Grapevine cultivar for white wine	*Vitis vinifera* ssp. sativa H. Sauvignon Blanc mutation	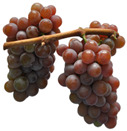
Johanniter (JO)	White	Grapevine cultivar for white wine	*Vitis vinifera* ssp. sativa H. Riesling weiss x Freiburg 589-54	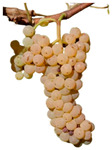
Neuburger (NE)	White	Grapevine cultivar for white wine	*Vitis vinifera* ssp. sativa H. Veltliner Rot x Sylvaner	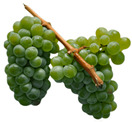
Rubin (RU), created and homologated at SCDVV Blaj, 2007	Rosé	Grapevine cultivar for white wine	Sexuate interspecific hybridization between the Traminer roz cultivar and a hybrid descendant (Seyve Villard 12375 x Regina viilor)	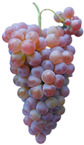
Sauvignon Blanc (SB)	White	Grapevine cultivar for white wine	*Vitis vinifera* ssp. sativa H. Savagnin blanc x Traminer x ?	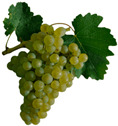
Fetească regală (FR)	White	Grapevine cultivar for white wine	*Vitis vinifera* ssp. sativa H. Fetească albă x Frâncușe	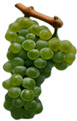
Radames (RA), created and homologated at SCDVV Blaj, 1993	Rosé	Grapevine cultivar for white wine	Interspecific hybrid Traminer roz x Seyve Villard 12.375	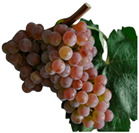
Brumăriu (BR), created and homologated at SCDVV Blaj, 1983	White	Grapevine cultivar for white wine	Interspecific hybrid Saint Emilion x Rayon d’Or	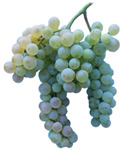
Selena (SE), created and homologated at SCDVV Blaj, 1995	Rosé	Grapevine cultivar for white wine	*Vitis vinifera* ssp. sativa L. Sexuate hybridization between Iordană cultivars x Traminer roz	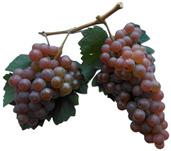
Muscat Ottonel (MO)	White	Grapevine cultivar for white wine	*Vitis vinifera* ssp. sativa H. Ingram’s Muscat x Chasselas blanc	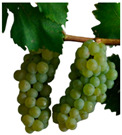
Regent (RE)	Dark red	Grapevine cultivar for red wine	*Vitis vinifera* ssp. sativa H. Diana x Chambourcin	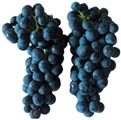
Syrah (SH)	Dark red	Grapevine cultivar for red wine	*Vitis vinifera* ssp. sativa H. Mondeuse blanche x Dureza	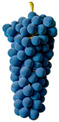
Amurg (AM), created and homologated at SCDVV Blaj, 1989	Dark red	Grapevine cultivar for red wine	*Vitis vinifera* ssp. sativa L. Muscat de Hamburg x Cabernet Sauvignon	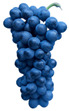

**Table 2:** Total polyphenol content (TPC) for the 17 grape pomace (GP) extracts analyzed using the Folin–Ciocalteu method.

GP Sample	TPC ^1^ (mgGAE/g GP)
BL	46.38 ± 0.40 ^b^
RR	52.07 ± 0.83 ^c^
RB	53.82 ± 1.03 ^c,d^
AS	42.97 ± 0.20 ^a,b^
TR	79.24 ± 2.37 ^h^
BR	51.46 ± 0.43 ^c^
JO	77.59 ± 1.54 ^h^
SE	56.43 ± 0.18 ^d,e^
NE	76.72 ± 0.79 ^h^
MO	54.11 ± 0.22 ^c,d^
RU	42.38 ± 0.08 ^a^
RA	64.95 ± 0.43 ^f^
SB	56.85 ± 0.62 ^d,e^
FR	72.56 ± 1.23 ^g^
AM	51.14 ± 0.35 ^c^
SH	59.71 ± 3.19 ^e^
RE	72.05 ± 0.91 ^g^

^1^ Values are presented as means of triplicate measurements ± standard deviation and are expressed in mg/g of GP. Statistical analysis was performed using a one-way ANOVA followed by Tukey’s post hoc analysis. Within each row, different letters indicate statistically significant differences (*p* < 0.05). Total phenolic content (TPC) is expressed as milligrams of gallic acid equivalents per gram of GP (mg GAE/g GP). The white grape cultivars analyzed included Blasius (BL), Rhine Riesling (RR), Roze Blaj (RB), Astra (AS), Traminer roz (TR), Johaniter (JO), Neuburger (NE), Rubin (RU), Sauvignon Blanc (SB), Feteascǎ Regalǎ (FR), Radames (RA), Brumăriu (BR), Selena (SE), and Muscat Ottonel (MO). The red grape cultivars included Regent (RE), Syrah (SH), and Amurg (AM).

## Data Availability

The original contributions presented in this study are included in the article. Further inquiries can be directed at the corresponding authors.

## Supplementary Materials

The following are available online at https://www.mdpi.com/article/10.3390/antiox14101152/s1, Figure S1. Calibration curves for the quantitative tests performed in this study, Figure S2. Chromatograms of phenolic extracts at 280 nm, 340 nm and 520 nm (for red GPs), Figure S3. UV-Vis DAD and MS after positive ionization spectra of the identified phenolic compounds, Table S1. Limit of detection (LOD), limit of quantification (LOQ) and recovery percentage of analyzed phenolics and fatty acids, Table S2. Flavanol (μg/mL catechin equivalents) and hydroxybenzoic acid (μg/mL gallic acid equivalents) compounds determined in the grape pomace extracts by LC-MS, Table S3. Flavonols (μg/mL rutin equivalents) and anthocyanins (μg/mL cyanidin equivalents) determined in the grape pomace extracts by LC-MS, Table S4. Fatty acids composition (g/100 g of dried sample) of the studied grape pomaces by GC-FID, Table S5. Antiradical activity of the samples evaluated using the capacity of scavenging DPPH˙ and ABTS˙^+^, the electron donation capacity using the FRAP, RP and CUPRAC methods and the ferrous and cupric ions chelation capacity

## Abbreviations

The following abbreviations are used in this manuscript:

GP	grape pomace
BL	GP from Blasius grapes
RR	GP from Rhine Riesling grapes
RB	GP from Roze Blaj grapes
AS	GP from Astra grapes
TR	GP from Traminer roz grapes
JO	GP from Johaniter grapes
NE	GP from Neuburger grapes
RU	GP from Rubin grapes
SB	GP from Sauvignon Blanc grapes
FR	GP from Fetească Regală grapes
RA	GP from Radames grapes
BR	GP from Brumăriu grapes
SE	GP from Selena grapes
MO	GP from Muscat Ottonel grapes
RE	GP from Regent grapes
SH	GP from Syrah grapes
AM	GP from Amurg grapes
FTIR	Fourier transform infrared spectroscopy
HPLC-DAD-ESI MS	liquid chromatography–diode array detection–electrospray ionization mass spectrometry
GC-FID	gas chromatography with flame ionization detector
DPPH	2,2-diphenyl-1-picrylhydrazyl radical-scavenging capacity
ABTS	2,2′-azinobis-(3-ethylbenzthiazolin-6-sulfonic acid) radical-scavenging capacity
CUPRAC	Cupric-reducing antioxidant capacity
FRAP	Ferric-reducing antioxidant potential
RP	reducing power
TI	thrombogenicity index
AI	atherogenicity index
H/H	ratio between hypo and hypercholesterolemic fatty acids
COX	calculated oxidizability
TPC	total polyphenol content
GAE	gallic acid equivalent
TE	Trolox equivalent
EE	EDTA equivalent
SFA	saturated fatty acid
UFA	unsaturated fatty acid
MUFA	monounsaturated fatty acid
PUFA	polyunsaturated fatty acid
