# Supplementary material for: Transylvanian Grape Pomaces as Sustainable Sources of Antioxidant Phenolics and Fatty Acids—A Study of White and Red Cultivars
Source: Antioxidants (Basel). 2025 Sep 23;14(10):1152. doi: 10.3390/antiox14101152 (PMC12562140; doi:10.3390/antiox14101152)
Supplement: Supplementary file 1 [file antioxidants-14-01152-s001.zip › antioxidants-3814191-supplementary.pdf]

Supplementary Material

Transylvanian Grape Pomaces as Sustainable Sources of Antioxidant Phenolics and Fatty Acids - A Study of White and Red Cultivars

Veronica Sanda Chedea[1] *, Liliana Lucia Tomoiagă[1], Mariana Ropota[2], Gabriel Marc[3], Floricuta Ranga[4], Maria Doinița Muntean[1], Alexandra Doina Sîrbu[1], Ioana Sorina Giurca[1], Maria Comșa[1], Ioana Corina Bocsan[5], Anca Dana Buzoianu[5], Hesham Kisher[6], Raluca Maria Pop [5]*

[1]Research Station for Viticulture and Enology Blaj (SCDVV Blaj), Gheorhe Barițiu Street, No 2, Blaj, Romania; chedeaveronica@yahoo.com (V.S.C), tomoiagaliliana@yahoo.com (L.L.T), maria.doinita@gmail.com (M.D.M), sirbu.alexandra@ymail.com (A.D.S), tirnovean.ioana@gmail.com (I.S.G), comsa_m@yahoo.com (M.C),
[2]Laboratory of Chemistry and Nutrition Physiology, National Research Development Institute for Animal Biology and Nutrition (IBNA Balotesti), Balotesti, Ilfov, Romania; m.ropota@yahoo.com (M.R)
[3]Department of Organic Chemistry, "Iuliu Hațieganu" University of Medicine and Pharmacy, 41 Victor Babeș Street, RO-400012 Cluj-Napoca, Romania; marc.gabriel@umfcluj.ro (G.M)
[4]Food Science and Technology, Department of Food Science, University of Agricultural Science and Veterinary Medicine Cluj-Napoca, Calea Mănăștur, No 3-5, 400372, Cluj-Napoca România; flori-cutza_ro@yahoo.com (F.R)
[5]Pharmacology, Toxicology and Clinical Pharmacology, Department of Morphofunctional Sciences, "Iuliu Hațieganu" University of Medicine and Pharmacy, Victor Babeș, No 8, 400012, Cluj-Napoca, Romania, raluca_parlog@yahoo.com (R.M..P), bocsan.corina@umfcluj.ro (I.C.B), abuzoianu@**umfcluj**.ro (A.D.B)
[6]School of Applied Sciences, University of the West of England, Bristol BS16 1QY, UK; hesham.kisher@uwe.ac.uk (H.C)
*Correspondence: chedeaveronica@yahoo.com (V.S.C); raluca_parlog@yahoo.com (R.M.P)

The GPs studied in this work come from Târnave vineyard. The vineyard's name derives from the location of the vineyards along the slopes bordering the valleys of the Târnave Mare and Târnava Mică rivers [28,30,31]. Renowned for producing high-quality wines with distinctive aromatic profiles and a well-balanced sugar-to-acidity ratio [28,29,31], the Târnave region offers favorable environmental conditions and a suitable growing season for grapevine cultivation.

Viticulture in Transylvania has deep historical roots, dating back to antiquity and evolving through successive cultural and agricultural developments, particularly during the feudal period [28,30]. The enduring interest of local communities in grape cultivation and winemaking reflects the cultural and economic significance of this region [28].

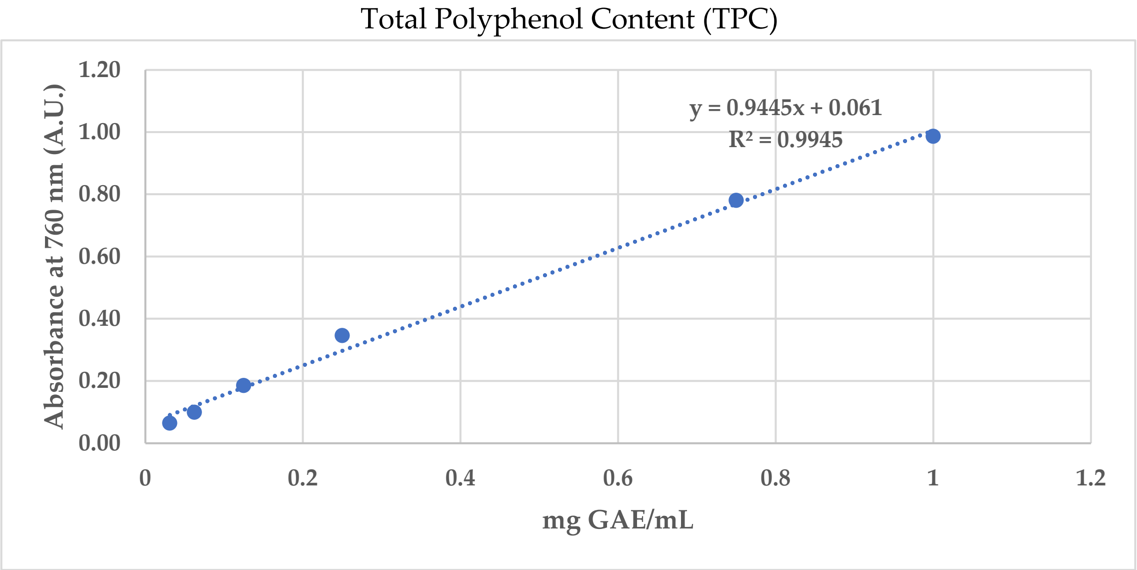

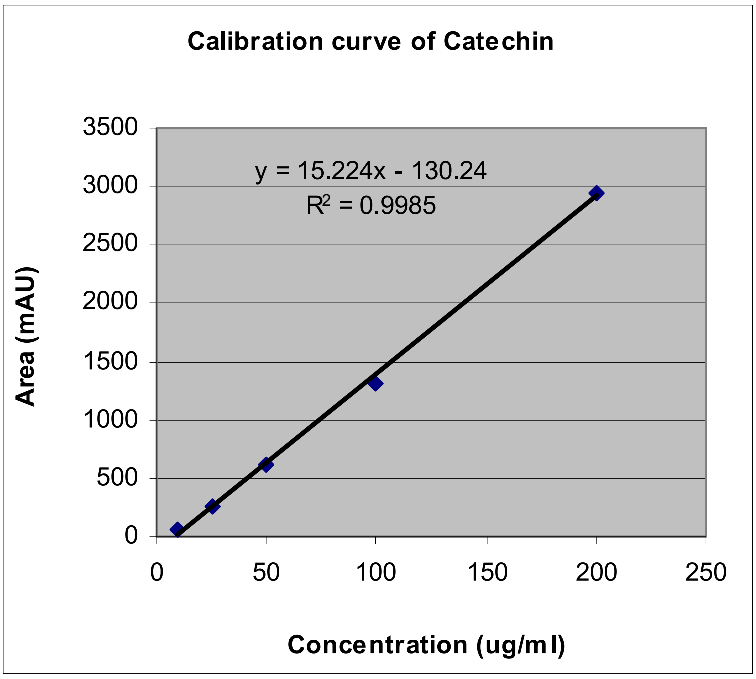
Calibration curve of Catechin
y = 15.224x - 130.24
R2 = 0.9985
Area (mAU)
Concentration (ug/ml)
0
500
1000
1500
2000
2500
3000
3500
0
50
100
150
200
250

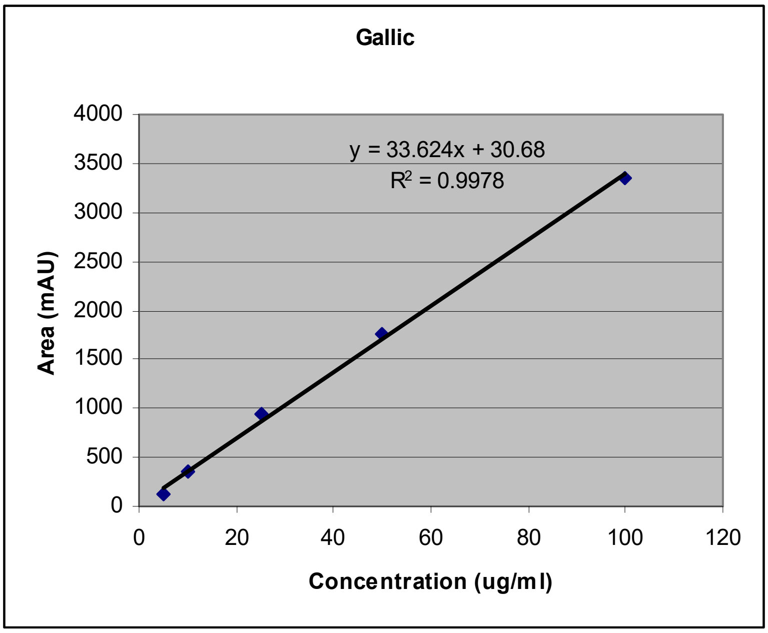
Gallic
y = 33.624x + 30.68
R2 = 0.9978
Area (mAU)
Concentration (ug/ml)
0
500
1000
1500
2000
2500
3000
3500
4000
0
20
40
60
80
100
120

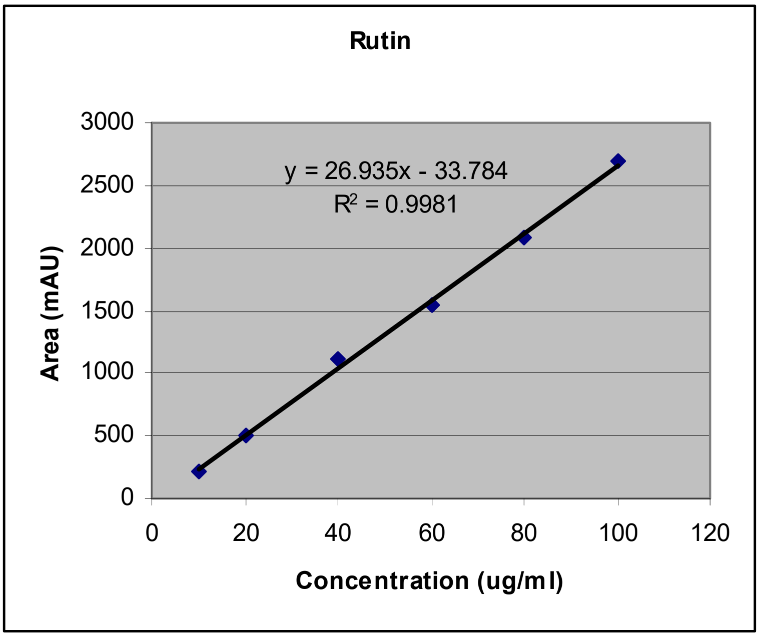
Rutin
y = 26.935x - 33.784
R2 = 0.9981
Area (mAU)
Concentration (ug/ml)
0
500
1000
1500
2000
2500
3000
0
20
40
60
80
100
120

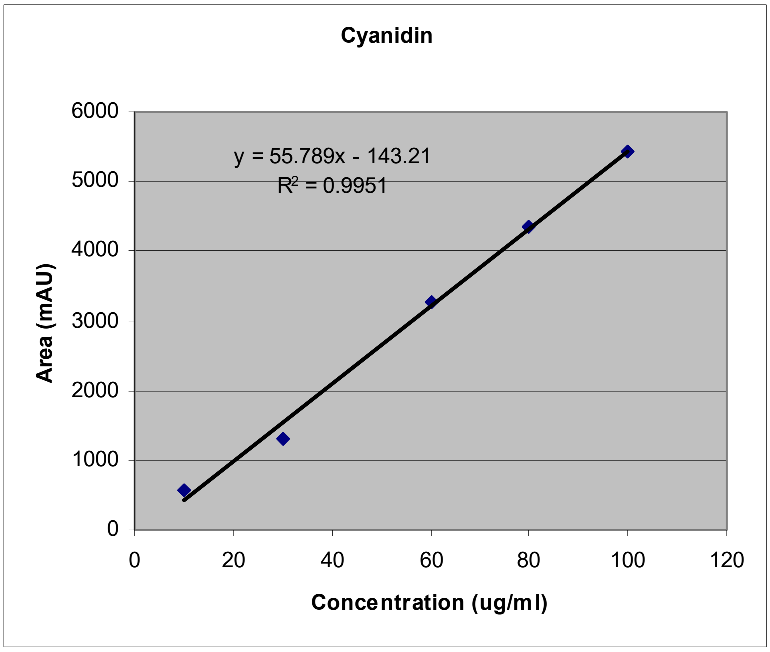
Cyanidin
y = 55.789x - 143.21
R² = 0.9951
Area (mAU)
Concentration (ug/ml)

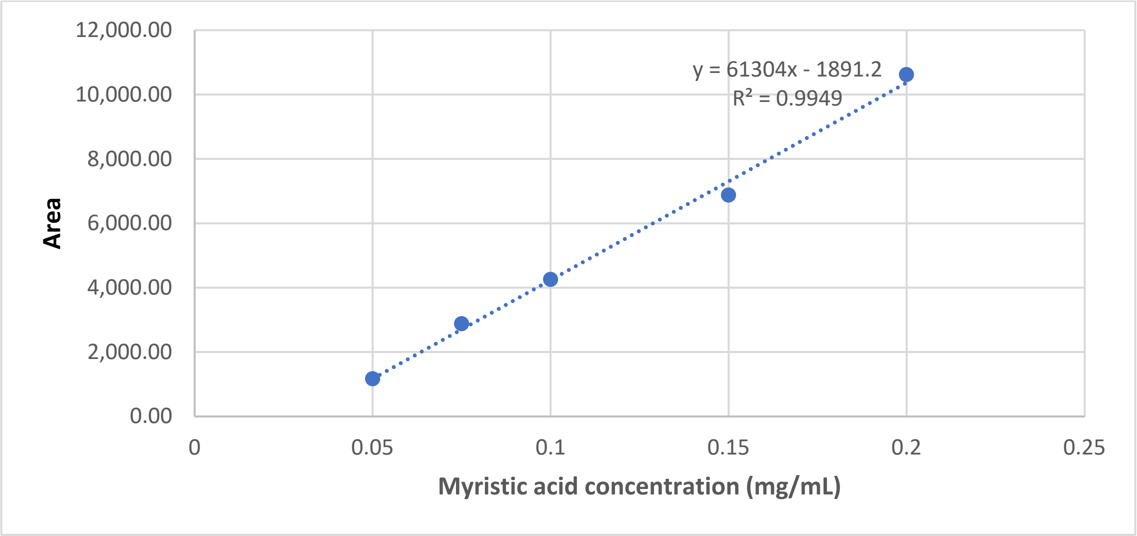
y = 61304x - 1891.2
R² = 0.9949
Area
Myristic acid concentration (mg/mL)

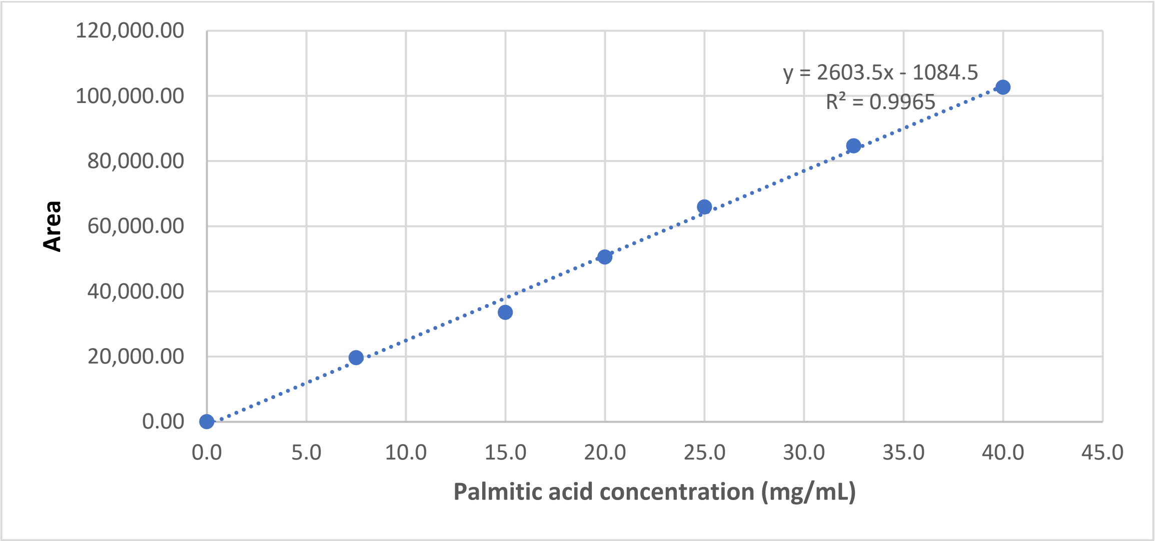
y = 2603.5x - 1084.5
R² = 0.9965
Area
Palmitic acid concentration (mg/mL)

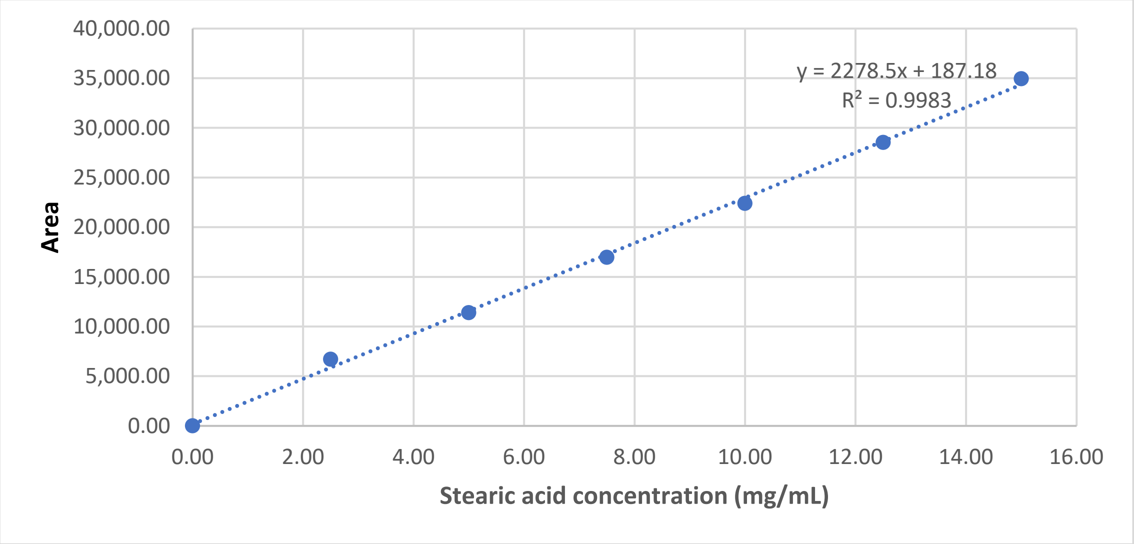
y = 2278.5x + 187.18
R² = 0.9983
Area
Stearic acid concentration (mg/mL)
0.00
5,000.00
10,000.00
15,000.00
20,000.00
25,000.00
30,000.00
35,000.00
40,000.00
2.00
4.00
6.00
8.00
10.00
12.00
14.00
16.00

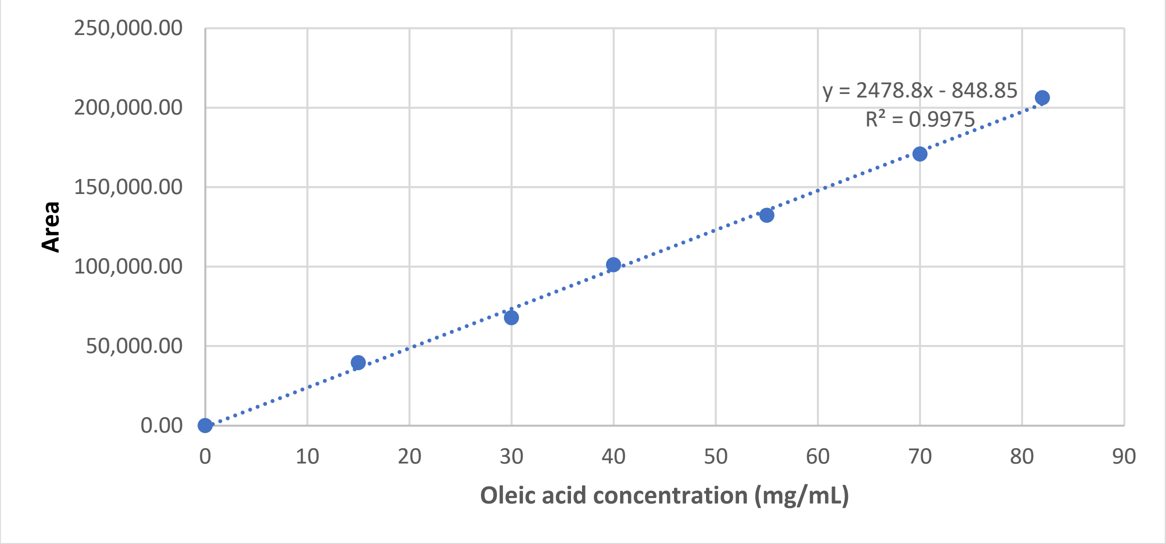
y = 2478.8x - 848.85
R² = 0.9975
Area
Oleic acid concentration (mg/mL)
0.00
50,000.00
100,000.00
150,000.00
200,000.00
250,000.00
0
10
20
30
40
50
60
70
80
90

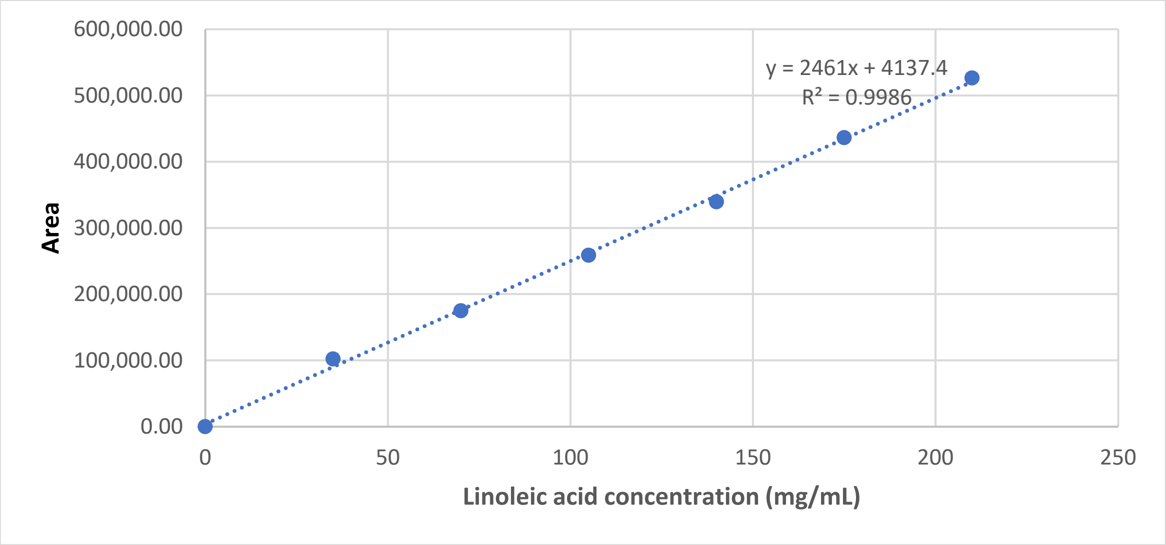
y = 2461x + 4137.4
R² = 0.9986
Area
Linoleic acid concentration (mg/mL)
0.00
100,000.00
200,000.00
300,000.00
400,000.00
500,000.00
600,000.00
0
50
100
150
200
250

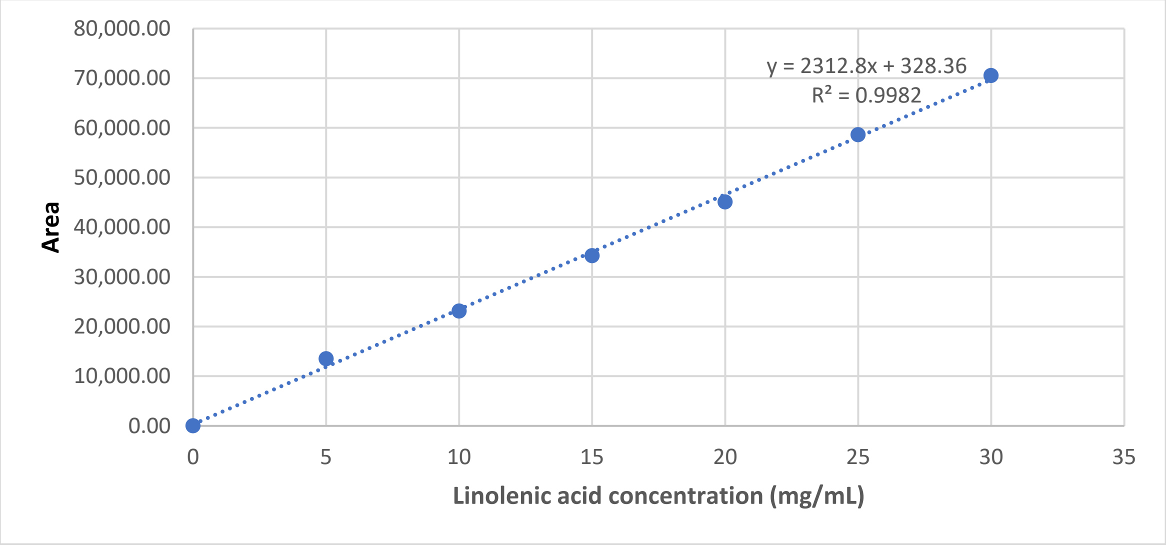

2,2-diphenyl-1-picrylhydrazyl radical-scavenging capacity (DPPH)

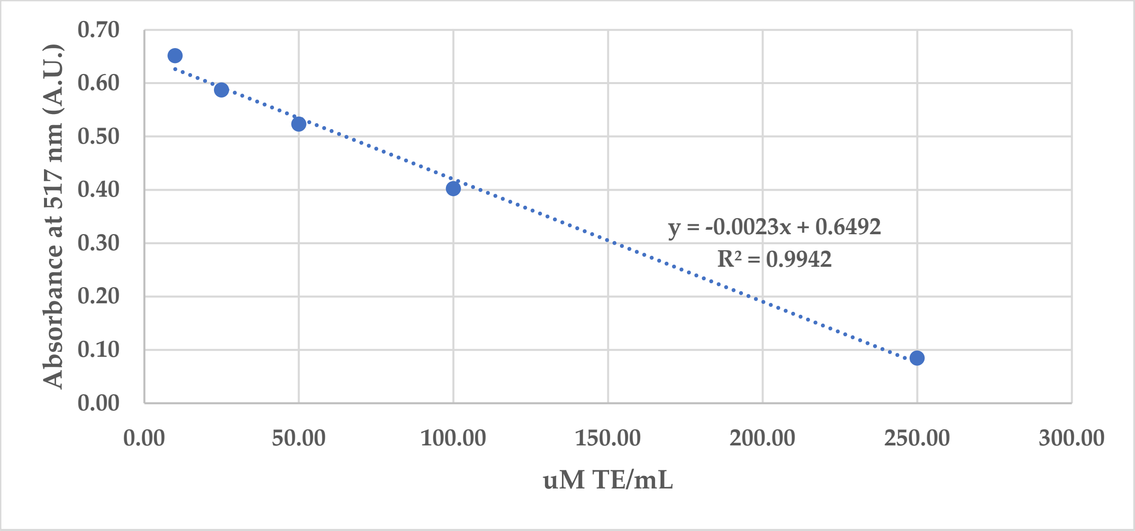

2,2′-azinobis-(3-ethylbenzthiazolin-6-sulfonic acid) radical-scavenging capacity (ABTS)

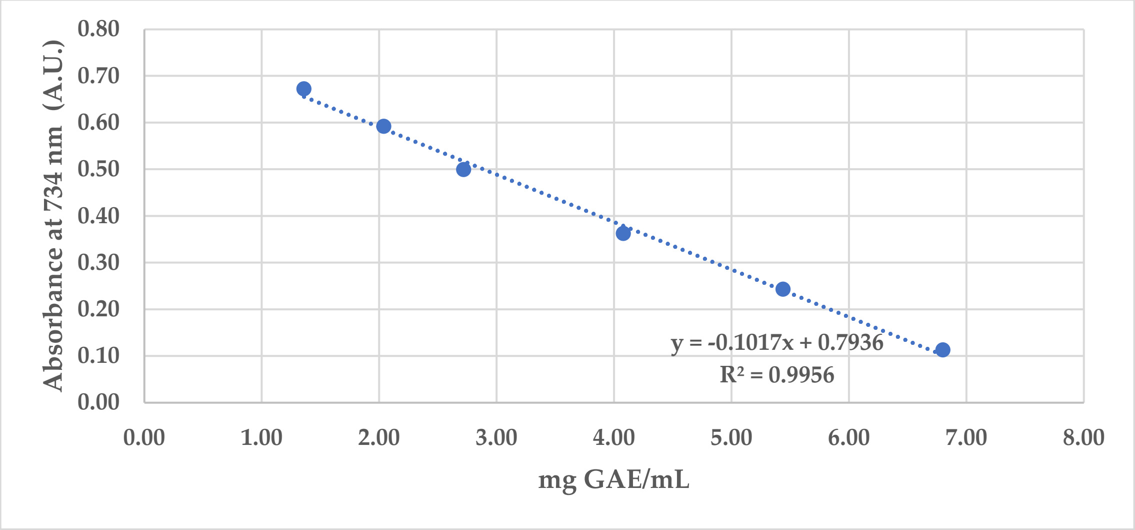

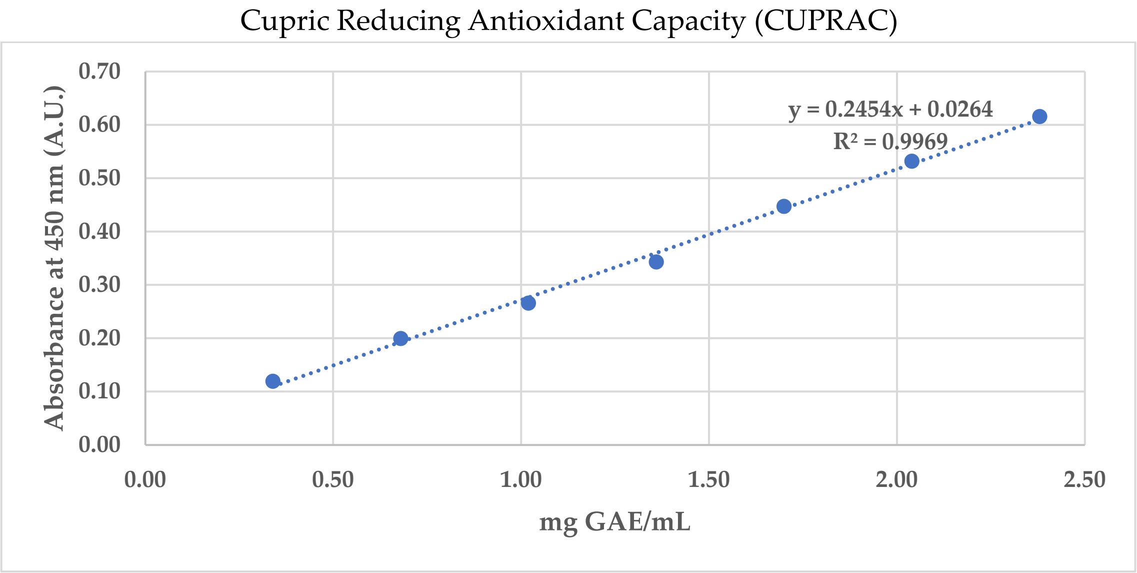
Cupric Reducing Antioxidant Capacity (CUPRAC)
Absorbance at 450 nm (A.U.)
0.70
0.60
0.50
0.40
0.30
0.20
0.10
0.00
0.00
0.50
1.00
1.50
2.00
2.50
mg GAE/mL
y = 0.2454x + 0.0264
R² = 0.9969

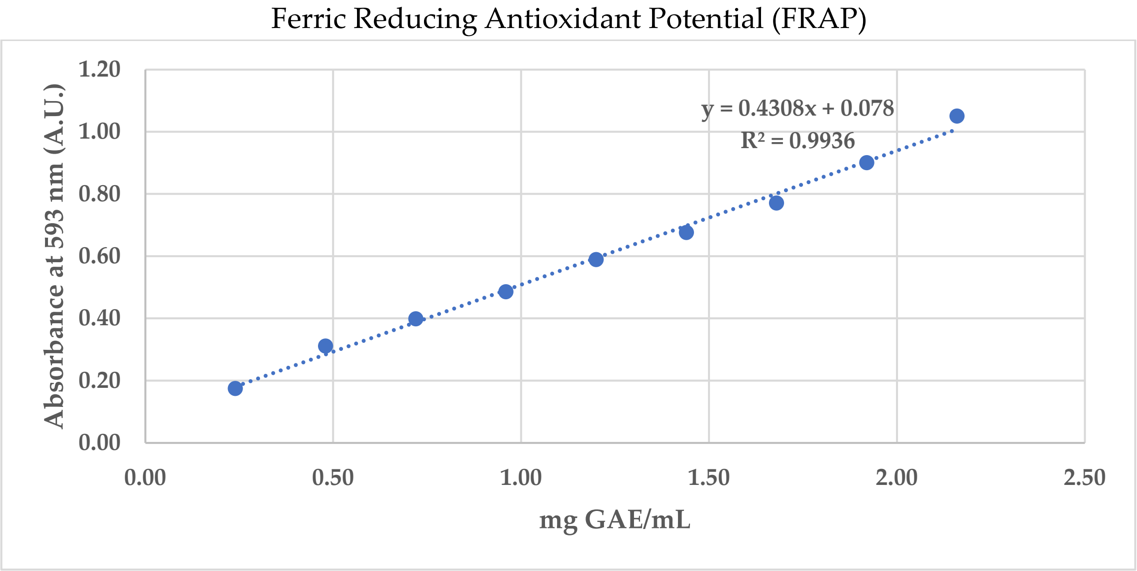
Ferric Reducing Antioxidant Potential (FRAP)
Absorbance at 593 nm (A.U.)
1.20
1.00
0.80
0.60
0.40
0.20
0.00
0.00
0.50
1.00
1.50
2.00
2.50
mg GAE/mL
y = 0.4308x + 0.078
R² = 0.9936

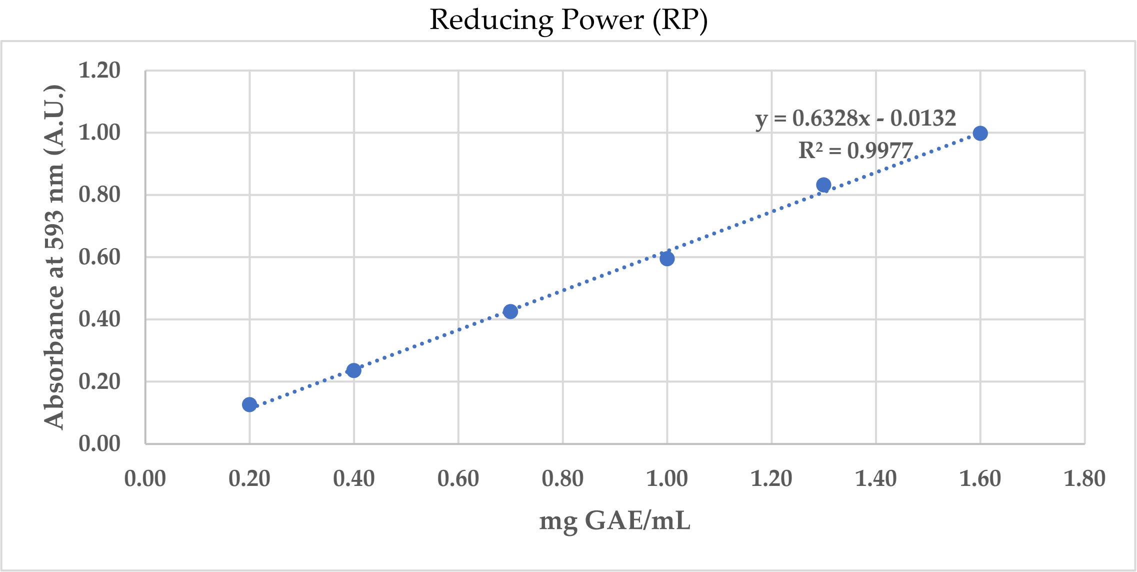
Reducing Power (RP)
Absorbance at 593 nm (A.U.)
1.20
1.00
0.80
0.60
0.40
0.20
0.00
0.00
0.20
0.40
0.60
0.80
1.00
1.20
1.40
1.60
1.80
mg GAE/mL
y = 0.6328x - 0.0132
R² = 0.9977

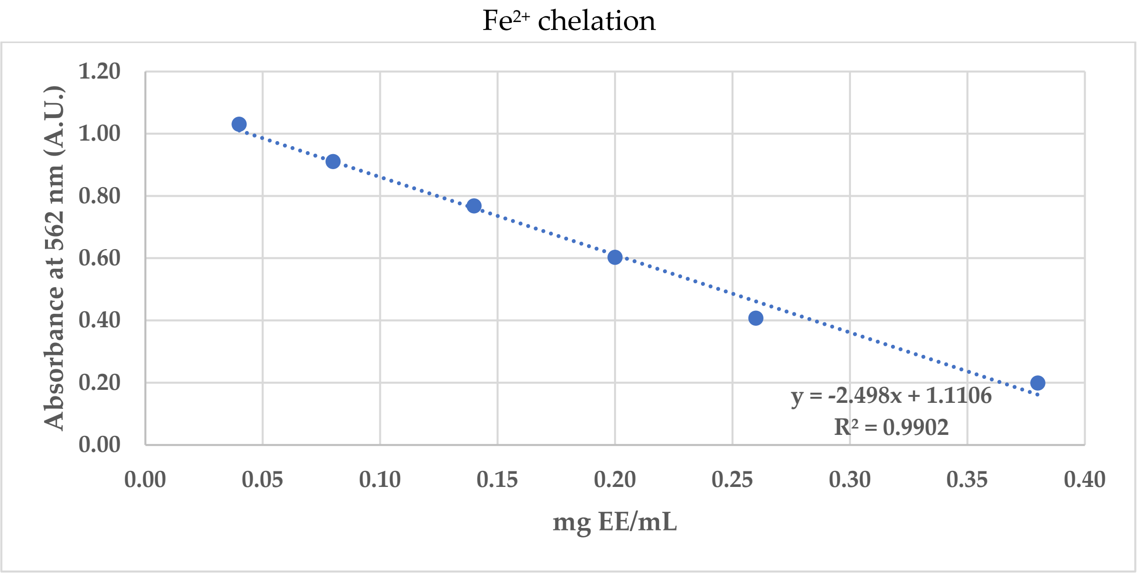

Figure S1. Calibration curves for the quantitative tests performed in this study

Table S1. Limit of detection (LOD), limit of quantification (LOQ) and recovery percentage of analyzed phenolics and fatty acids

Analysed compounds (μg/mL)	LOD	LOQ	% recovery
Catechin	0.08	0.72	N/A
Gallic acid	0.36	1.44	N/A
Rutin	0.21	0.84	N/A
Cyanidin	0.36	1.44	N/A
Linolenic acid	0.002349	0.05683	98.84

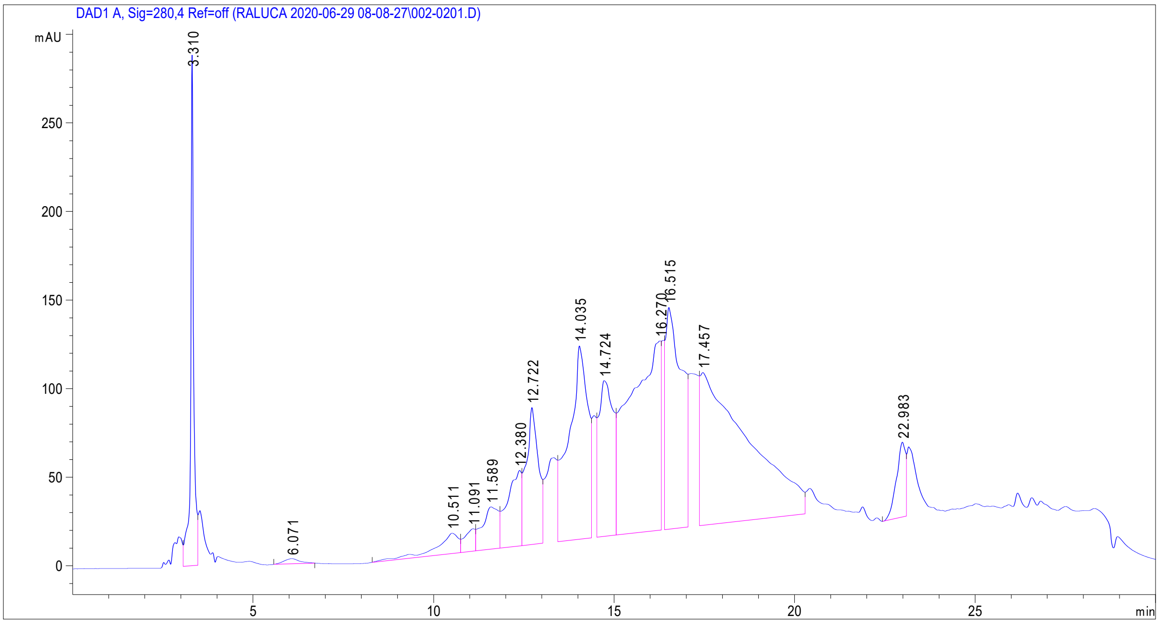

Blasius (BL)-280 nm

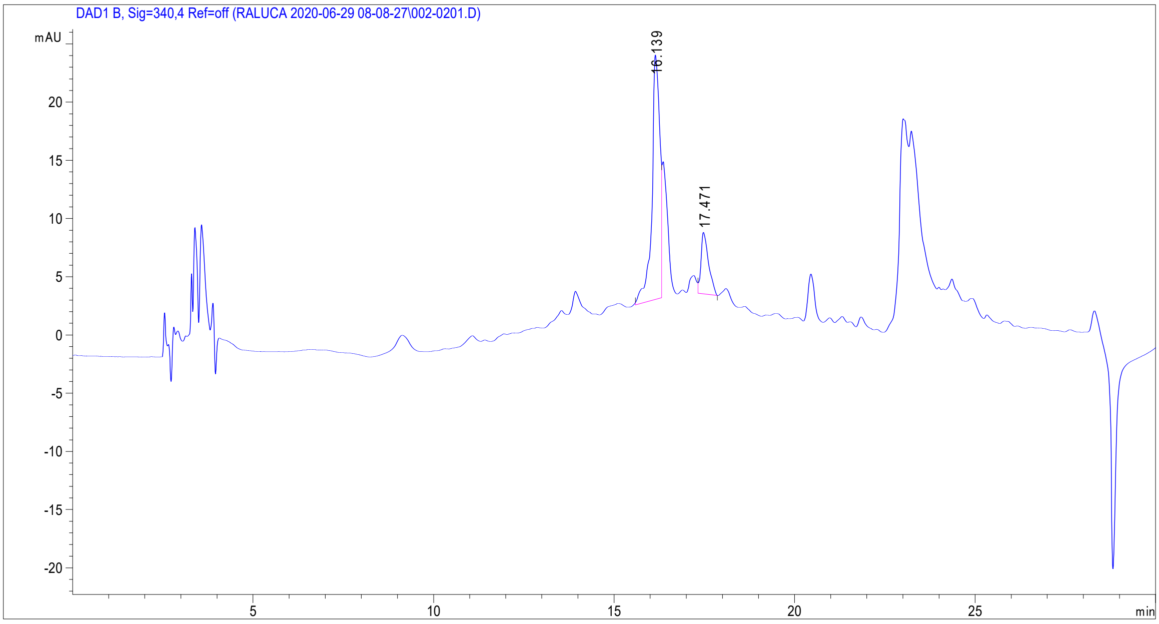

Blasius (BL)-340 nm

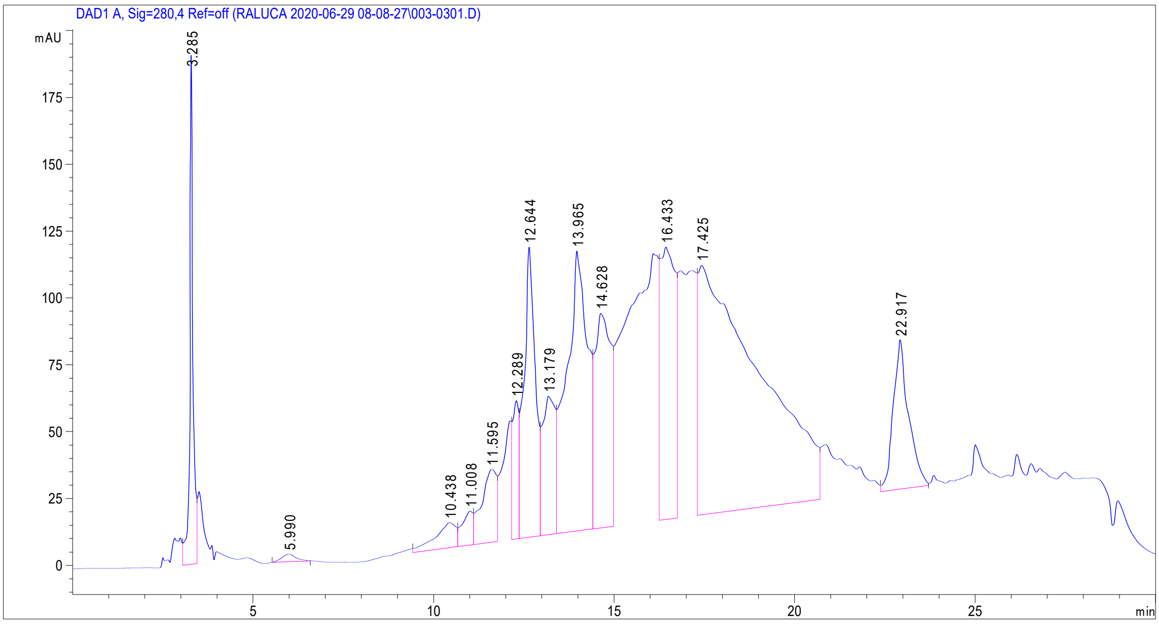

Riesling de Rhin (RR) -280 nm

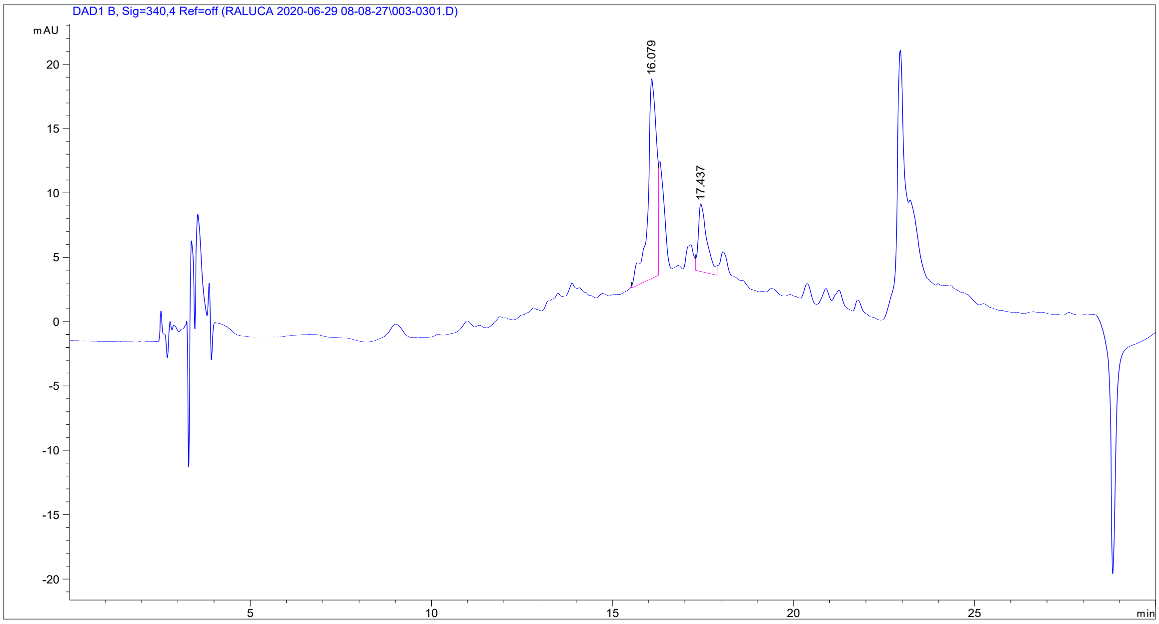

Riesling de Rhin (RR)-340 nm

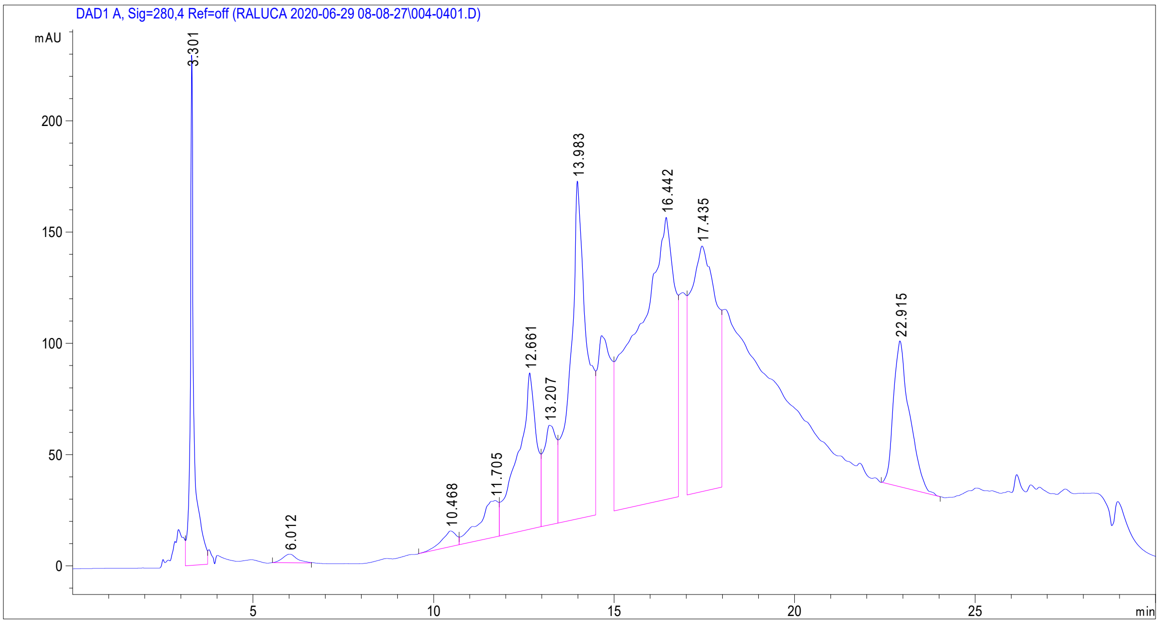

Amurg (AM)-280 nm

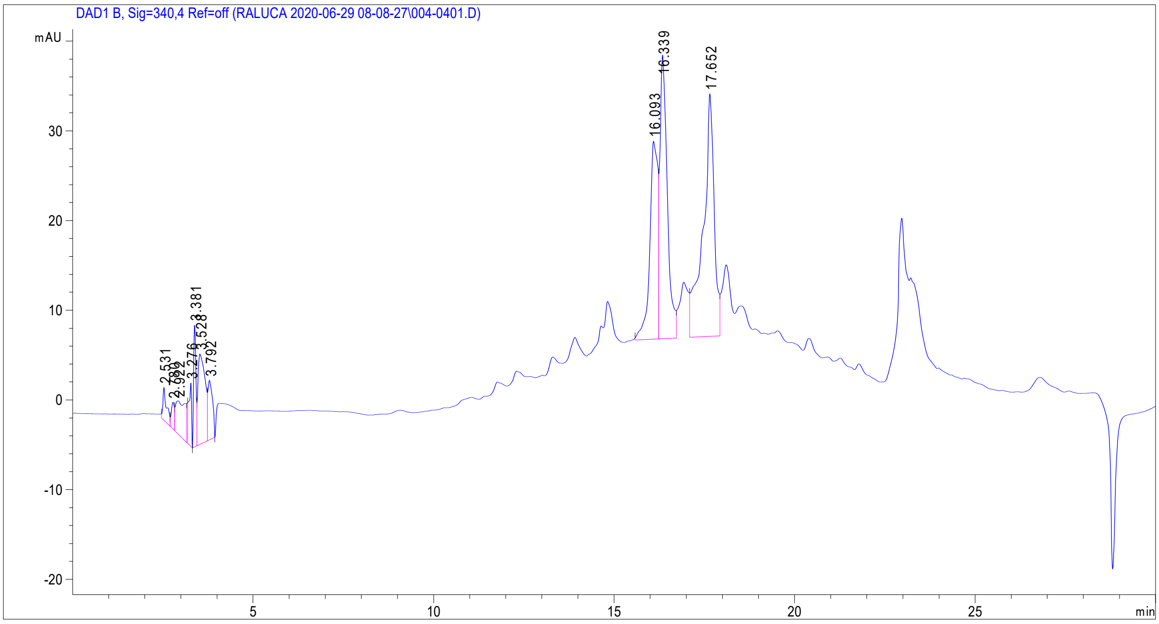

Amurg (AM)-340 nm

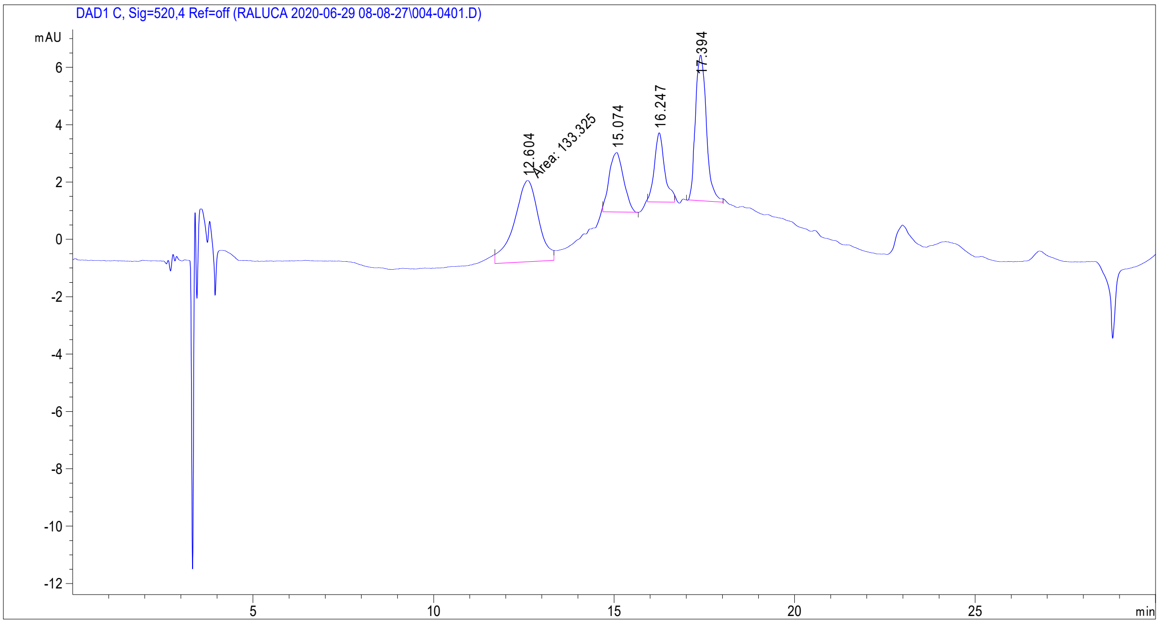

Amurg (AM)-520 nm

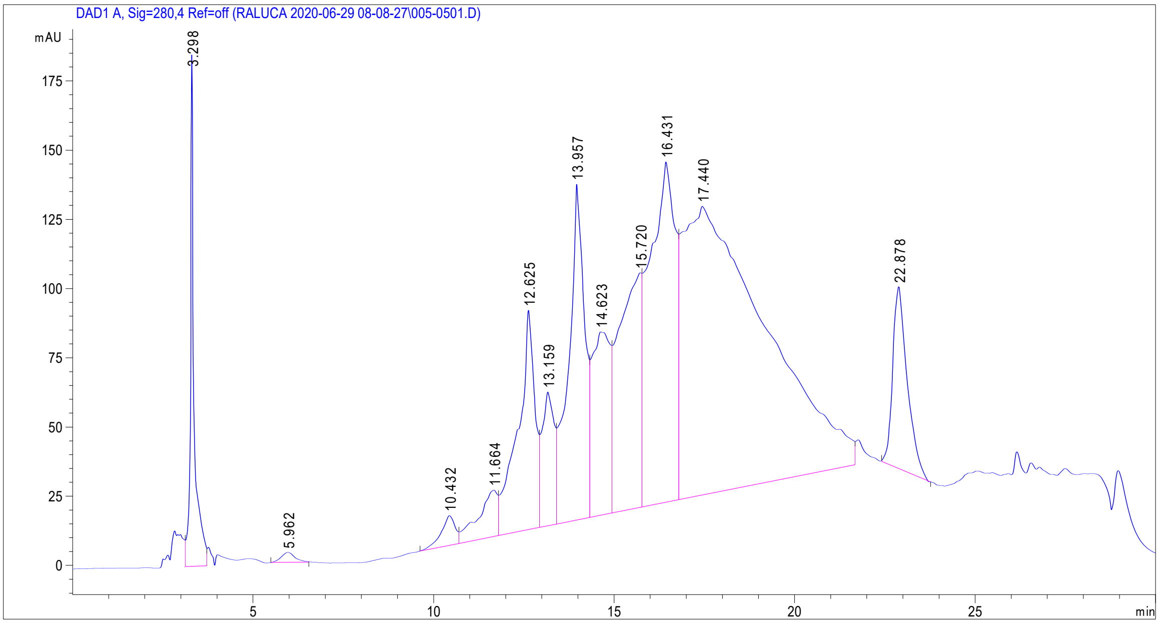

Roze Blaj (RB)-280 nm

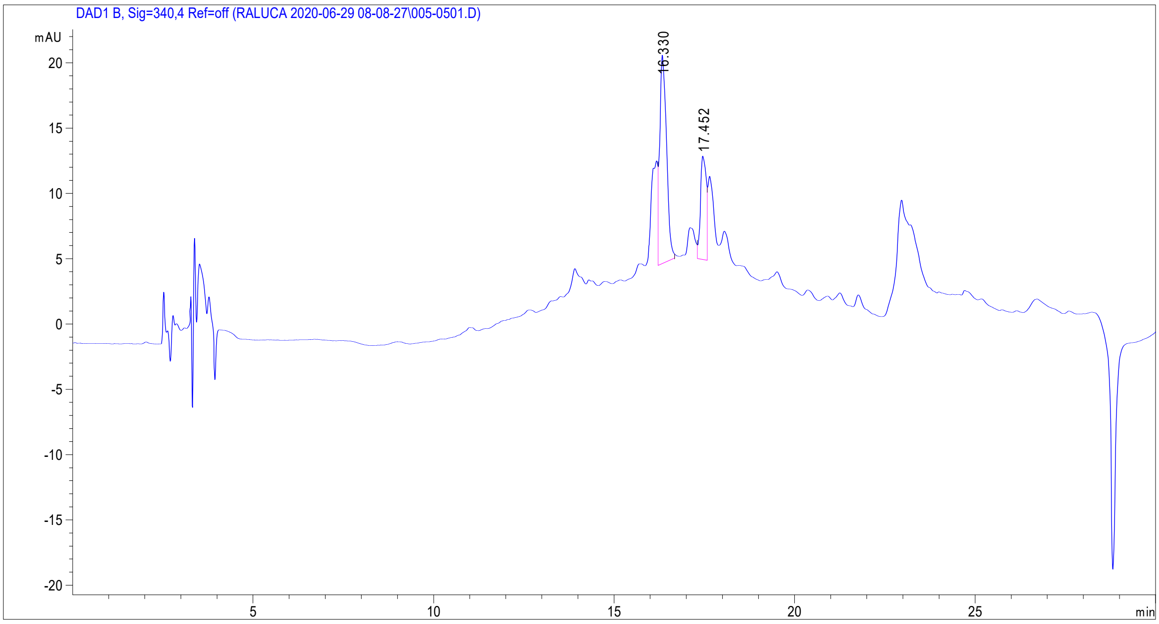

Roze Blaj (RB)-340 nm

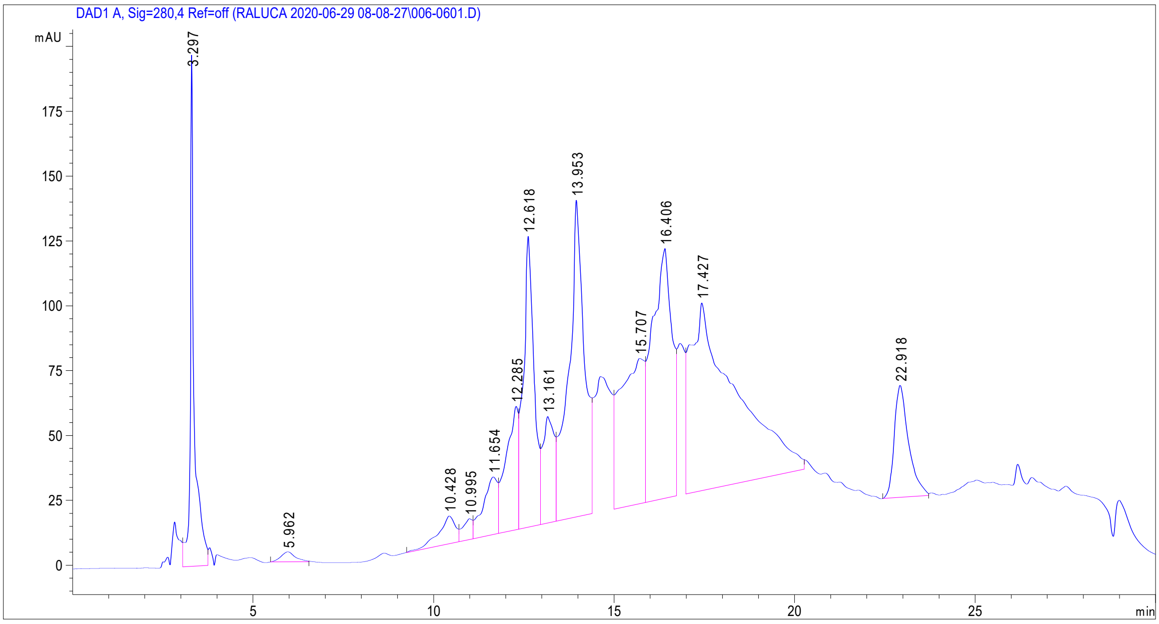

Astra-280 nm (AS)

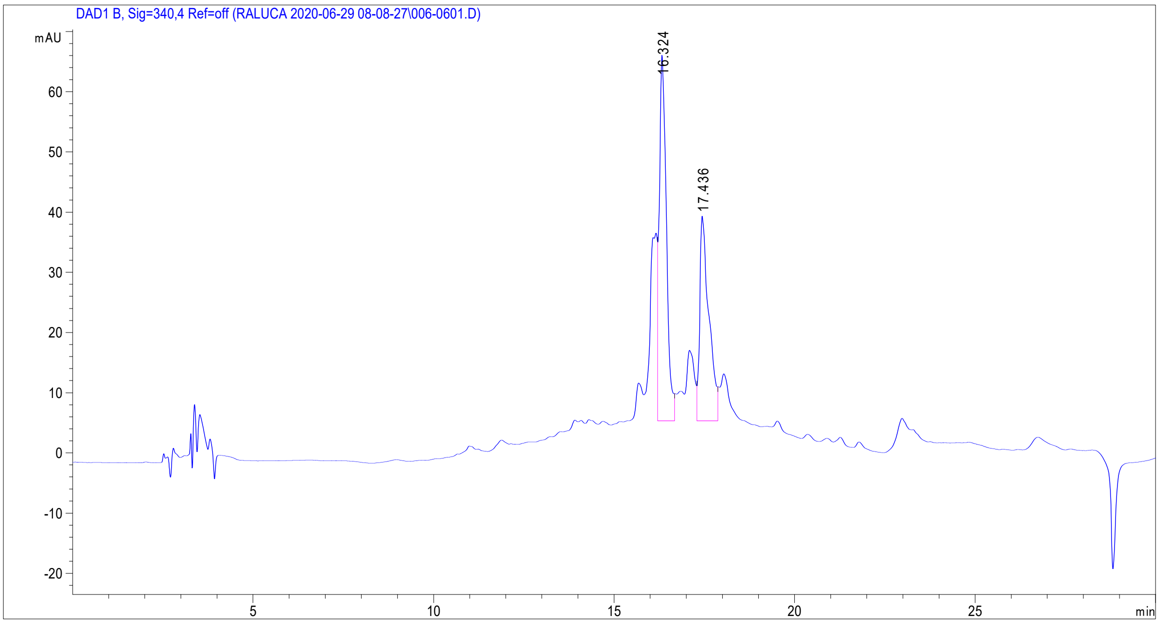

Astra (AS)-340 nm

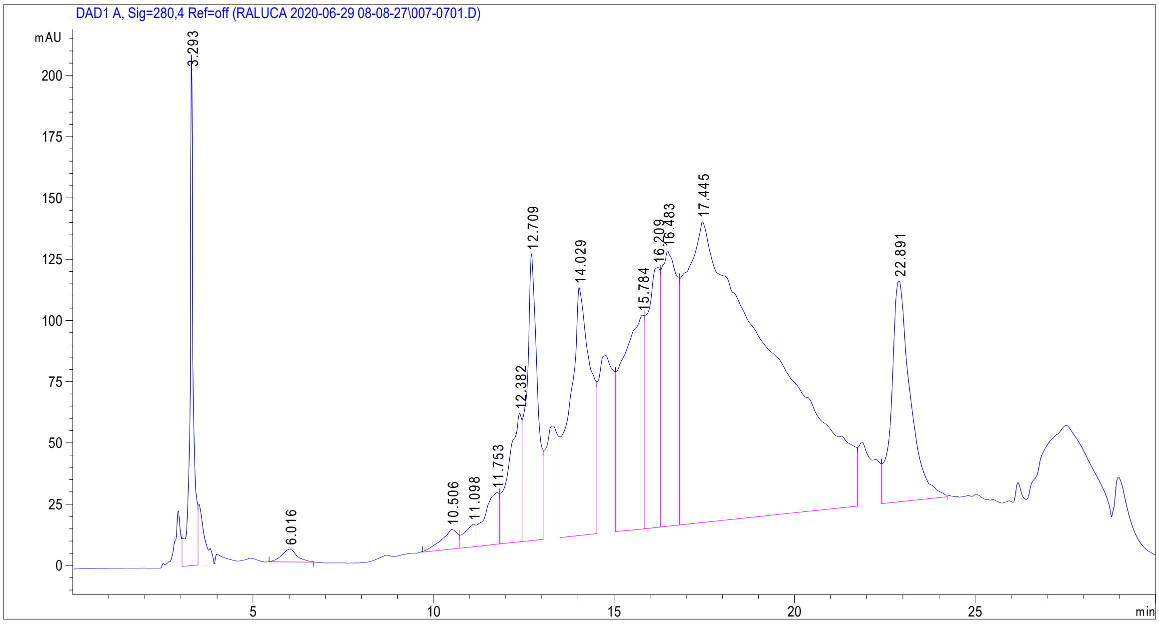

Syrah (SH)-280 nm

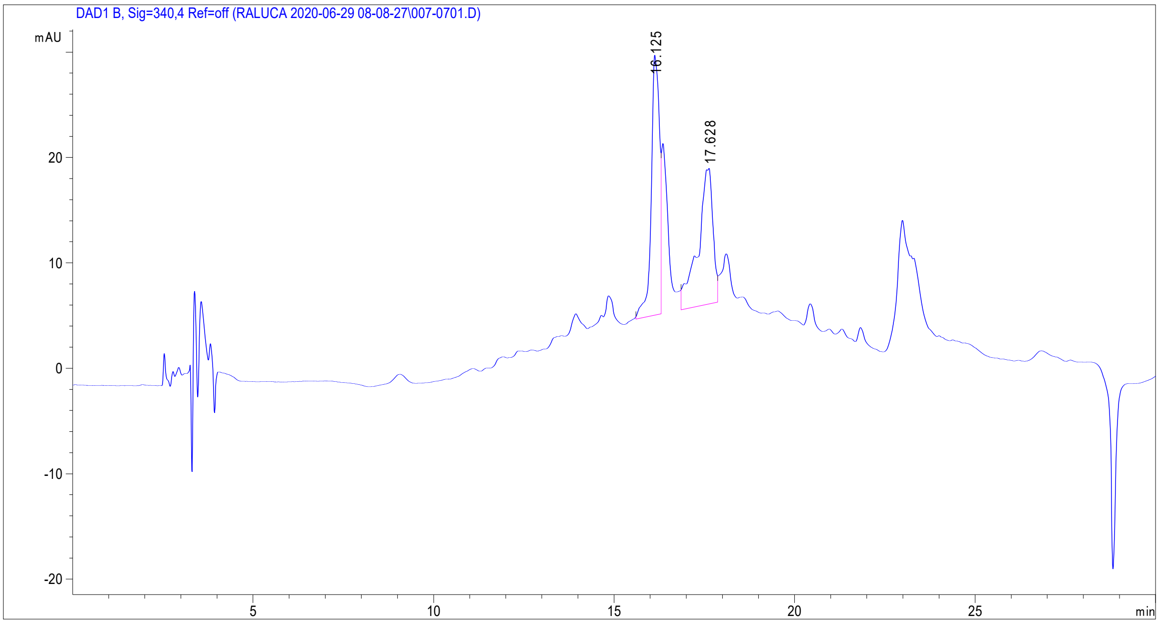

Syrah (SH)-340 nm

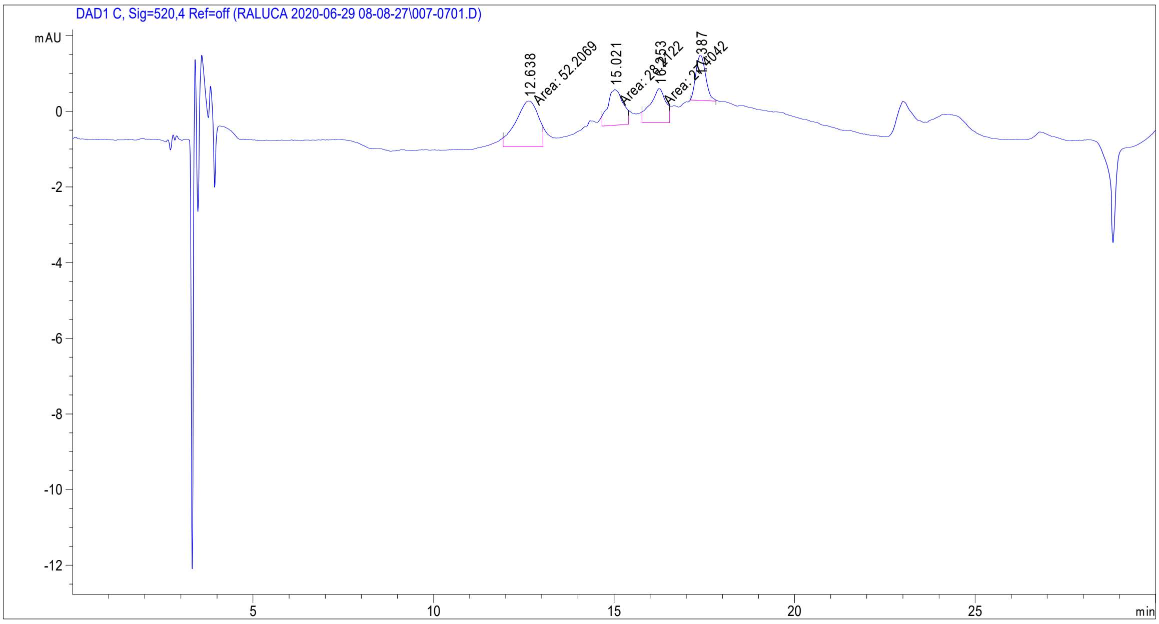

Syrah (SH)-520 nm

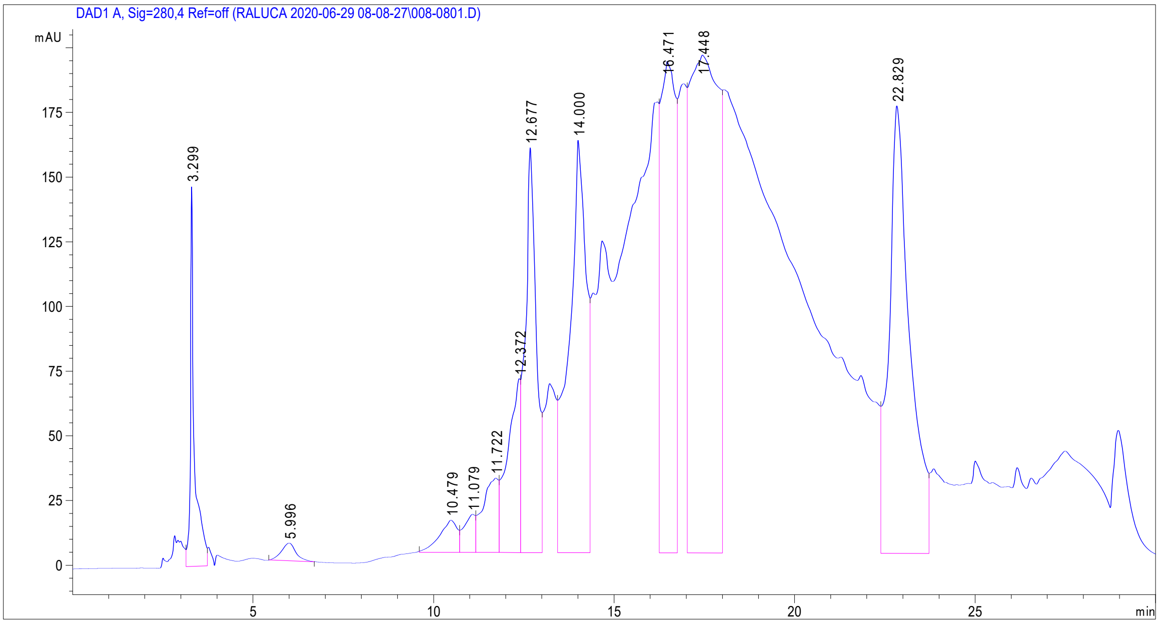

Traminer roz (TR)-280 nm

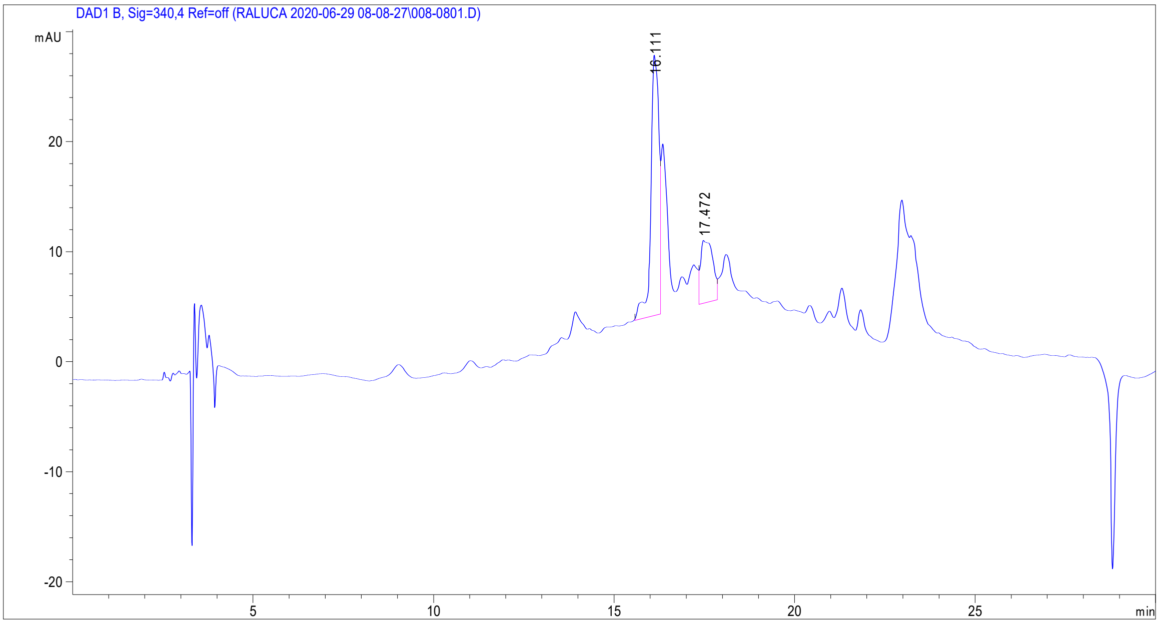

Traminer roz (TR)-340 nm

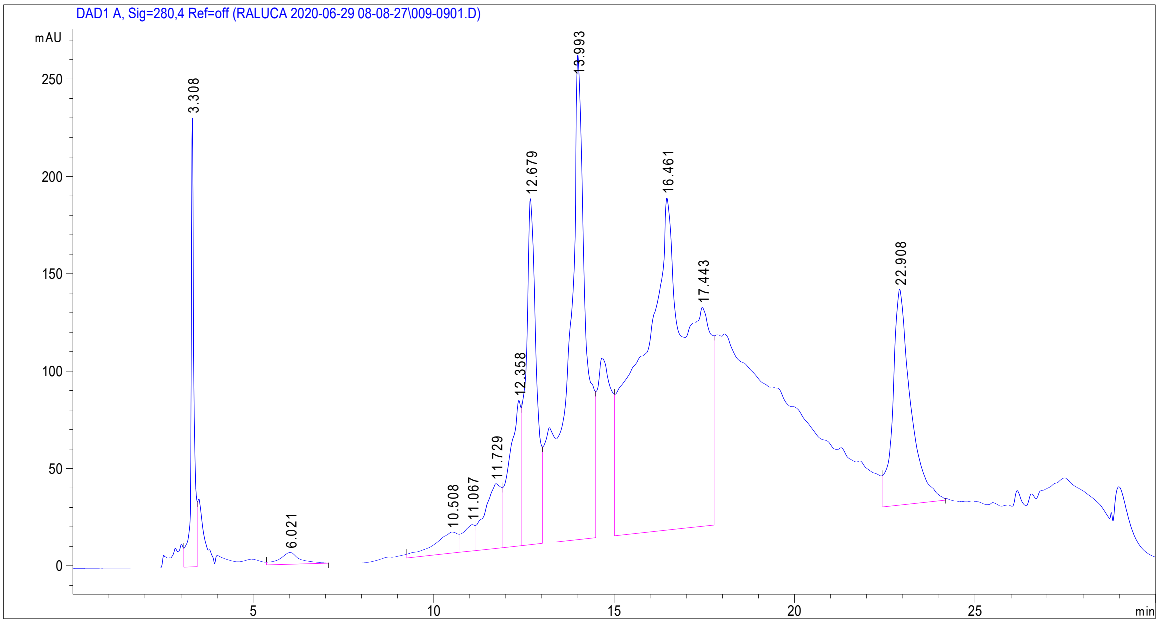

Brumăriu (BR)-280 nm

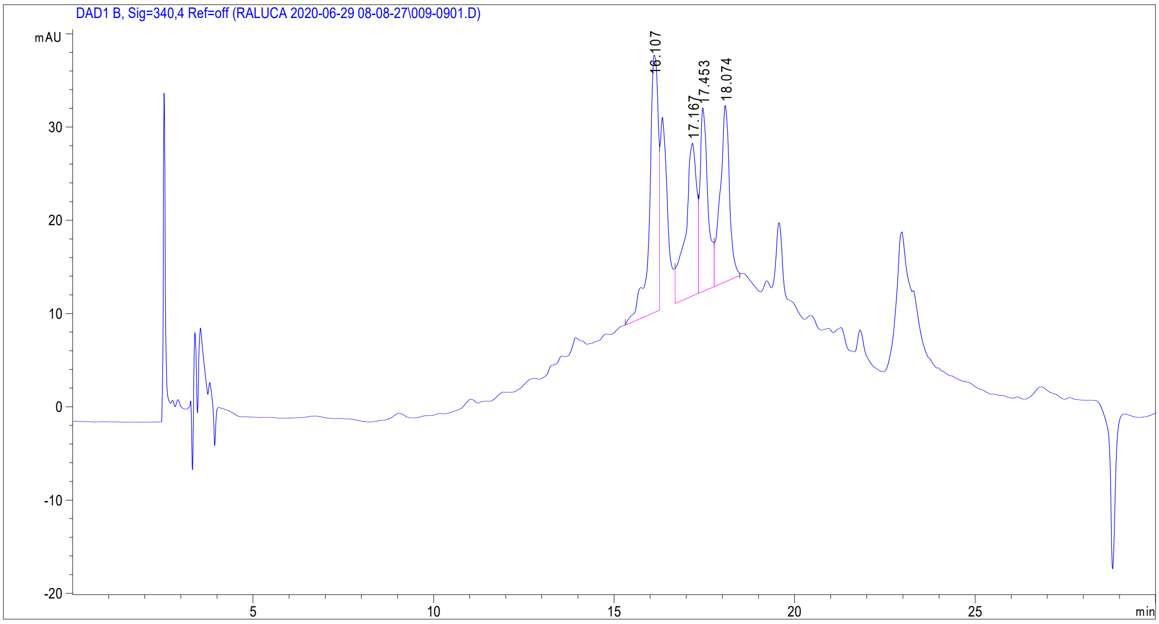

Brumăriu (BR)-340 nm

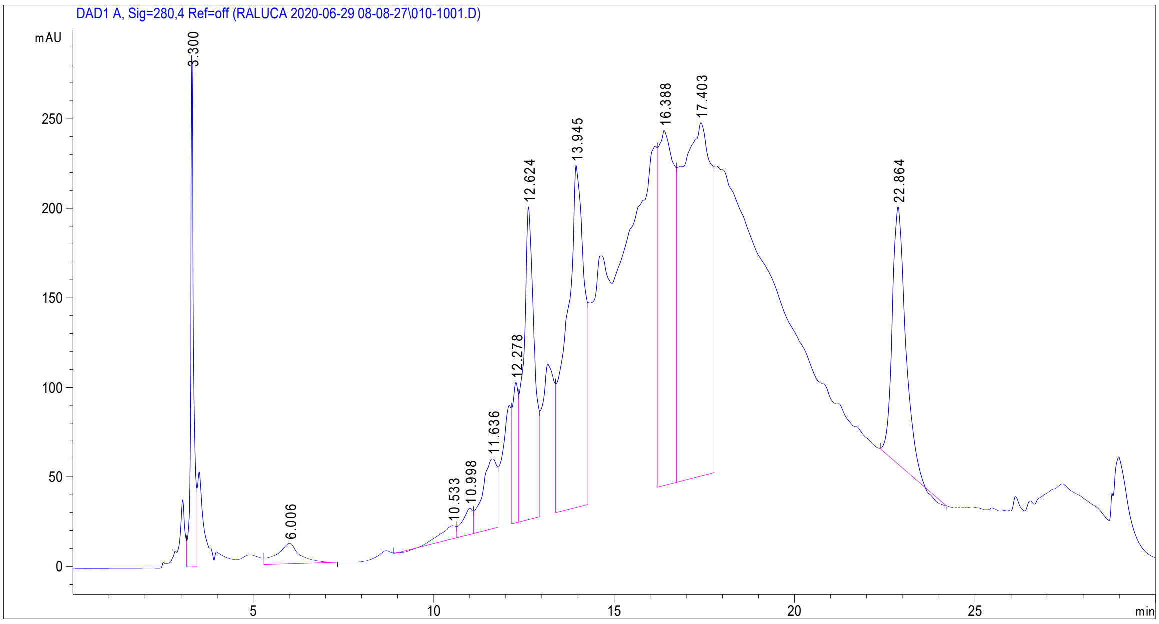

Johanniter (JO)-280 nm

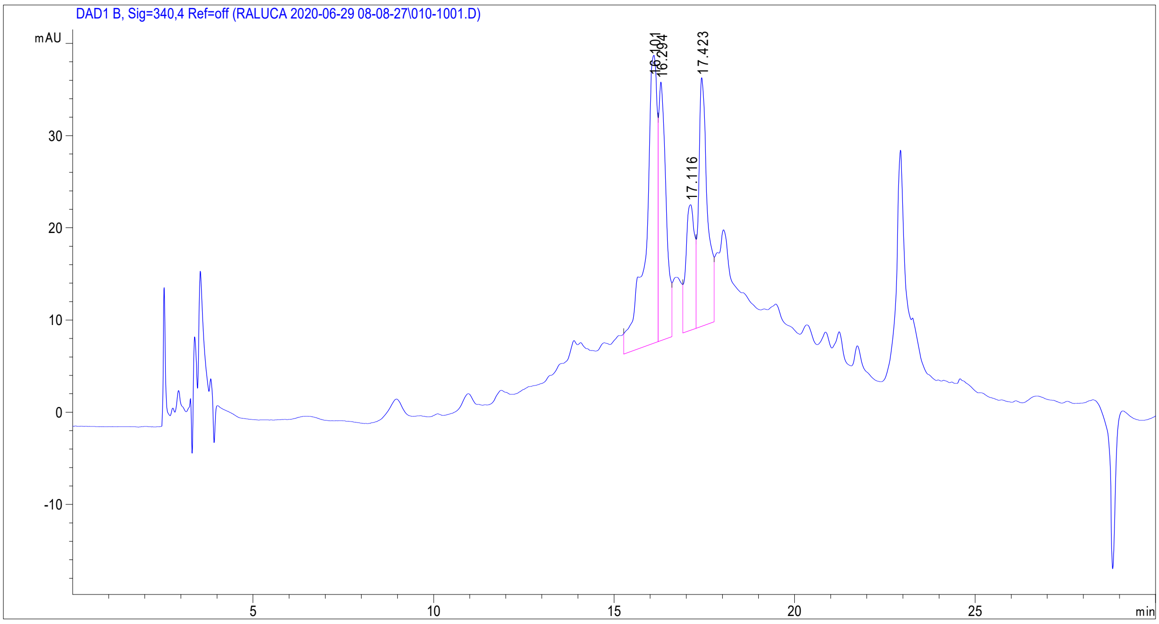

Johanniter (JO)-340 nm

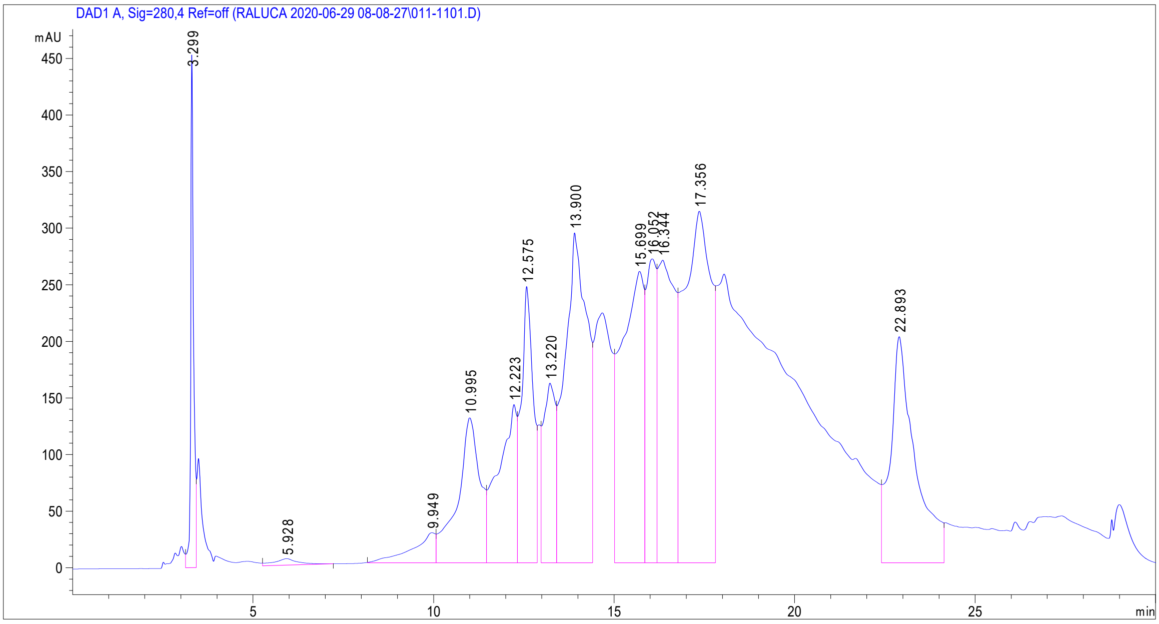

Regent (RE)-280 nm

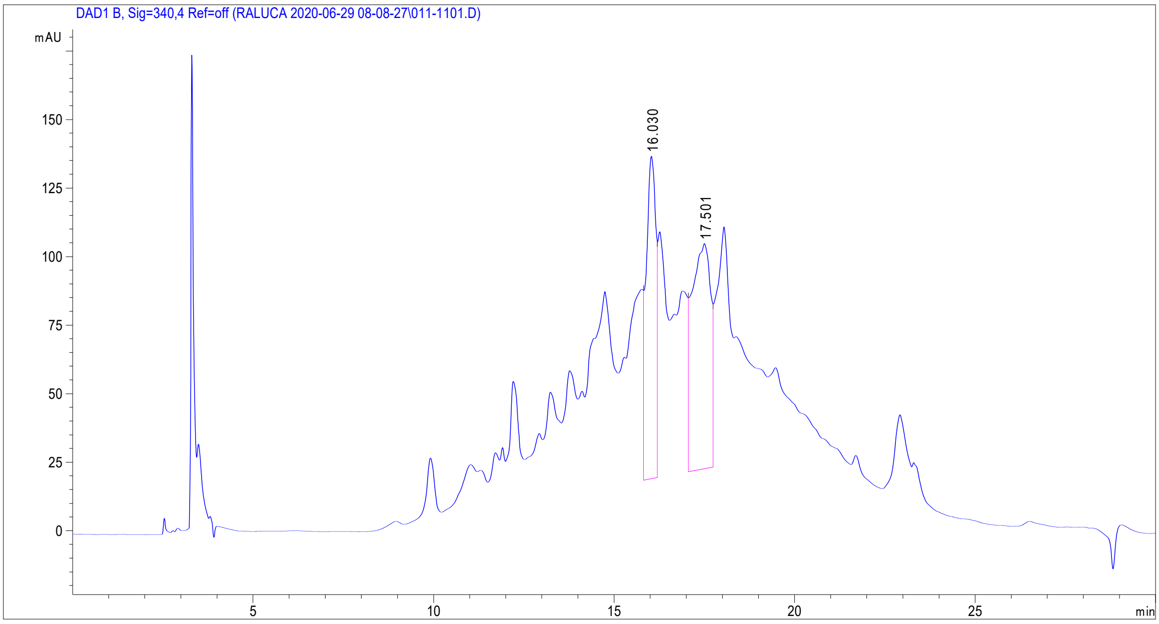

Regent (RE)-340 nm

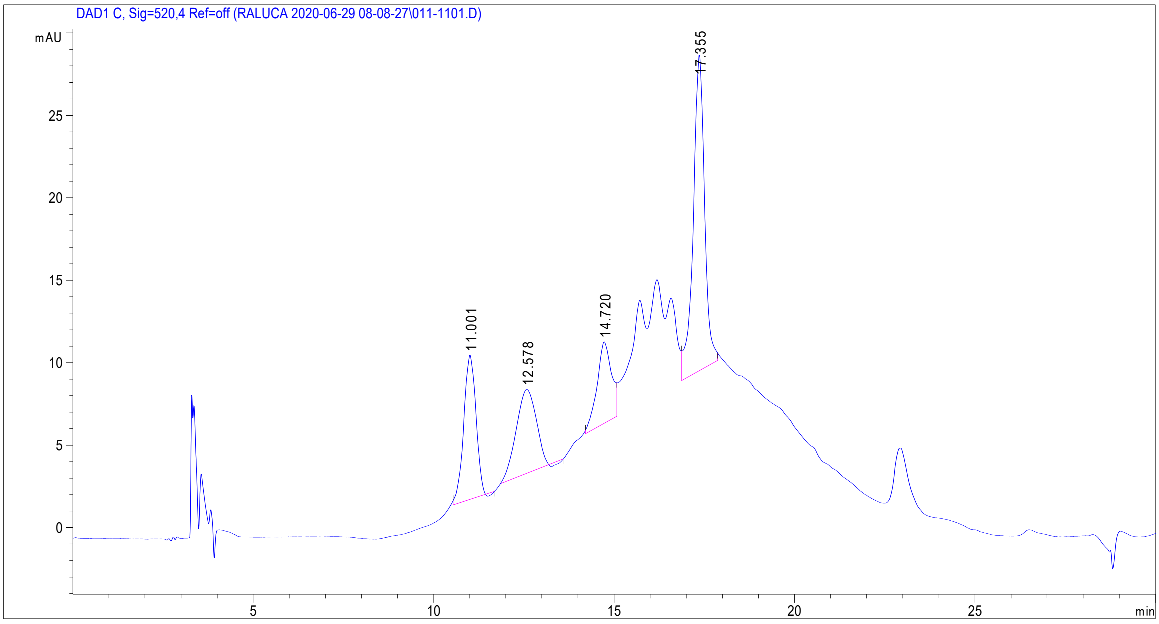

Regent (RE)-520 nm

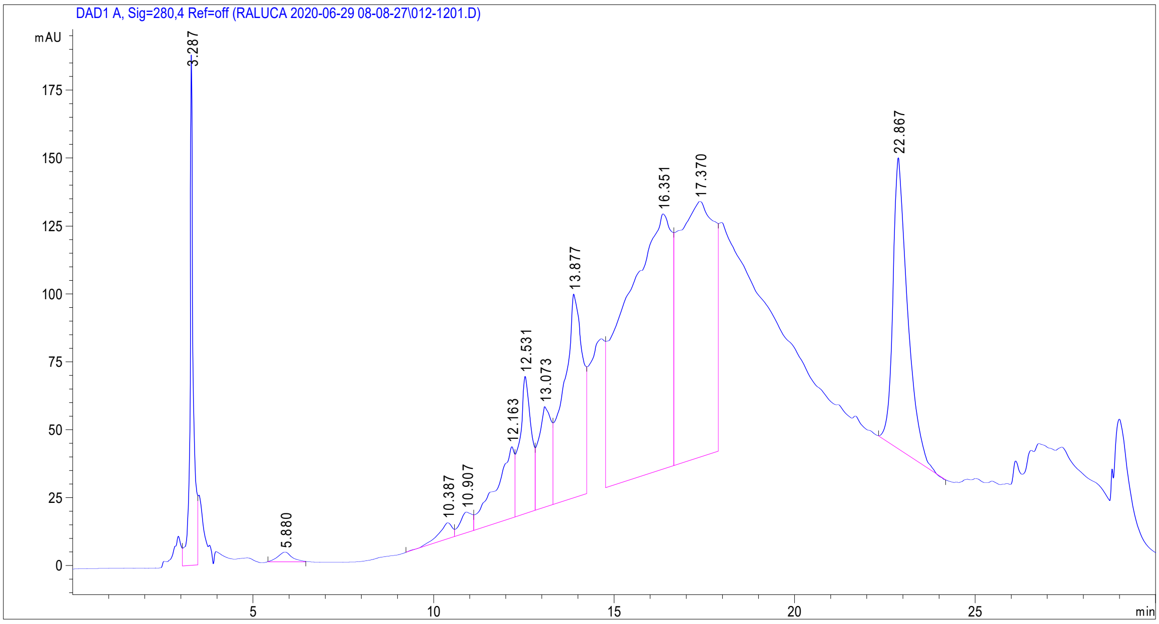

Selena (SE)-280 nm

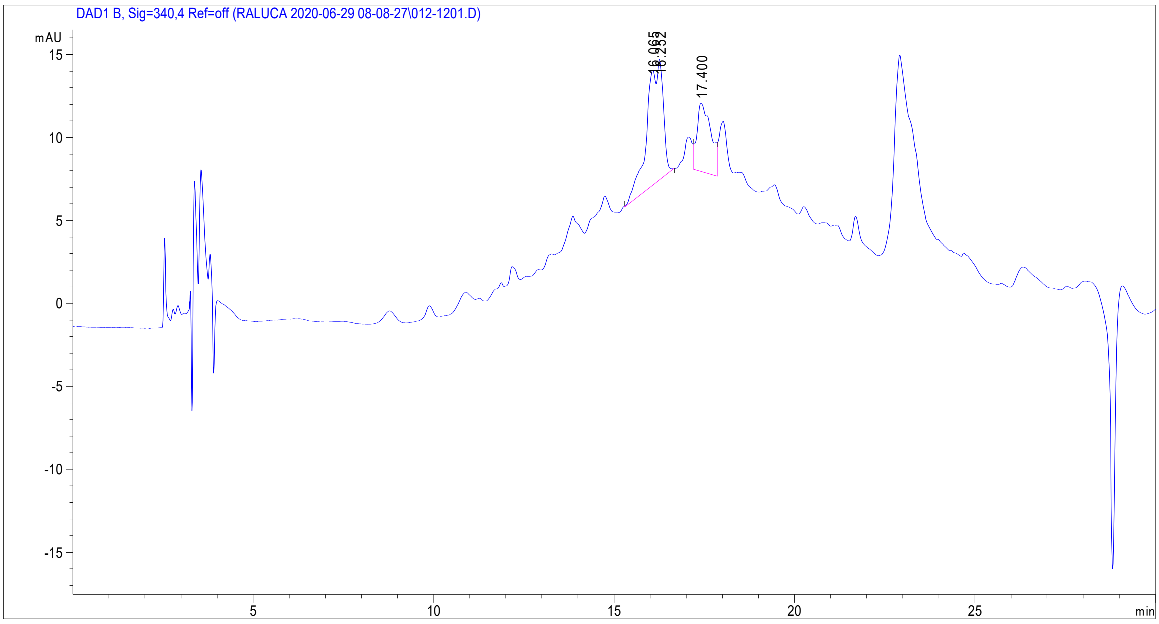

Selena (SE)-340 nm

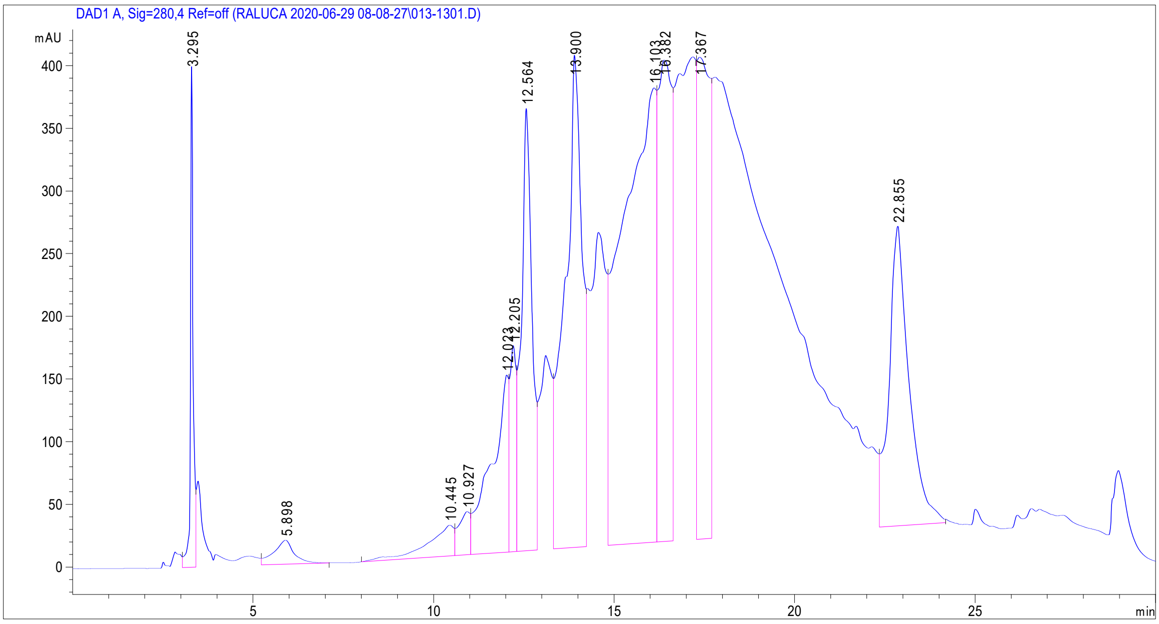

Neuburger (NE)-280 nm

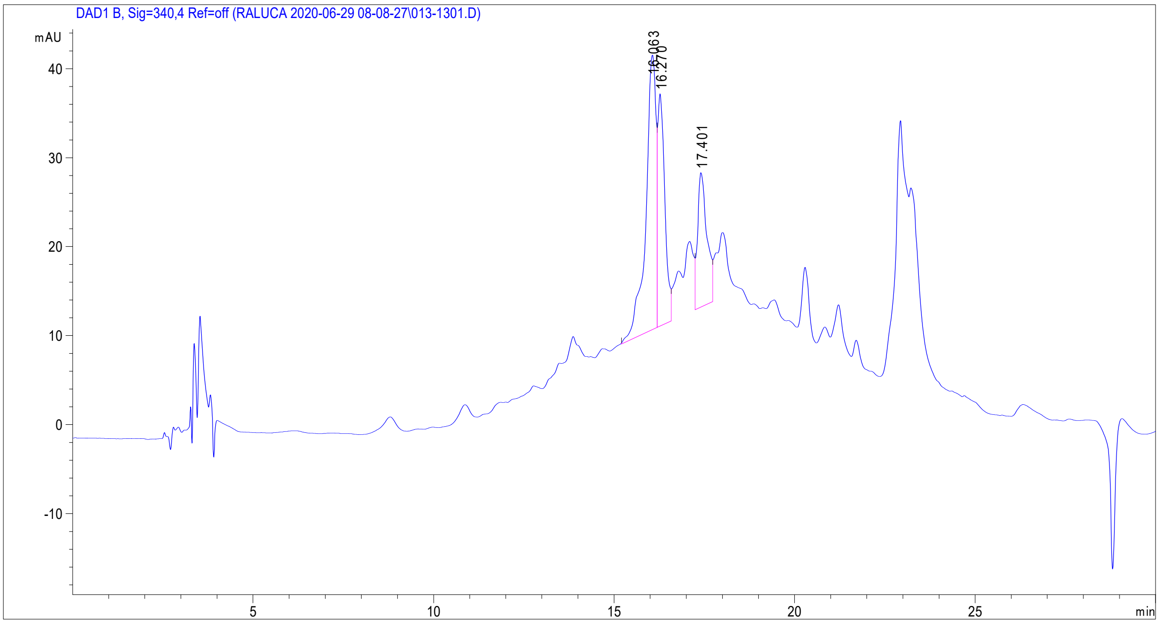

Neuburger (NE)-340 nm

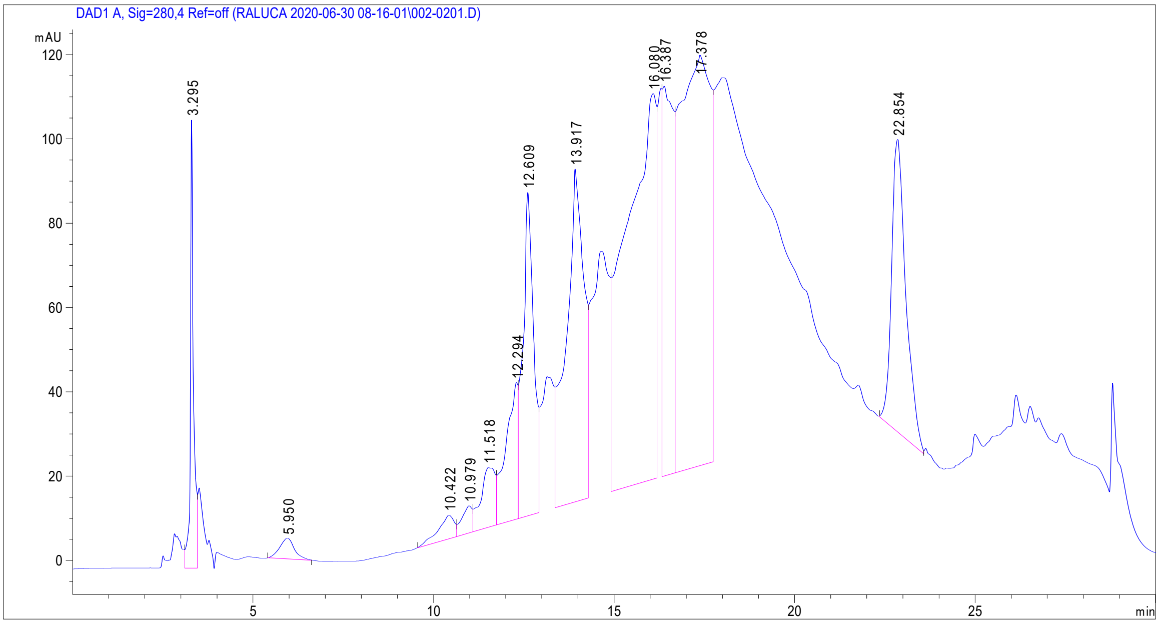

Muscat Ottonel (MO)-280 nm

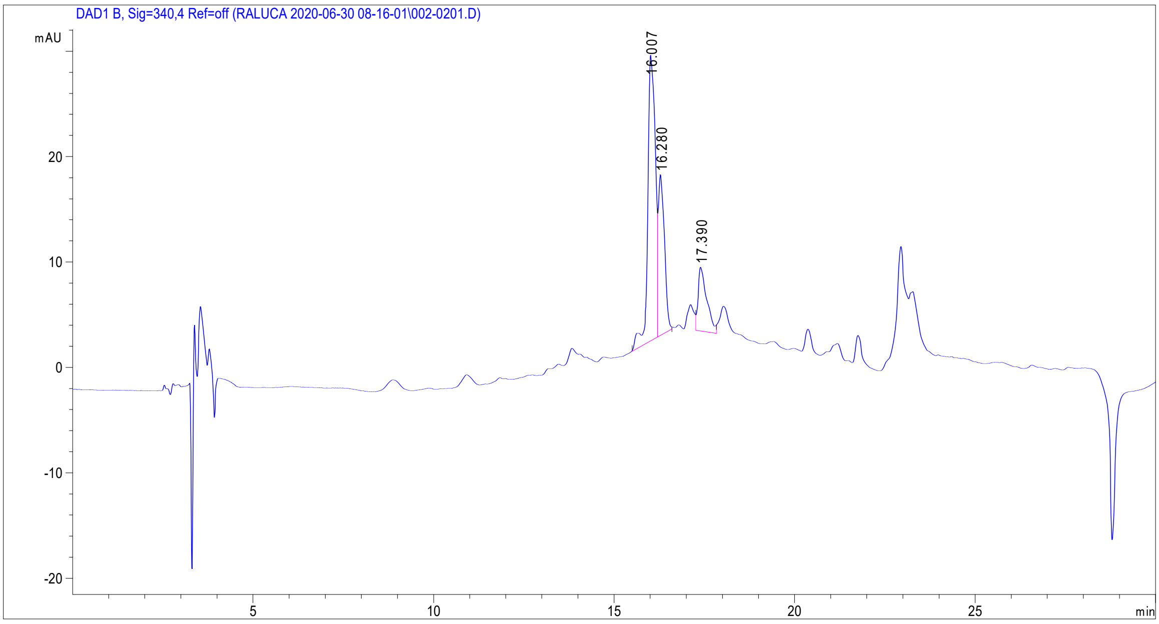

Muscat Ottonel (MO)-340 nm

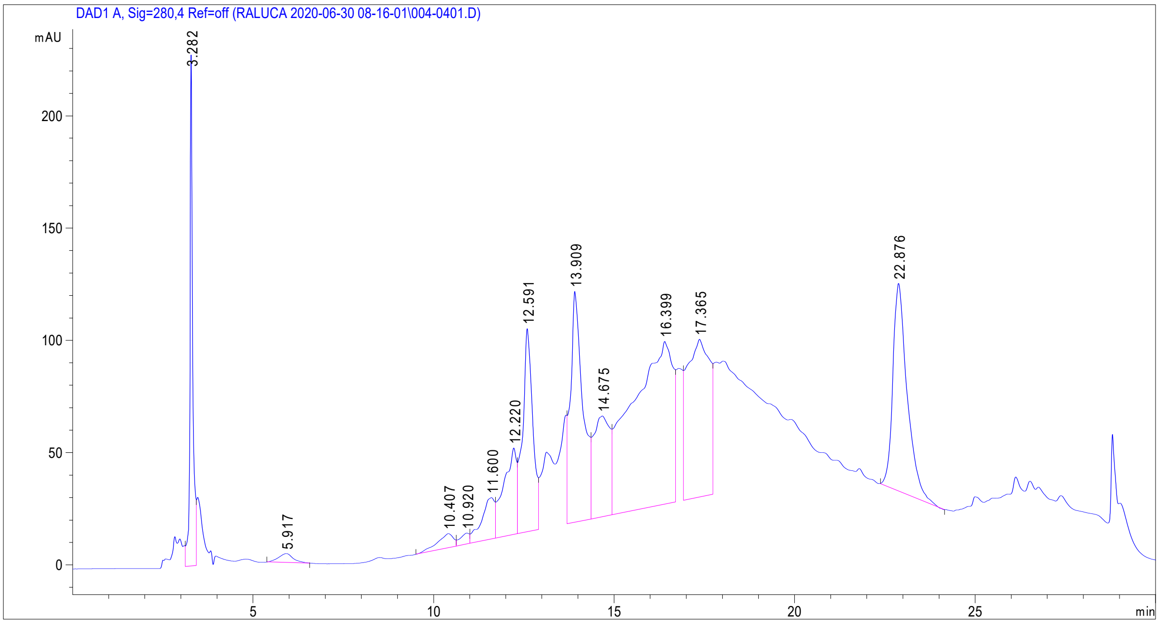

Rubin (RU)-280 nm

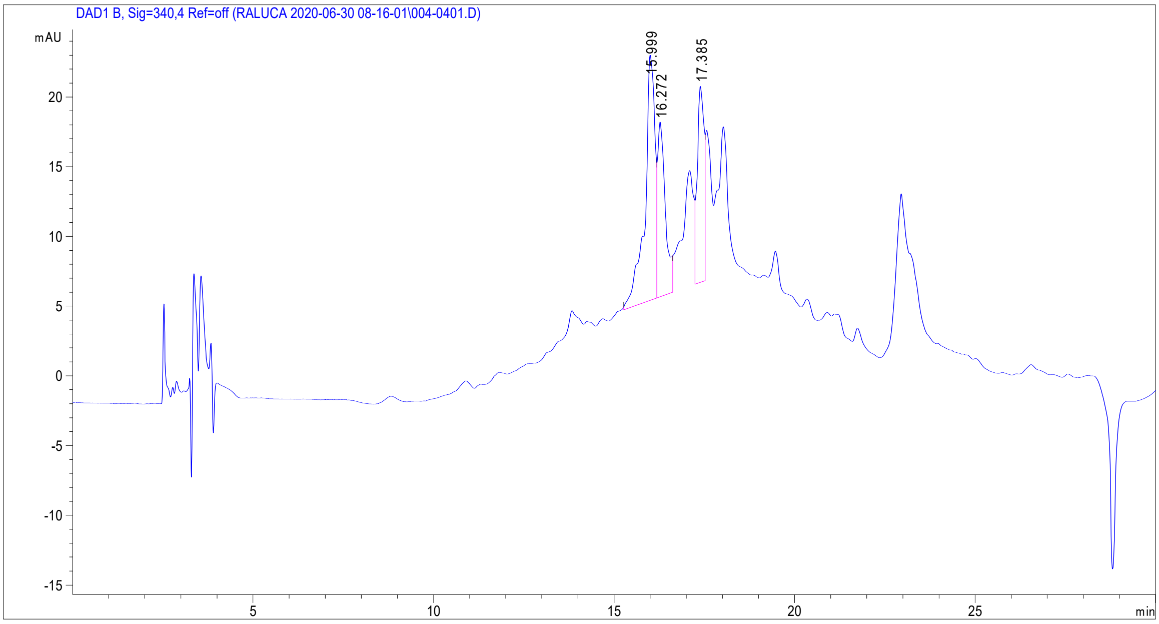

Rubin (RU)-340 nm

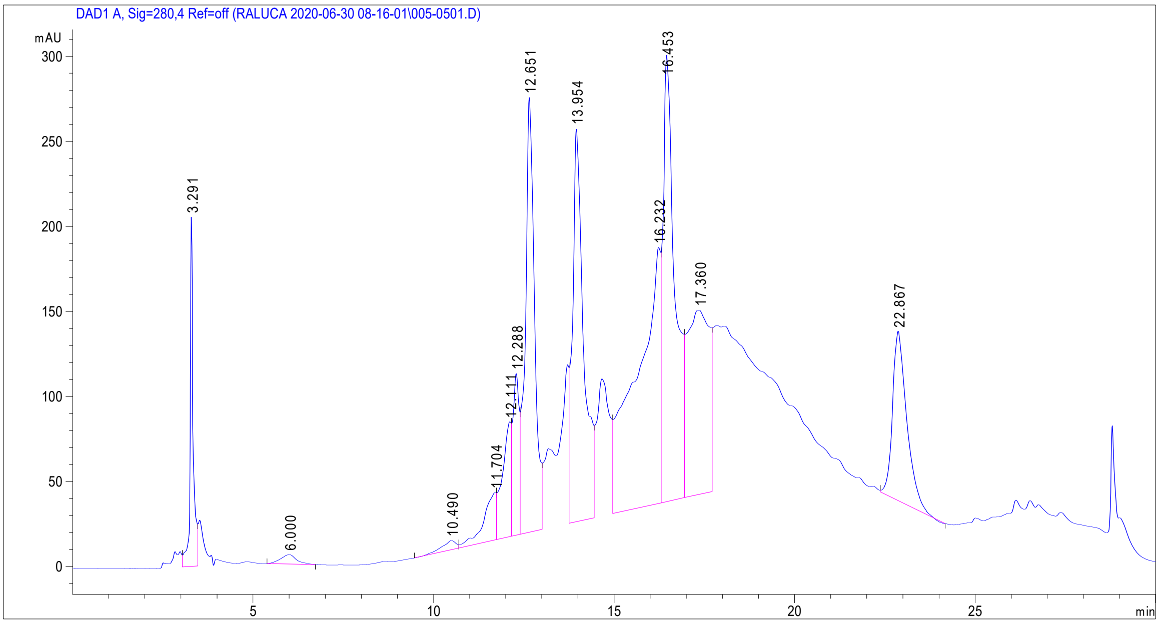

Radames (RA)-280 nm

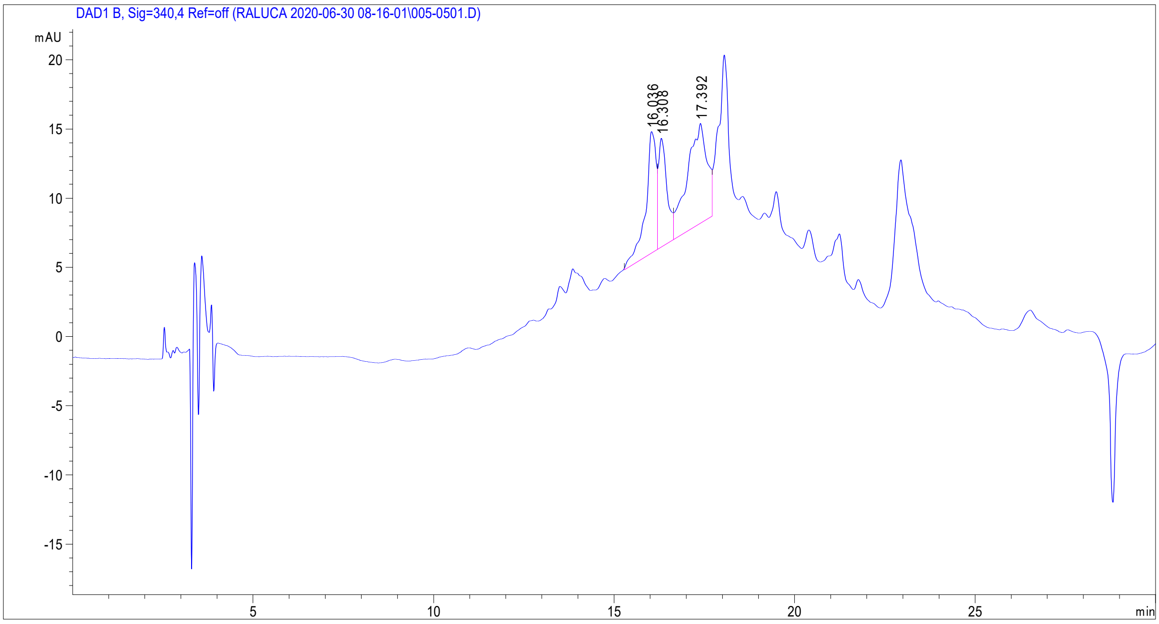

Radames (RA)-340 nm

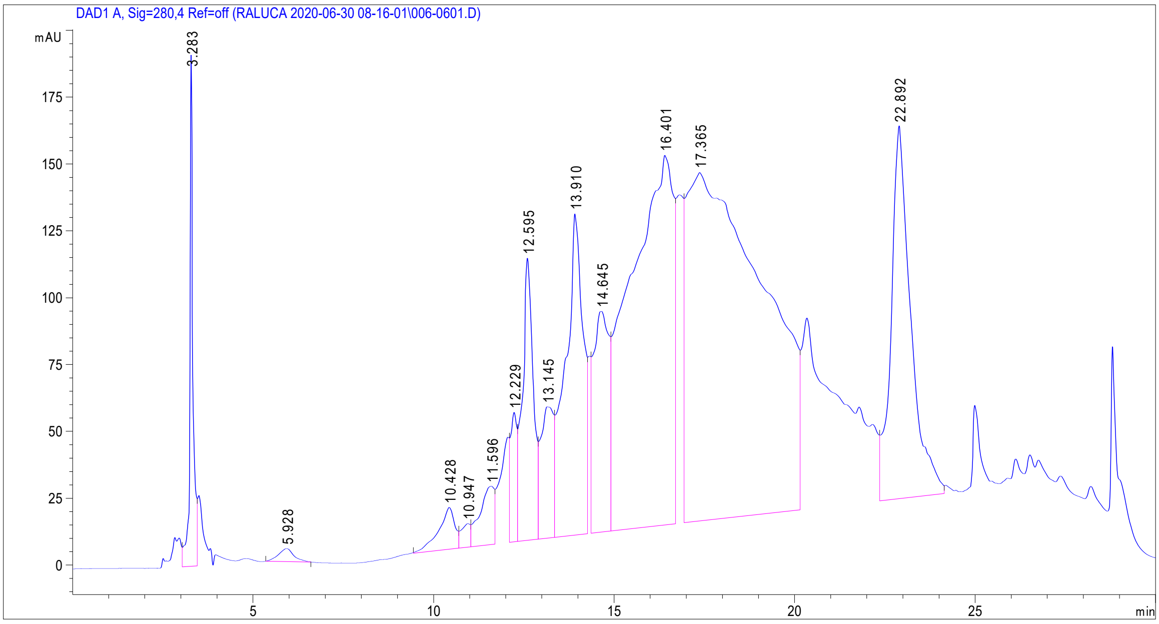

Sauvignon blanc (SB)-280 nm

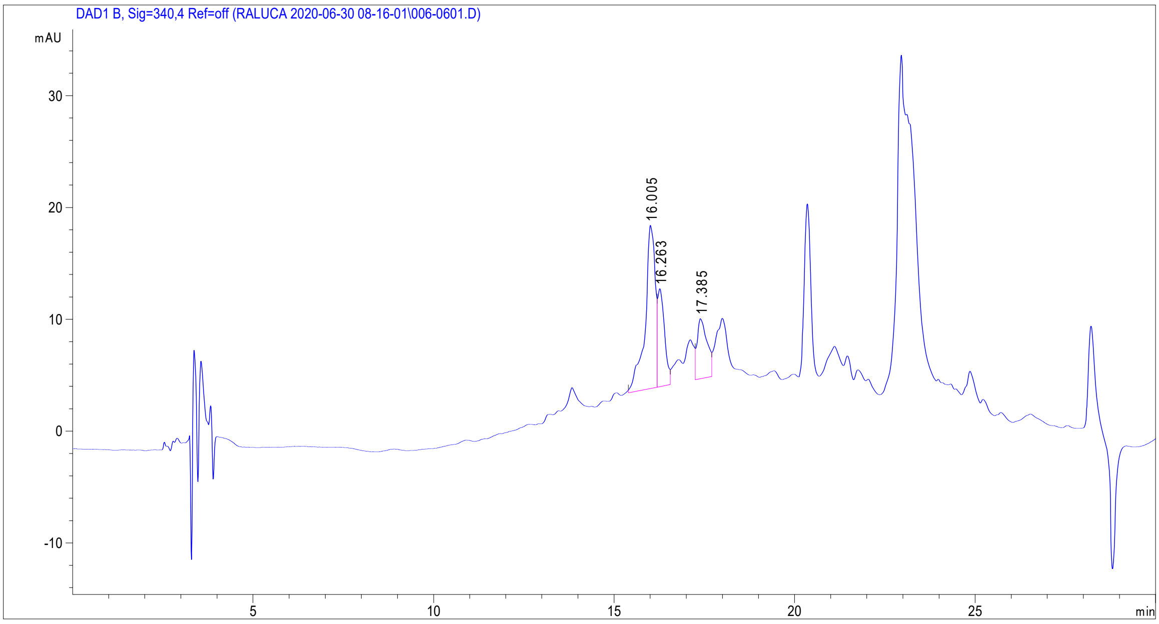

Sauvignon blanc (SB) -340 nm

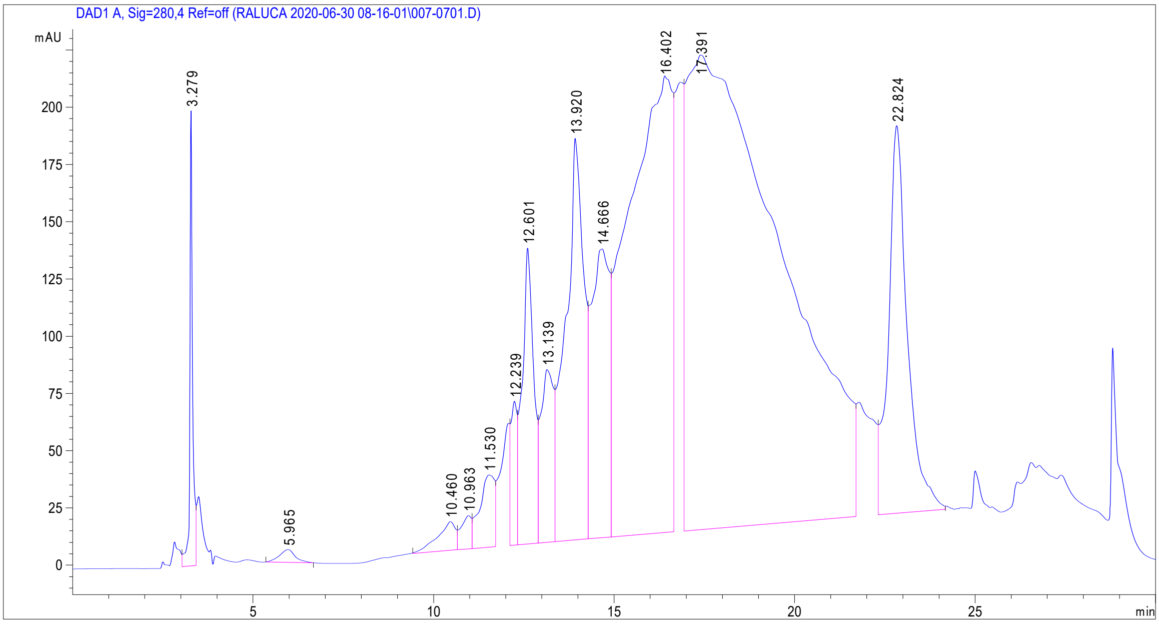

Fetească Regală (FR)-280 nm

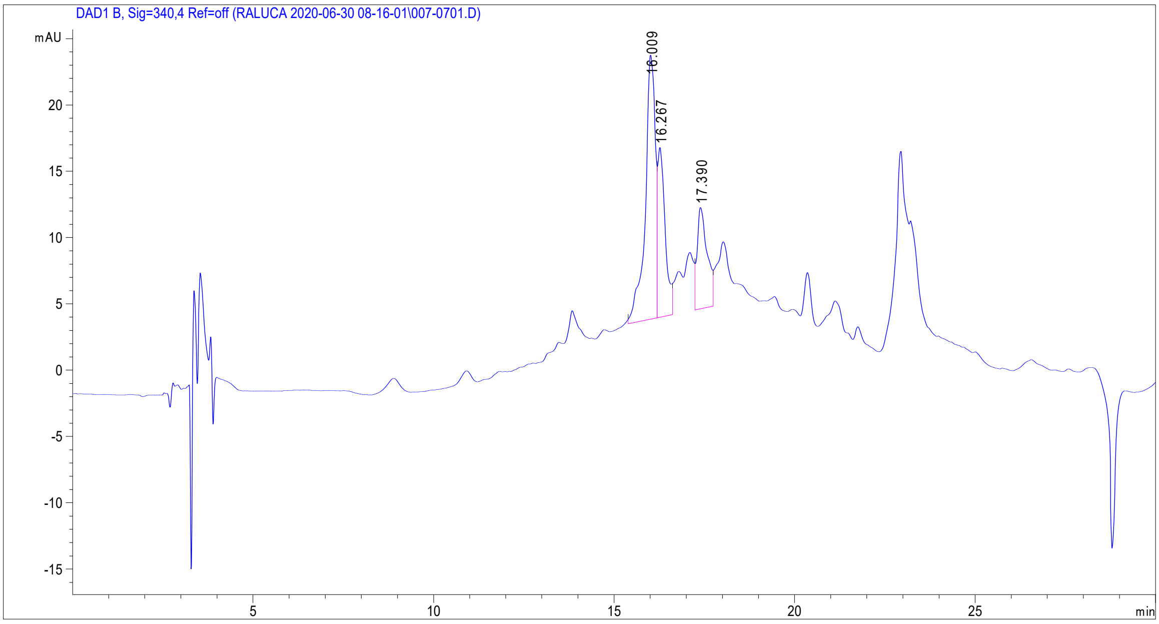

Fetească Regală -340 nm (FR)

Figure S2. Chromatograms of phenolic extracts at 280 nm, 340 nm and 520 nm (for red GPs)

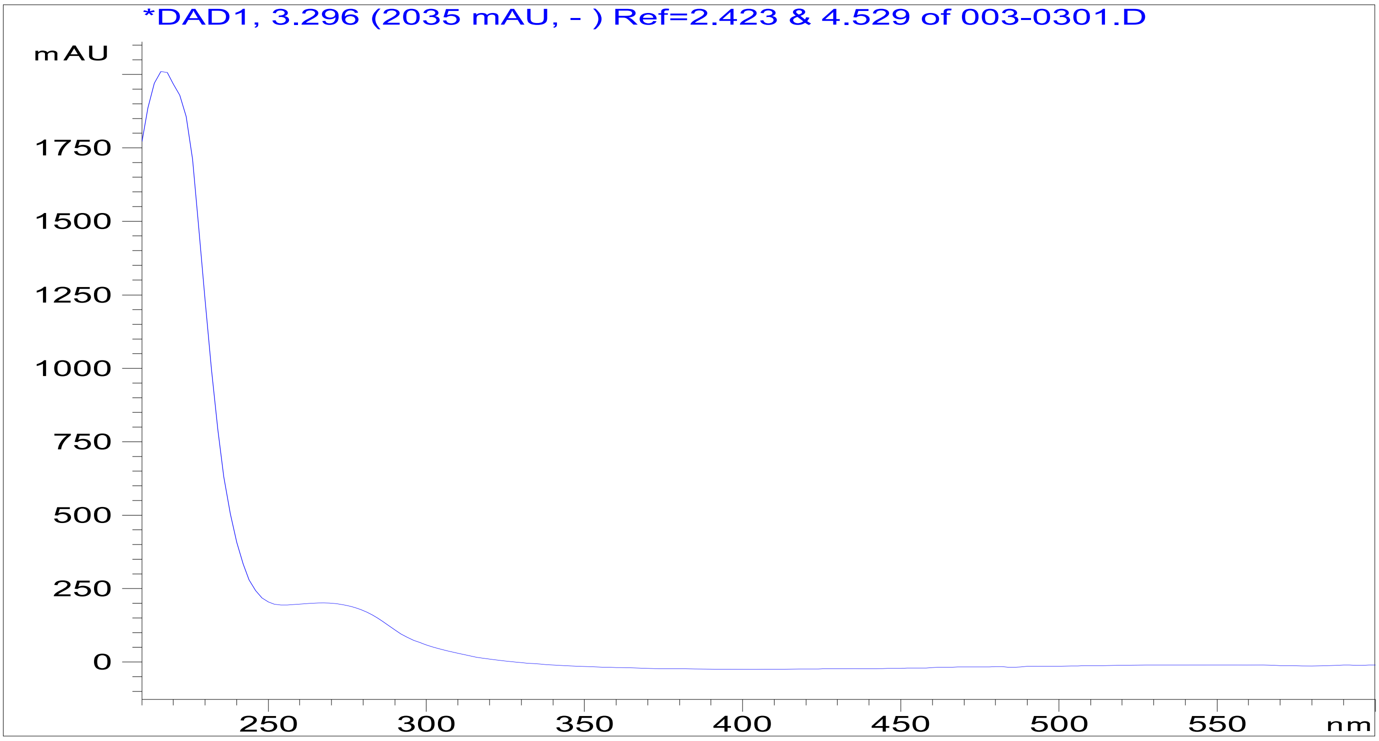

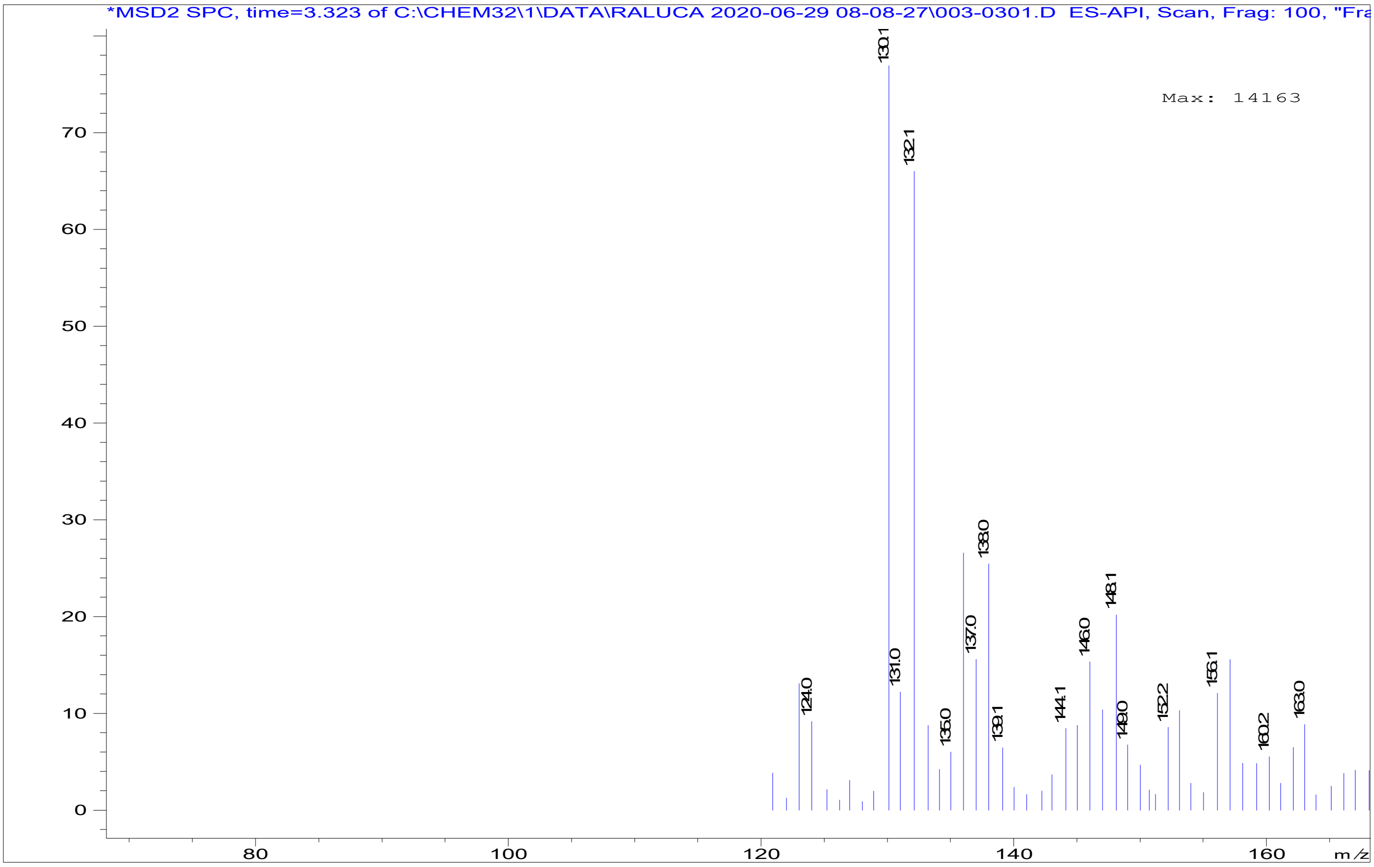

Rt=3.39 min Hydroxybenzoic acid

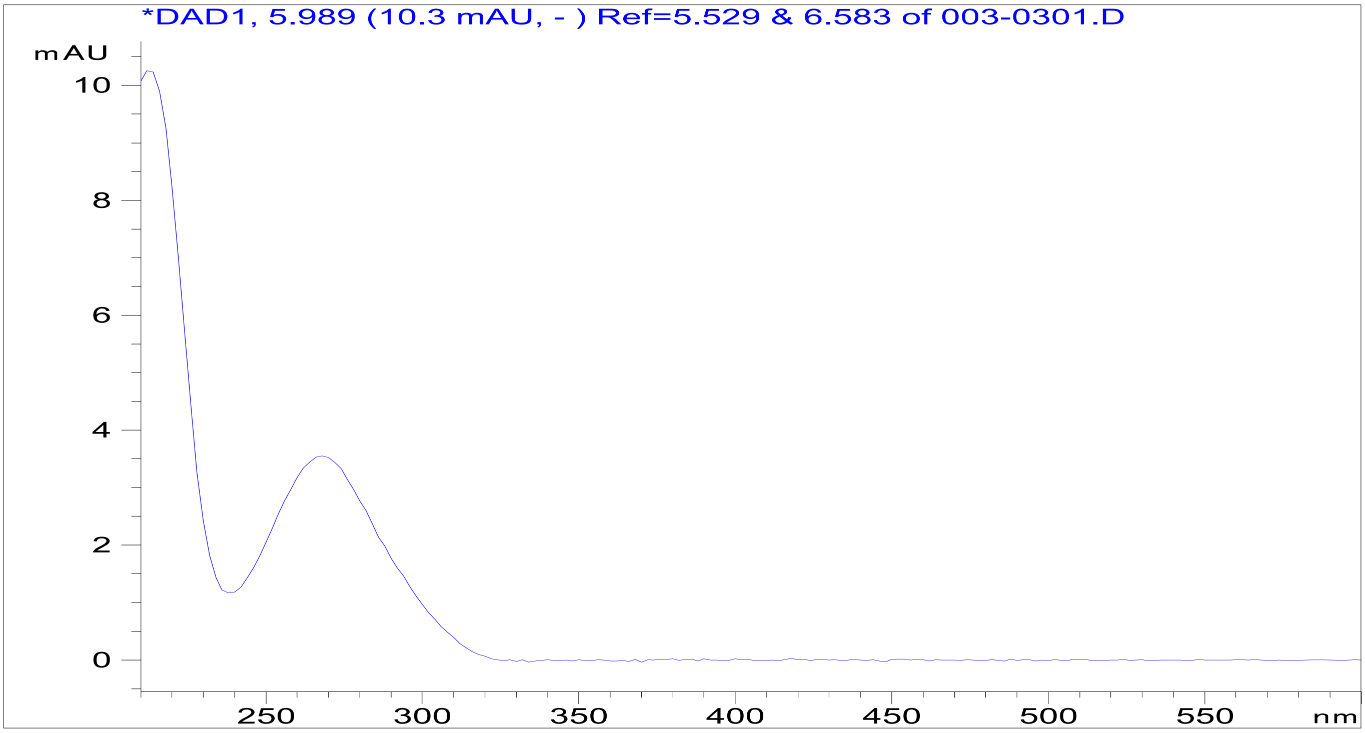

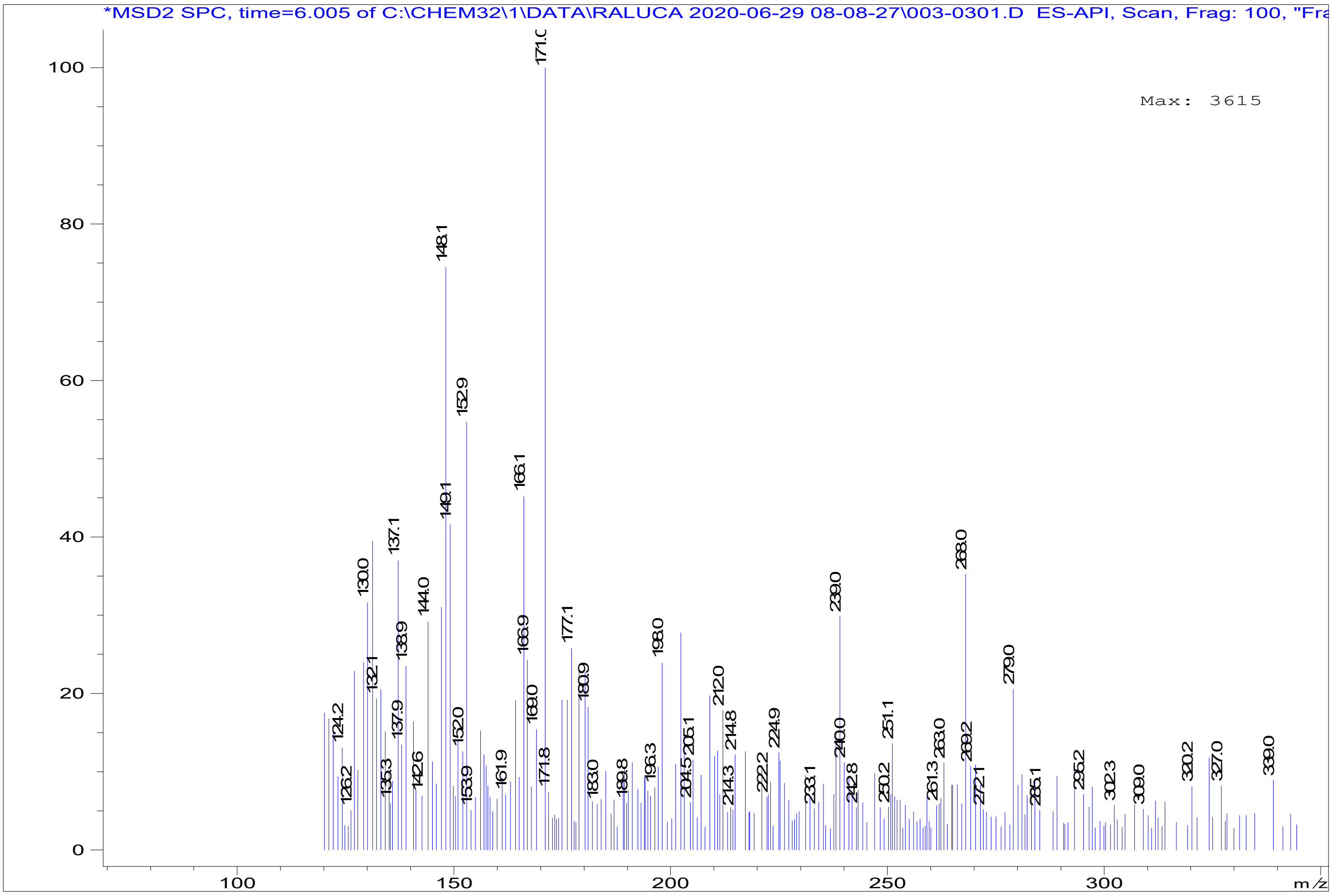

Rt=5.96 min Gallic acid

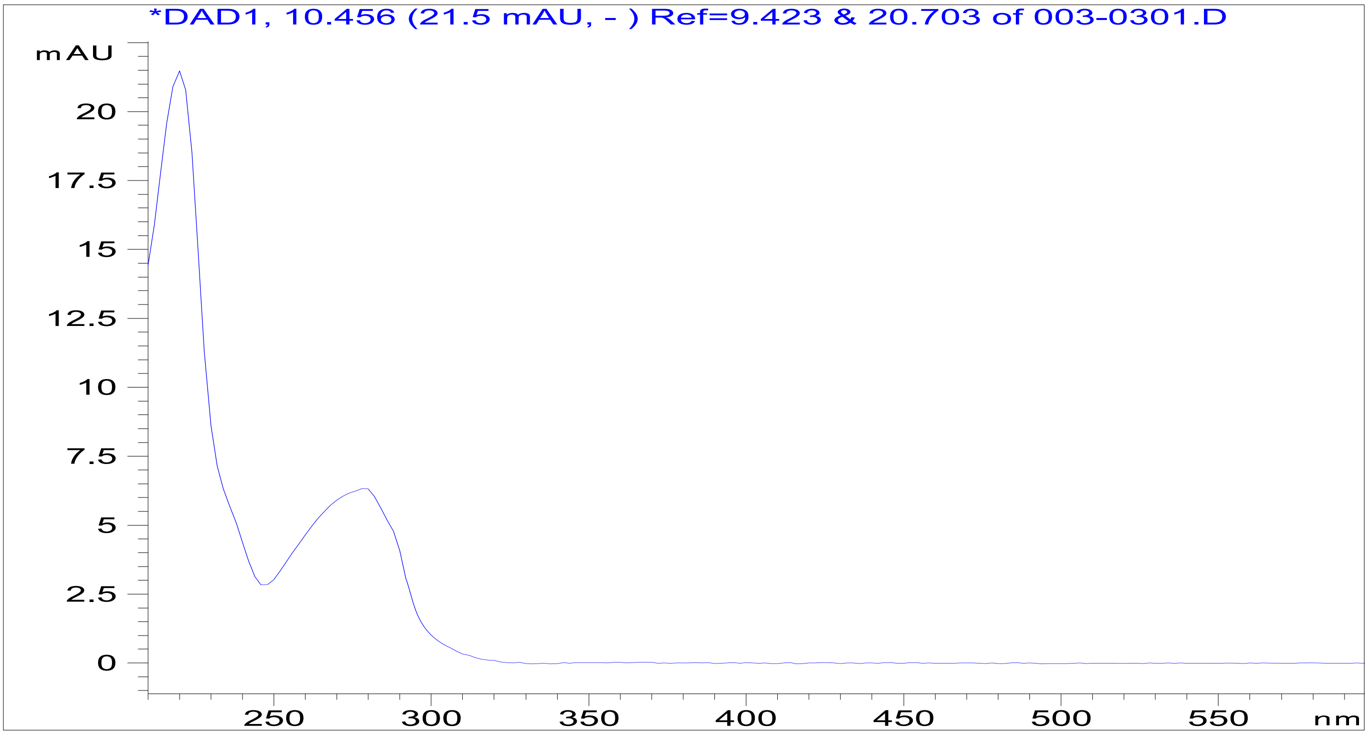

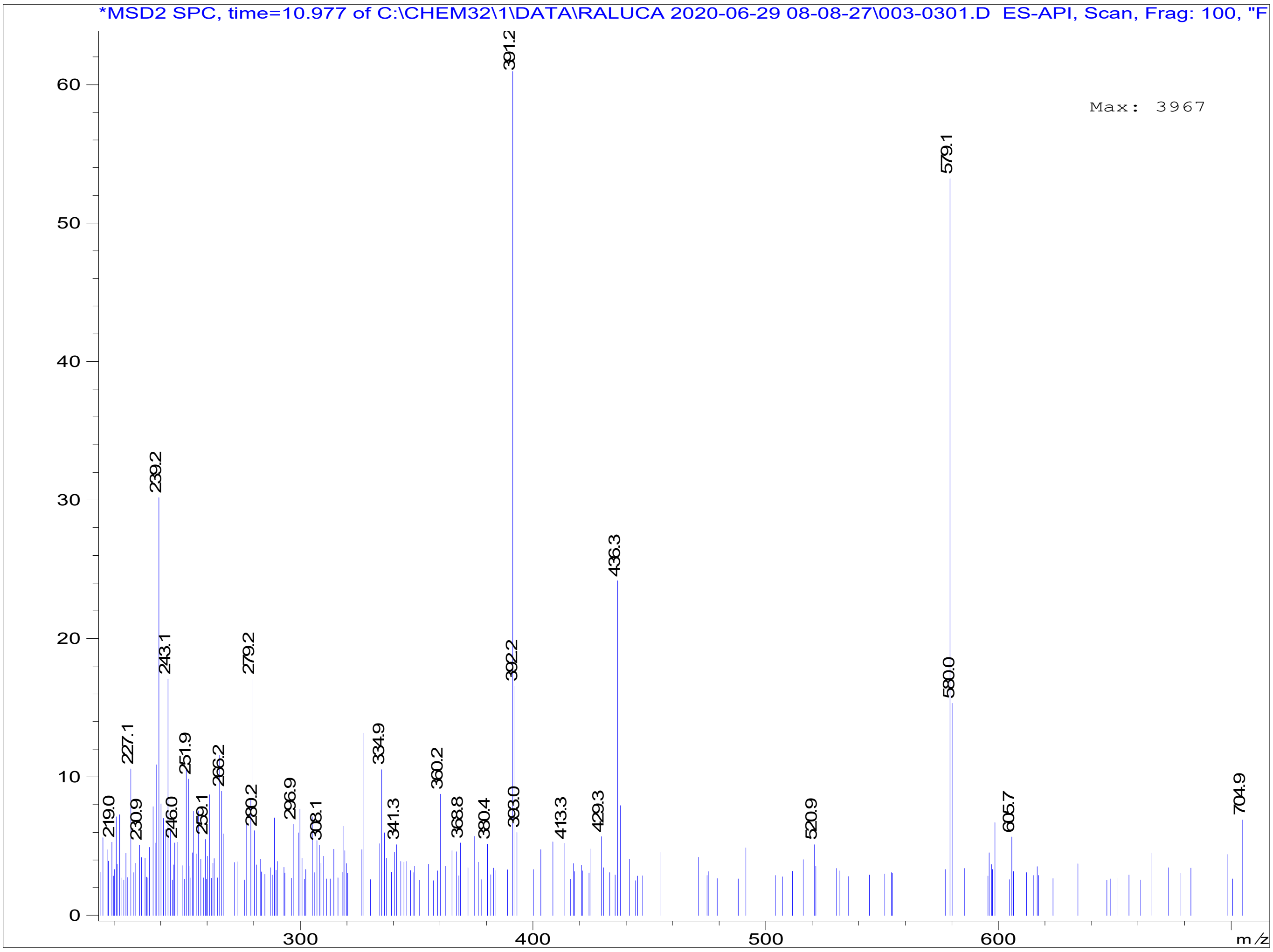

Rt=10.42 min Procyanidin dimer B1

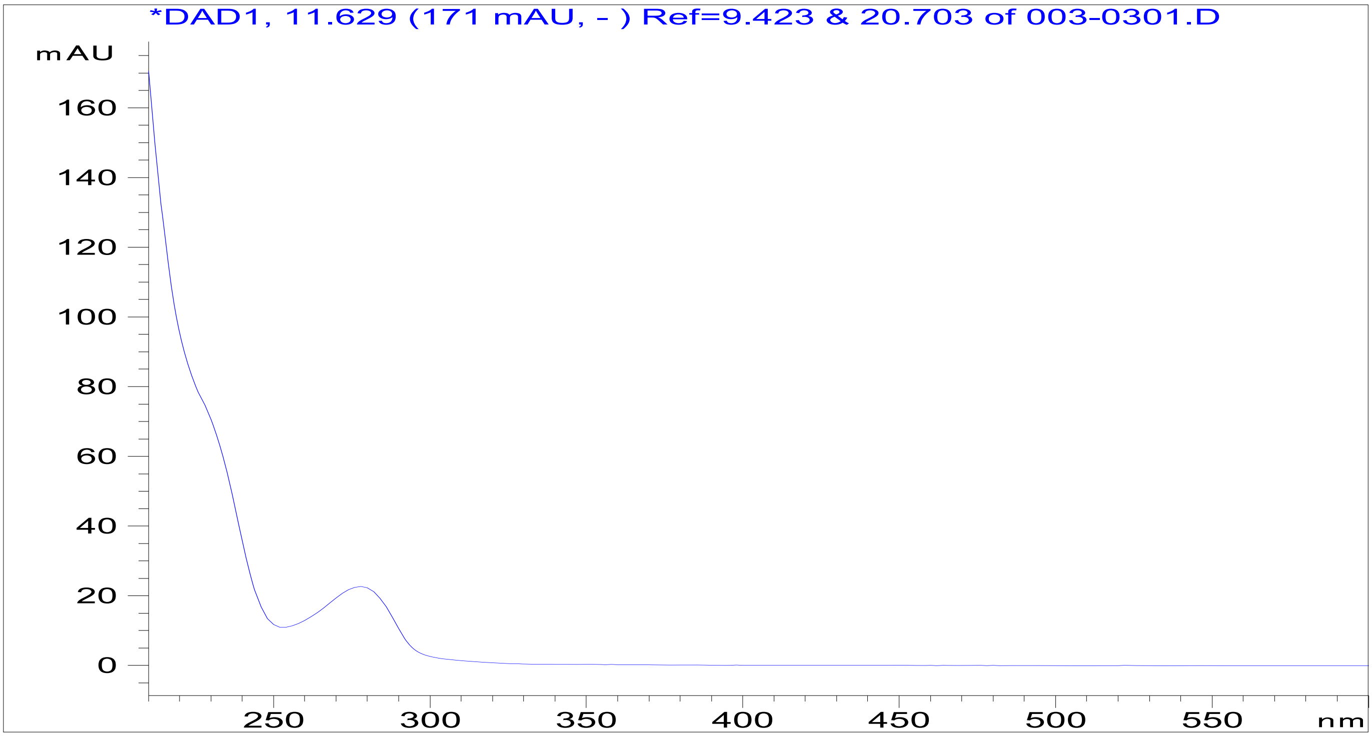

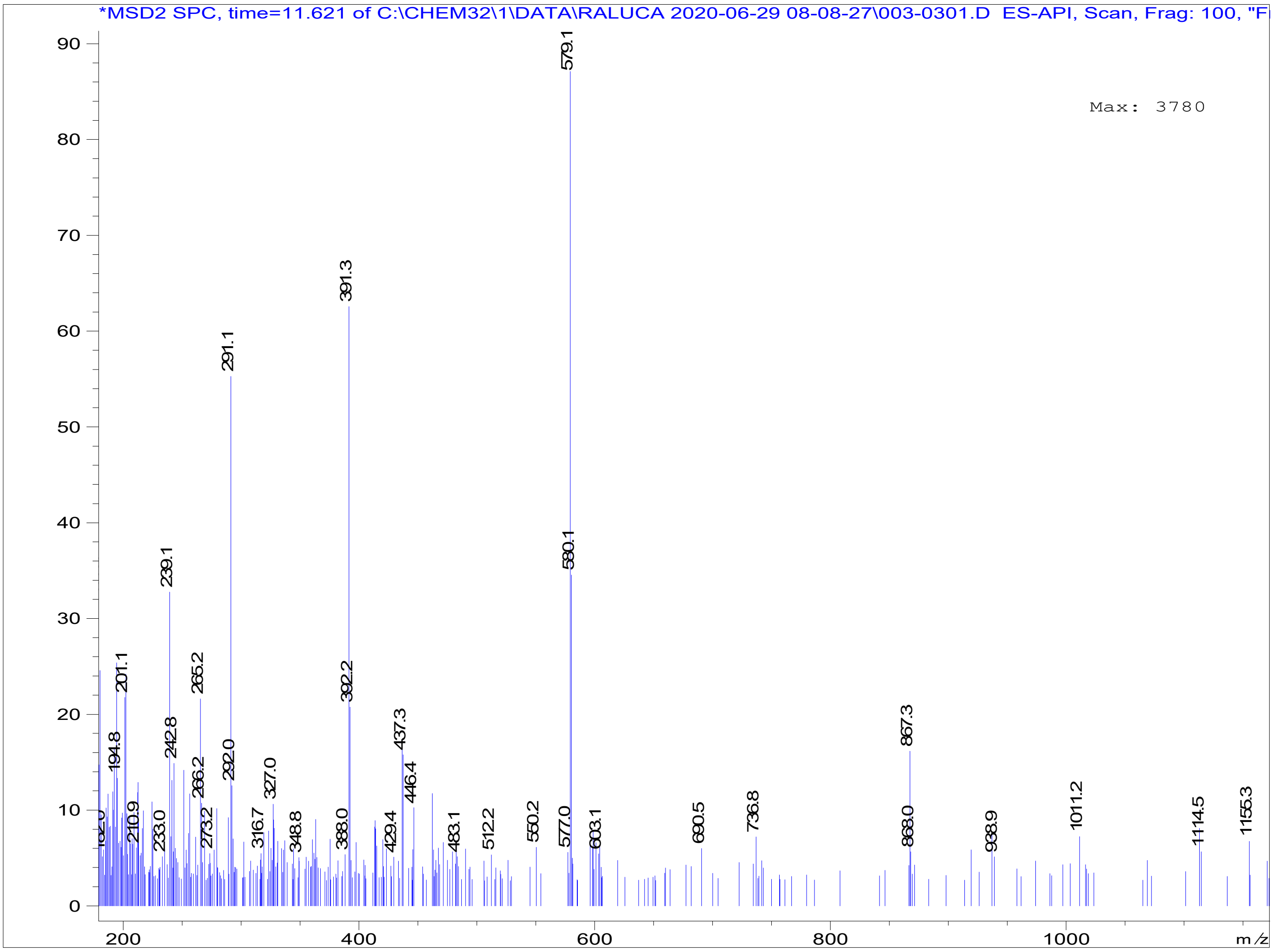

Rt=11.63 Procyanidin dimer B2

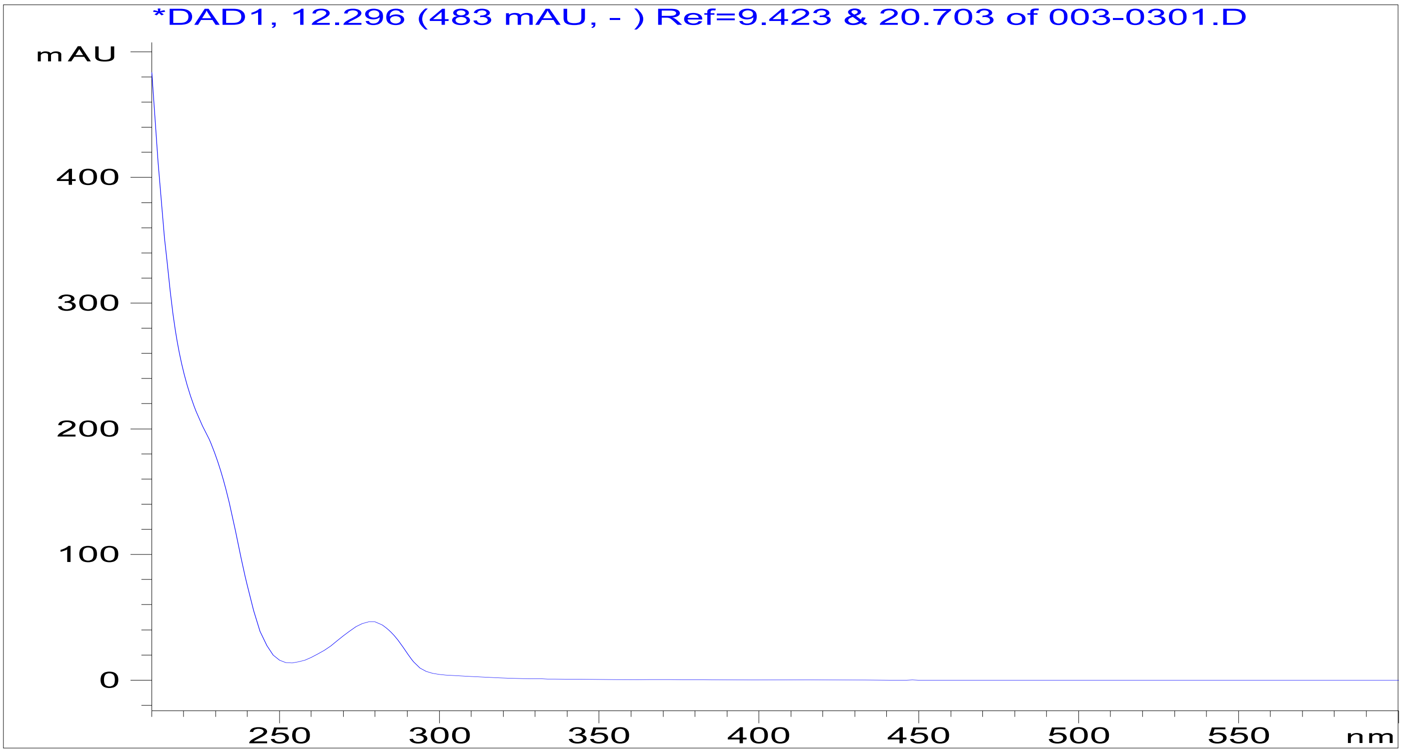

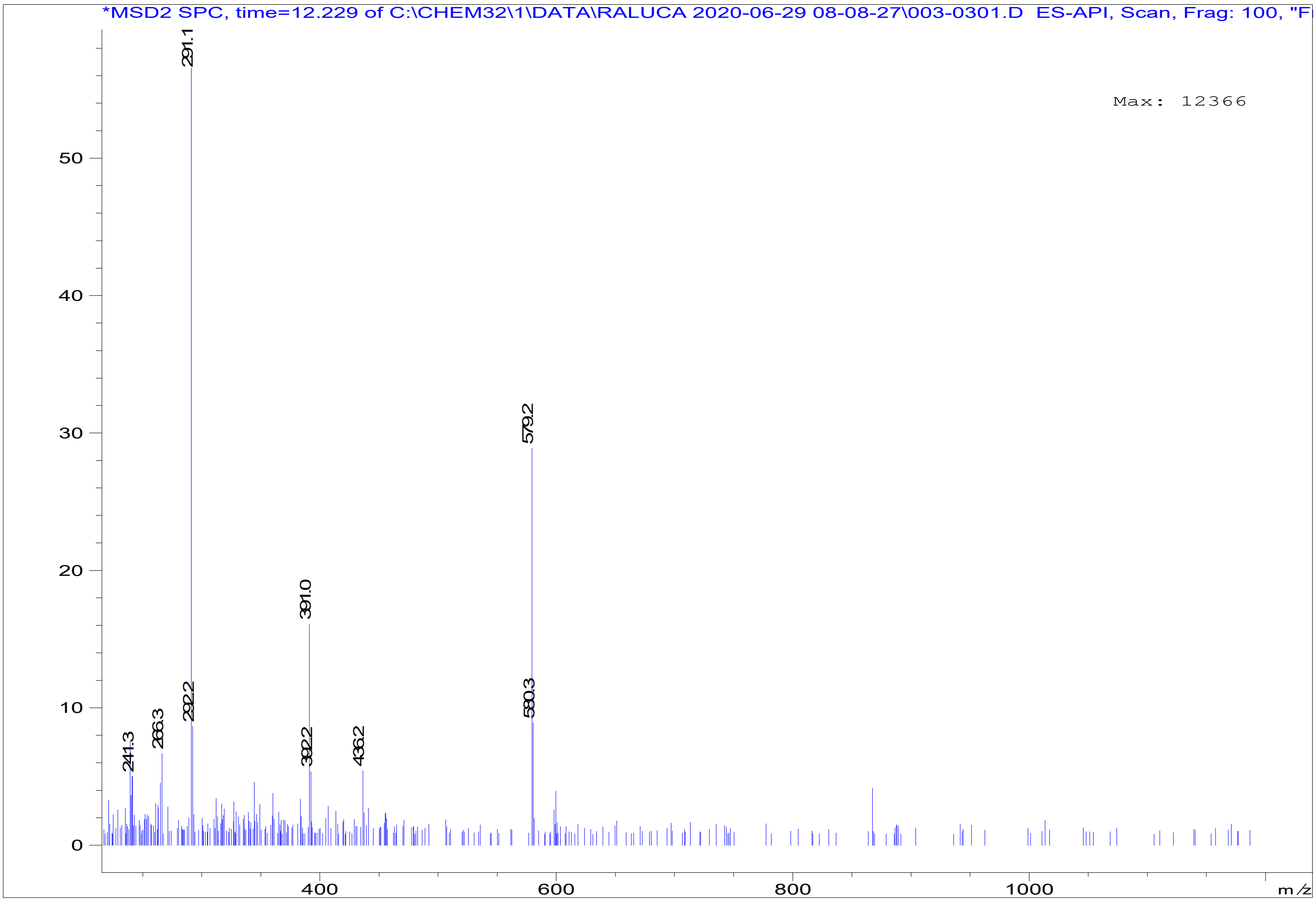

Rt=12.28 min Procyanidin dimer B3

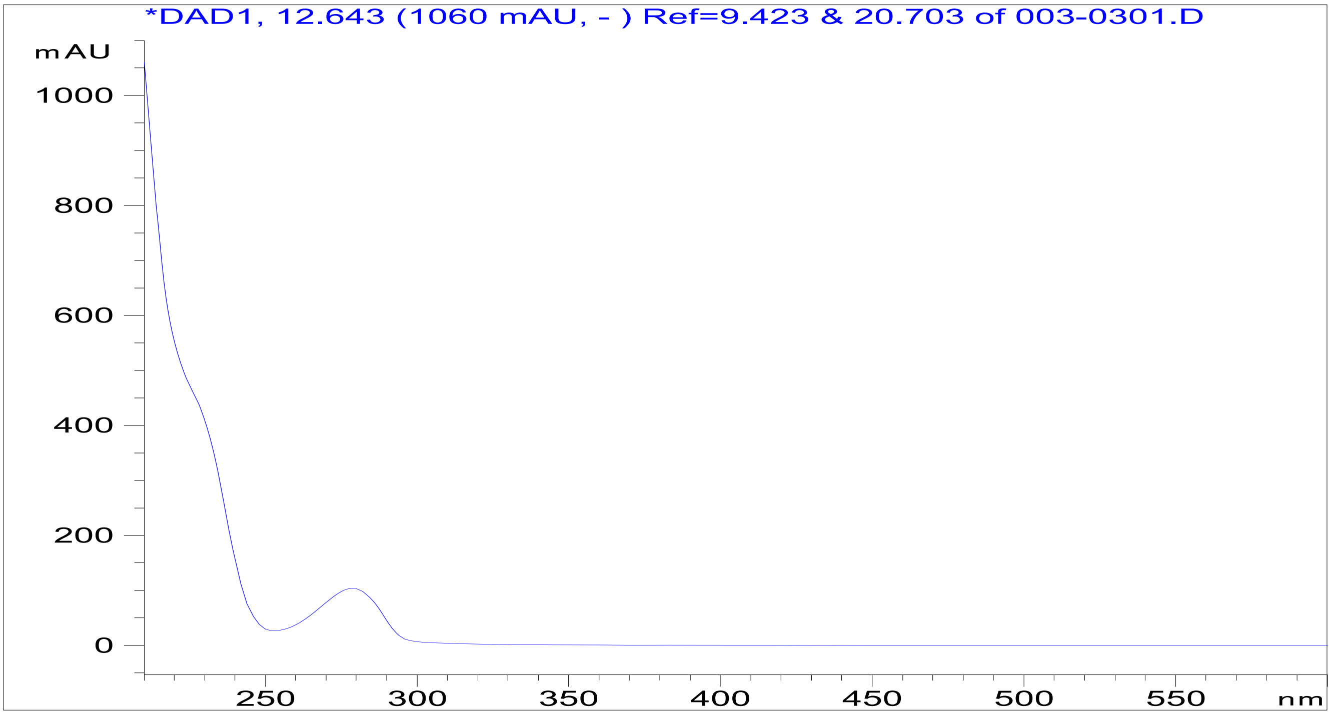

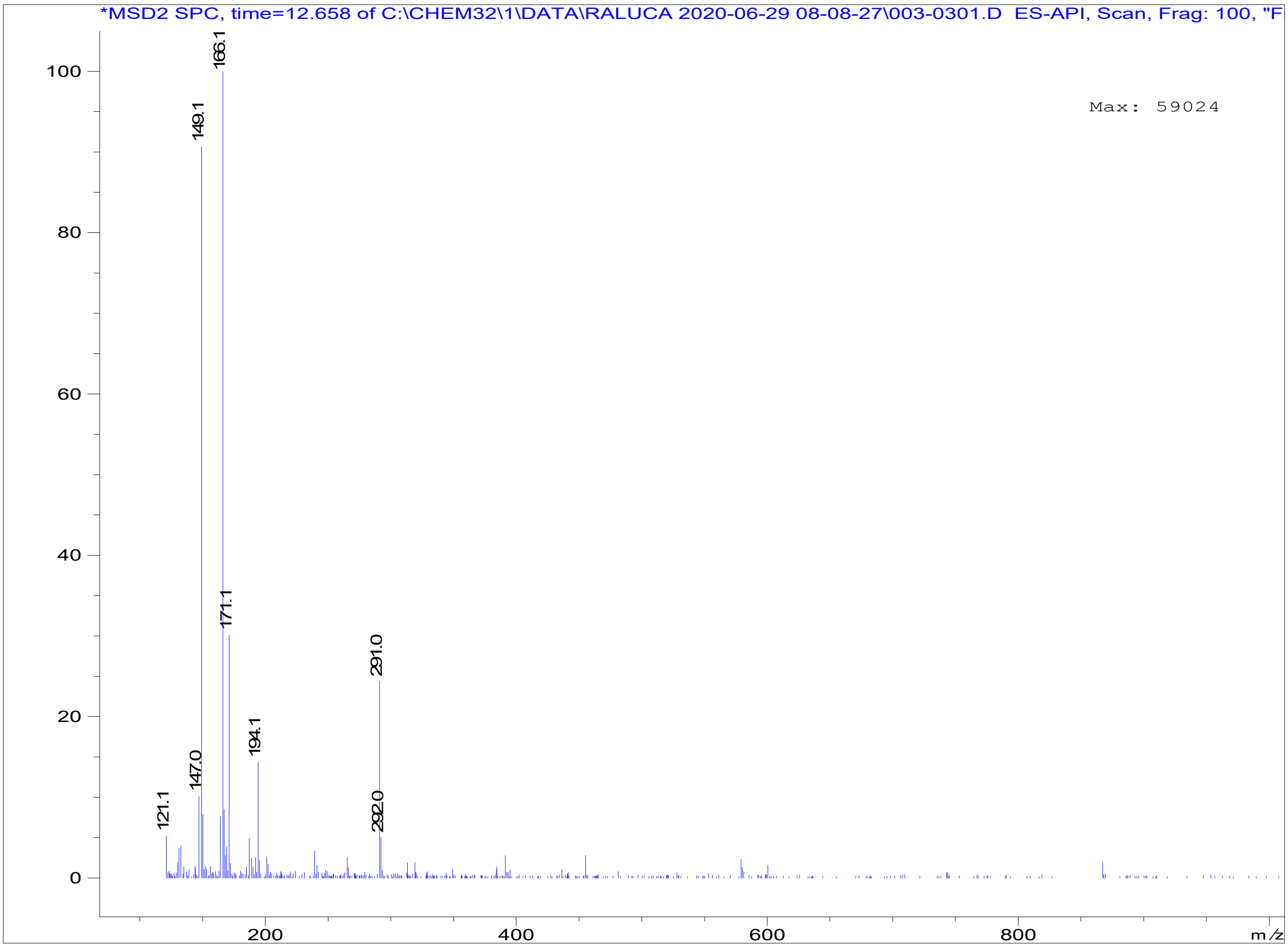

Rt=12.61 min Catechin

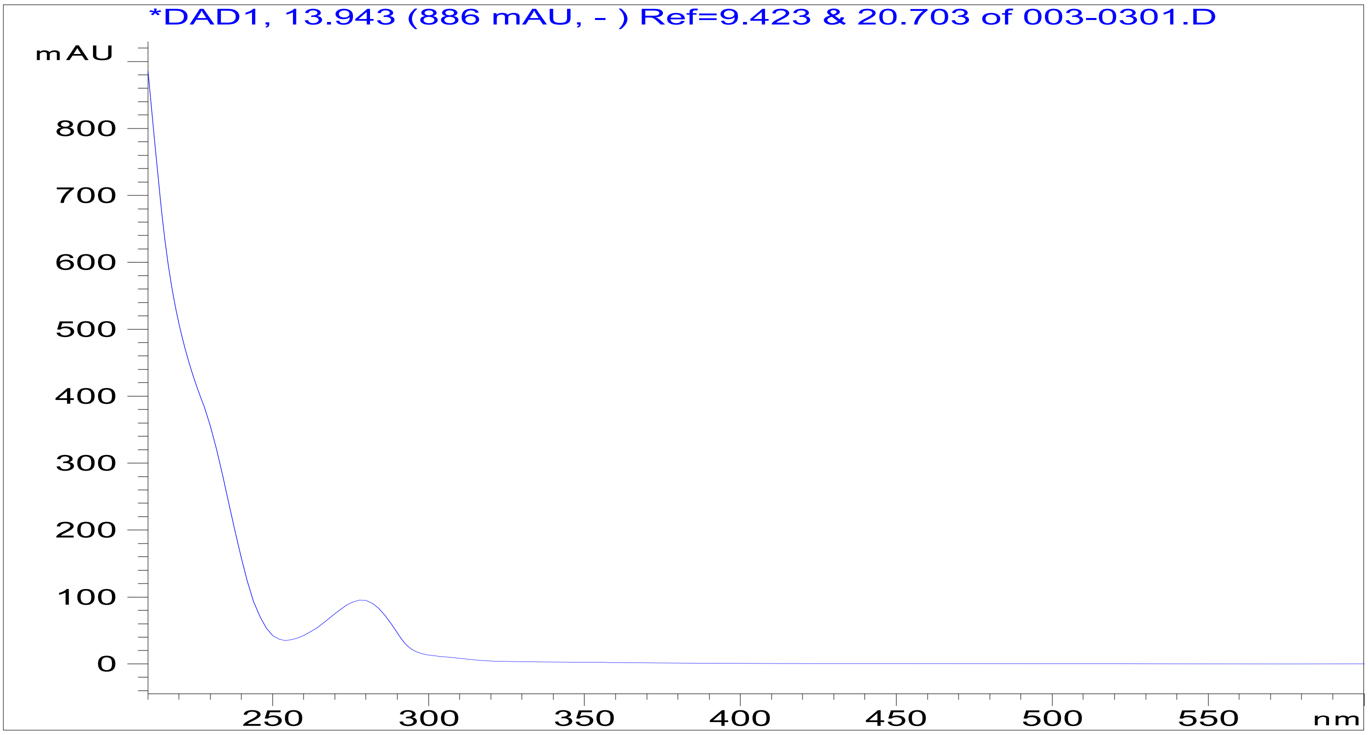

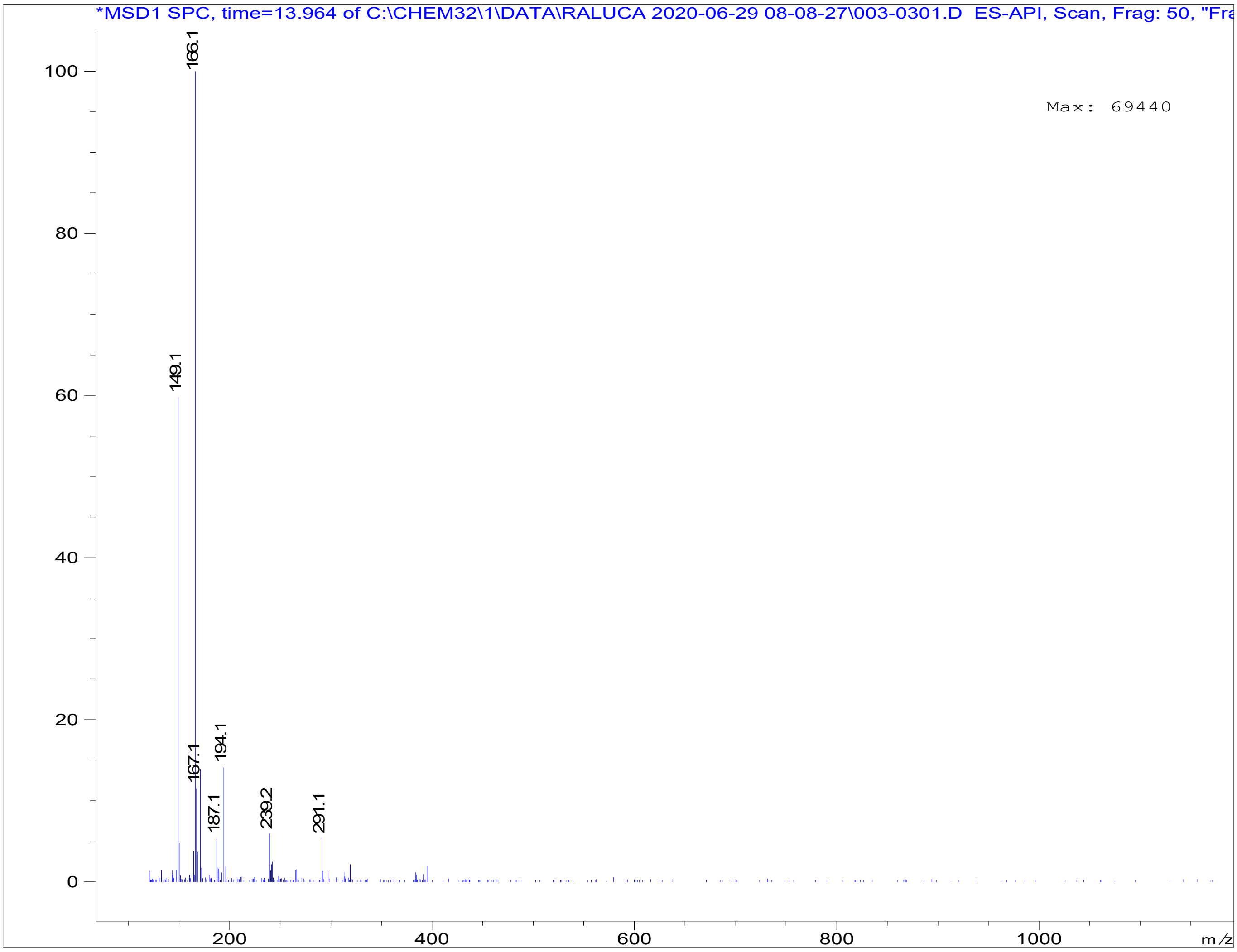

Rt=13.94 min Epicatechin

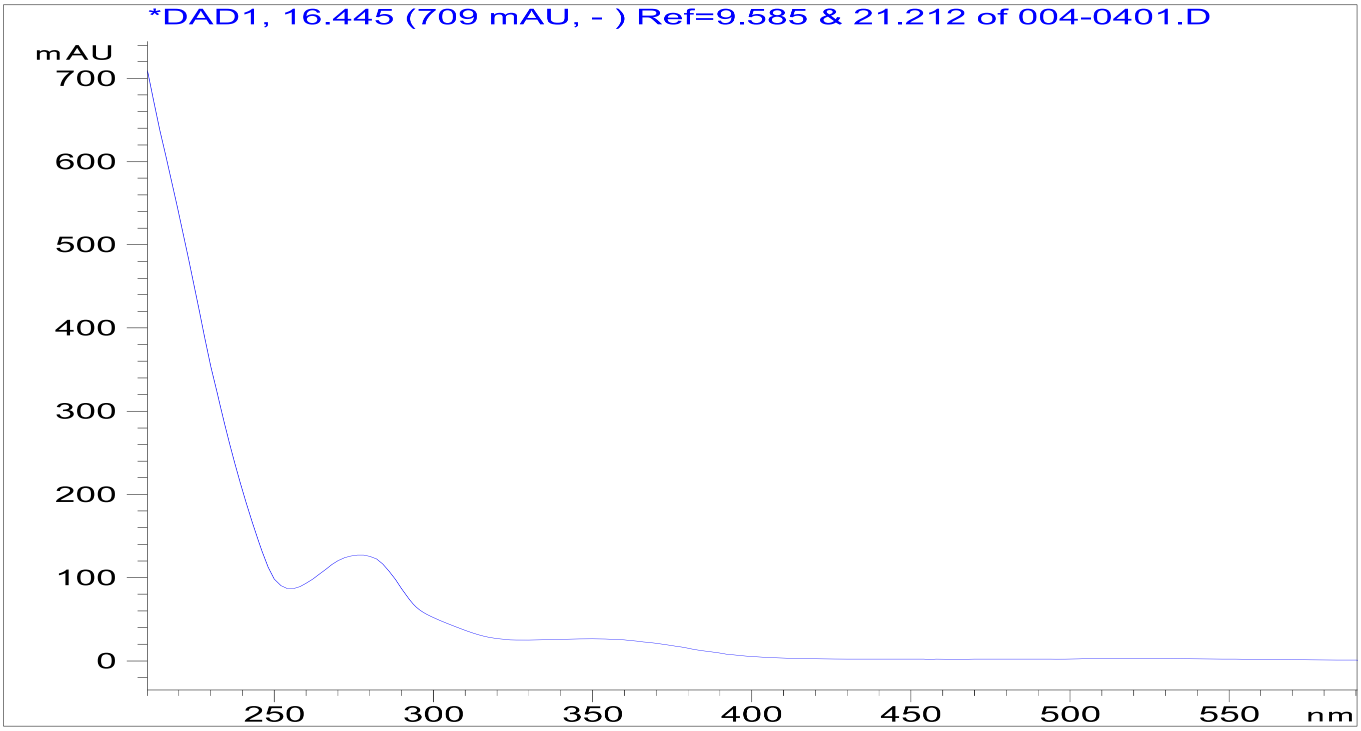

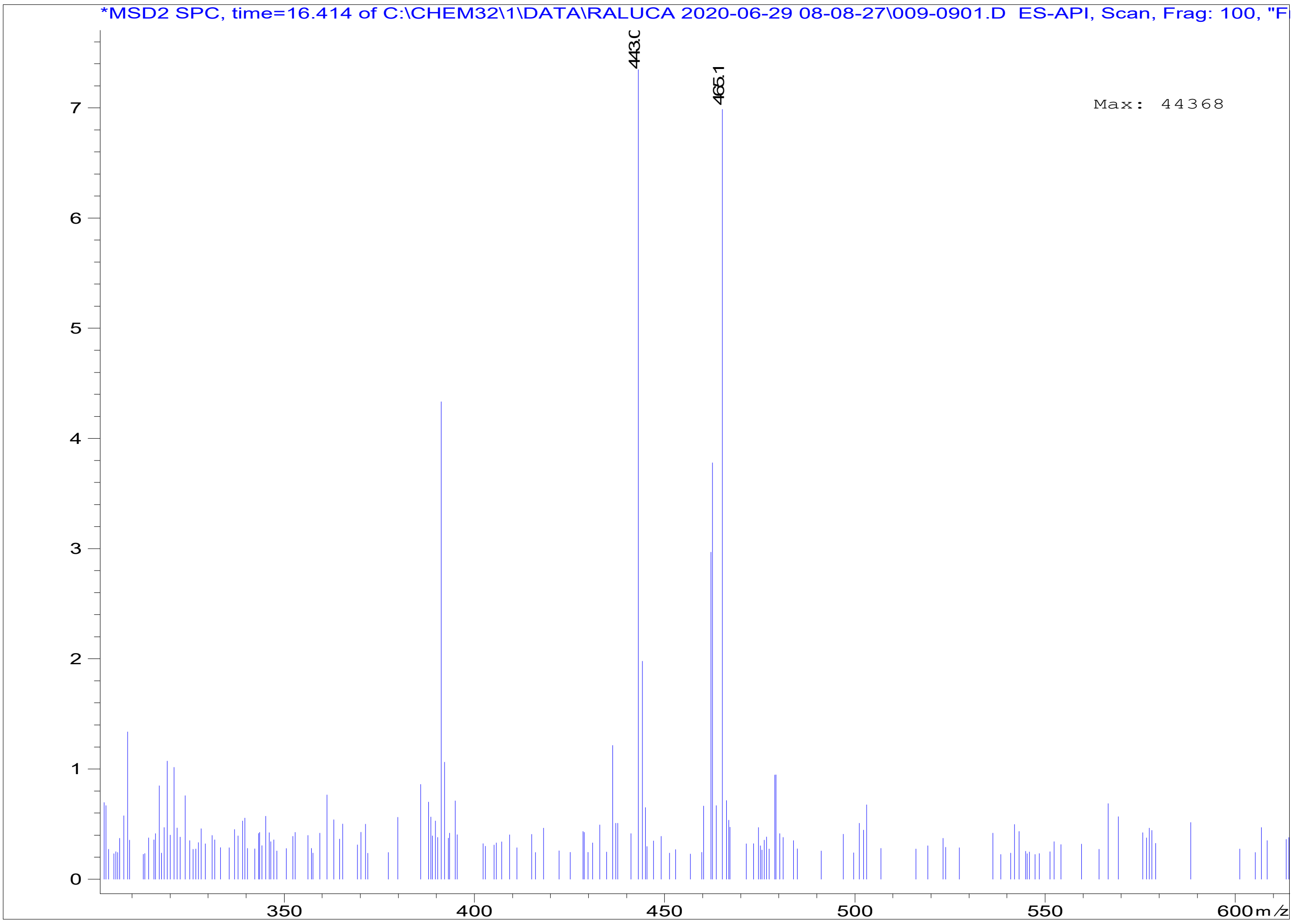

Rt=16.40 min Epicatechingallate

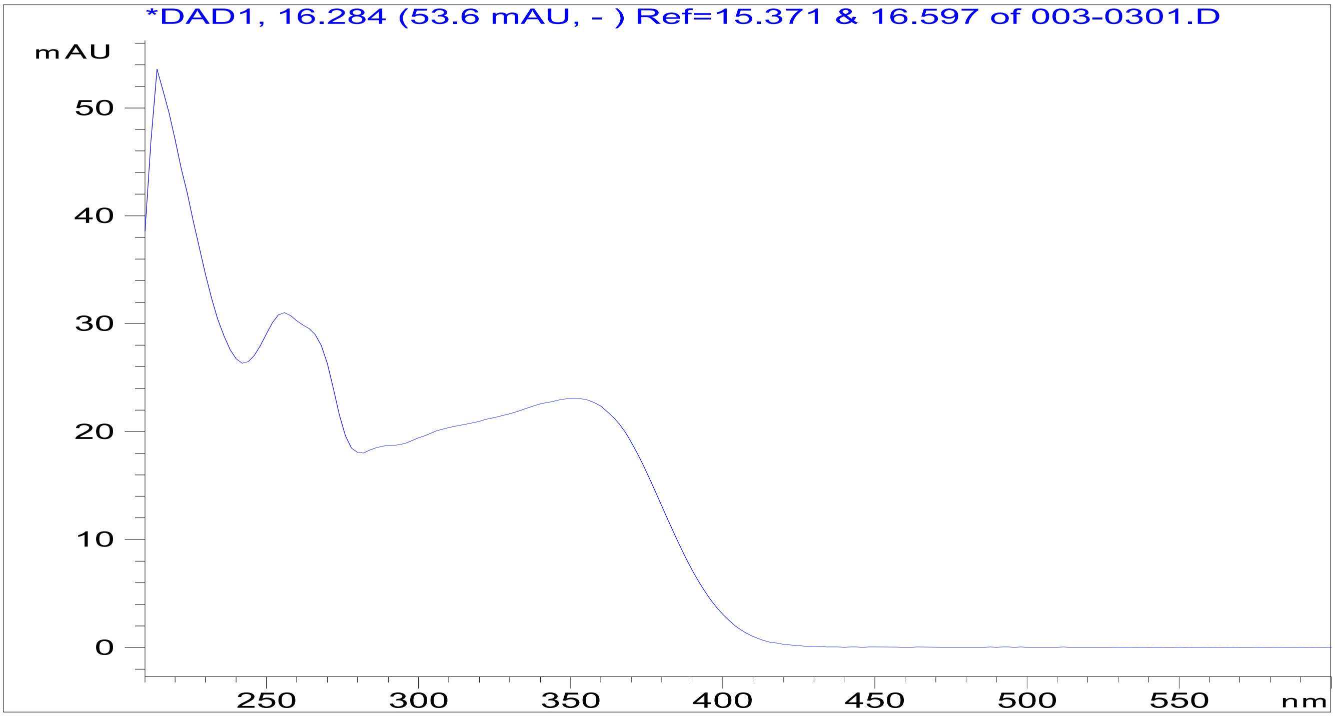

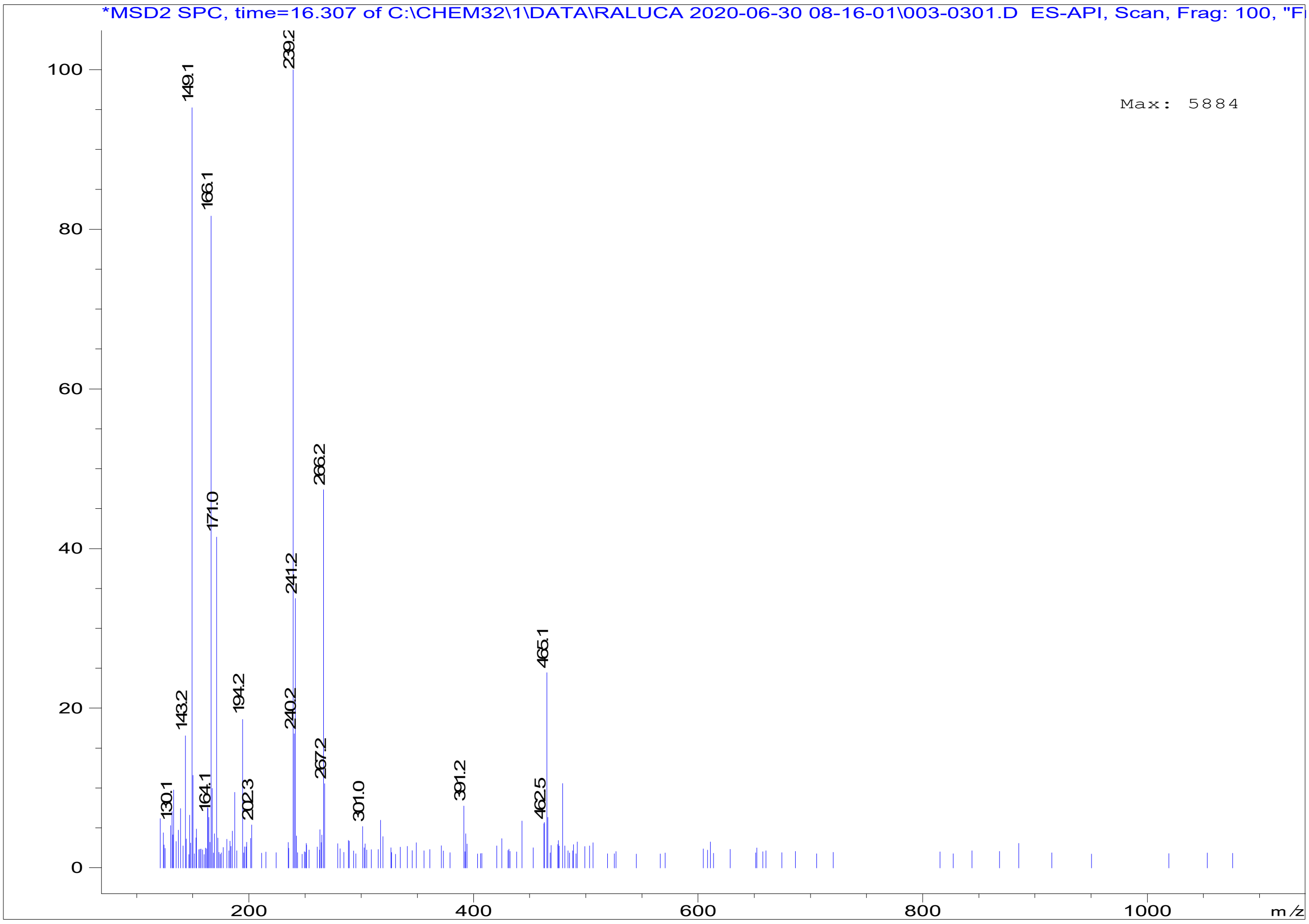

Rt=16.23 min Quercetin-glucoside

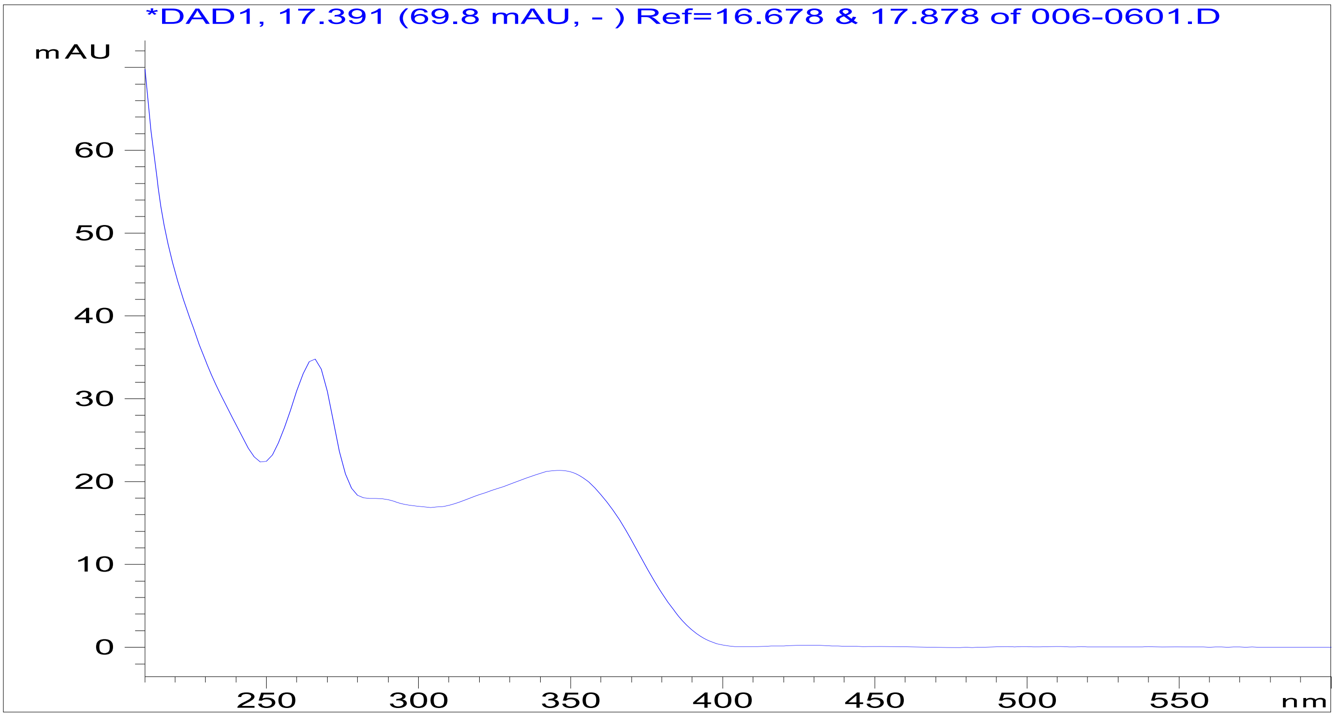

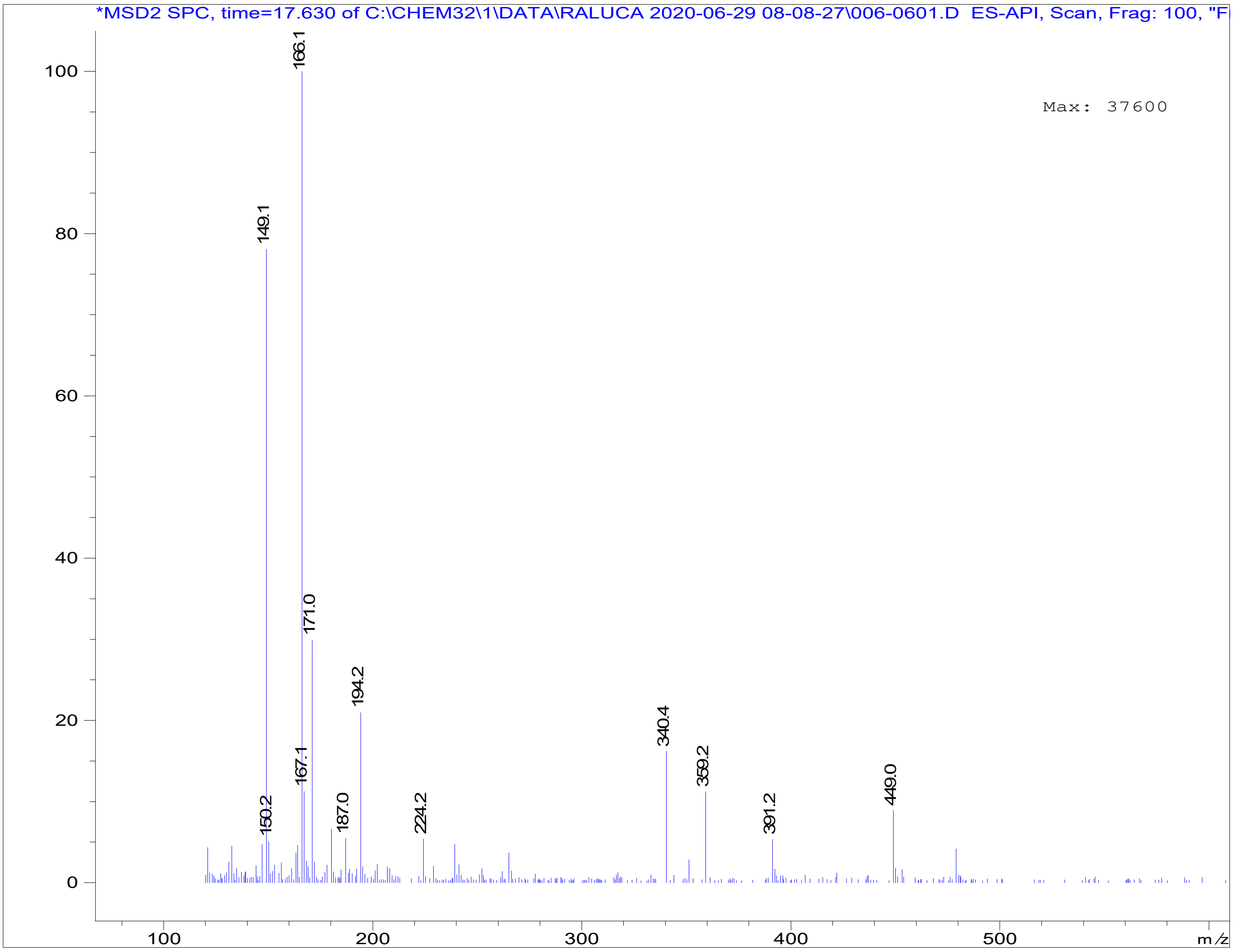

Rt=17.33 min Kaempferol-glucoside

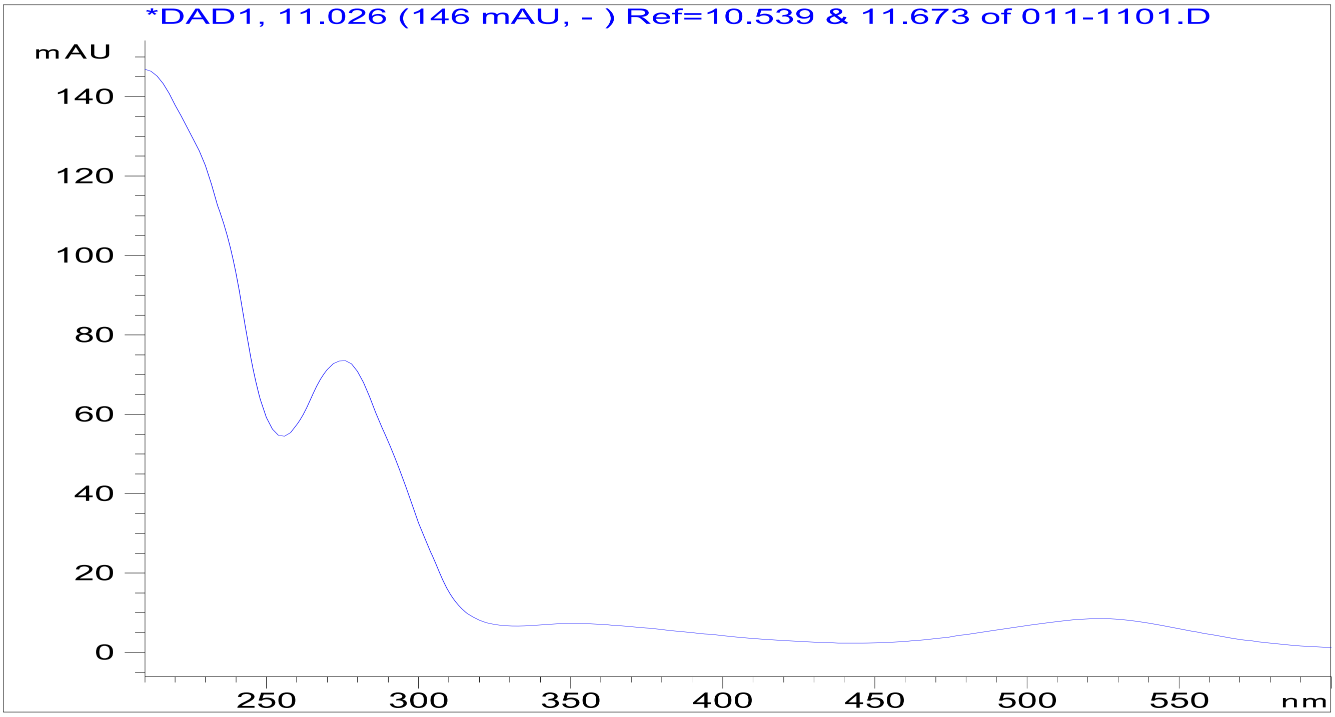

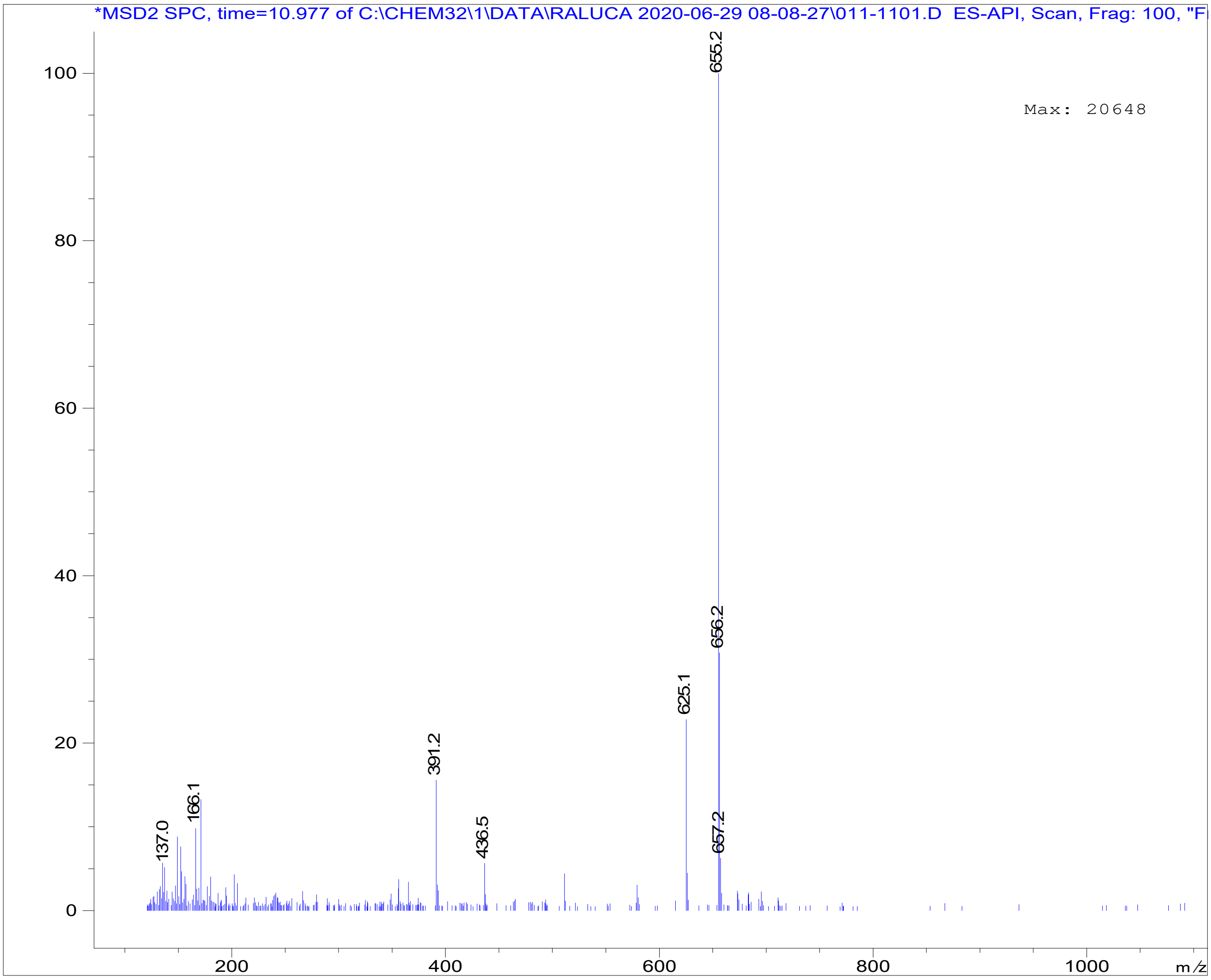

Rt=11.00 min Malvidn-caffeoyl-glucoside

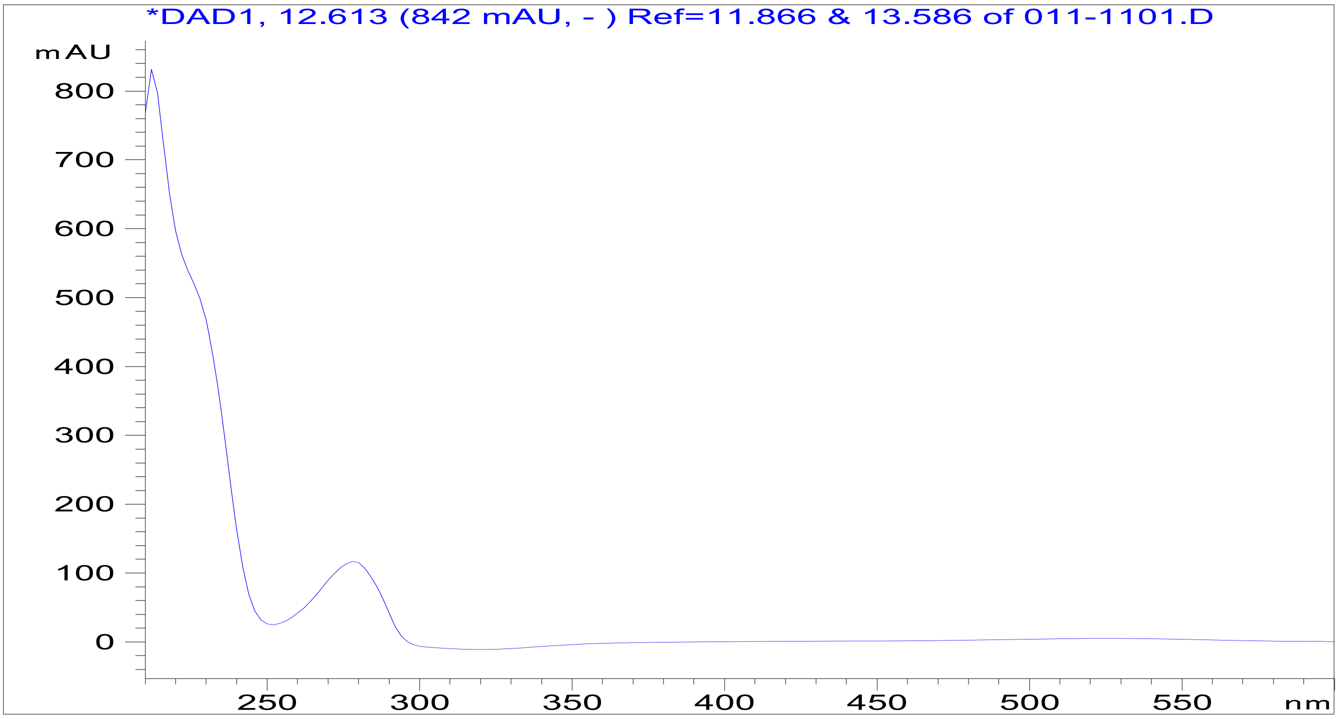

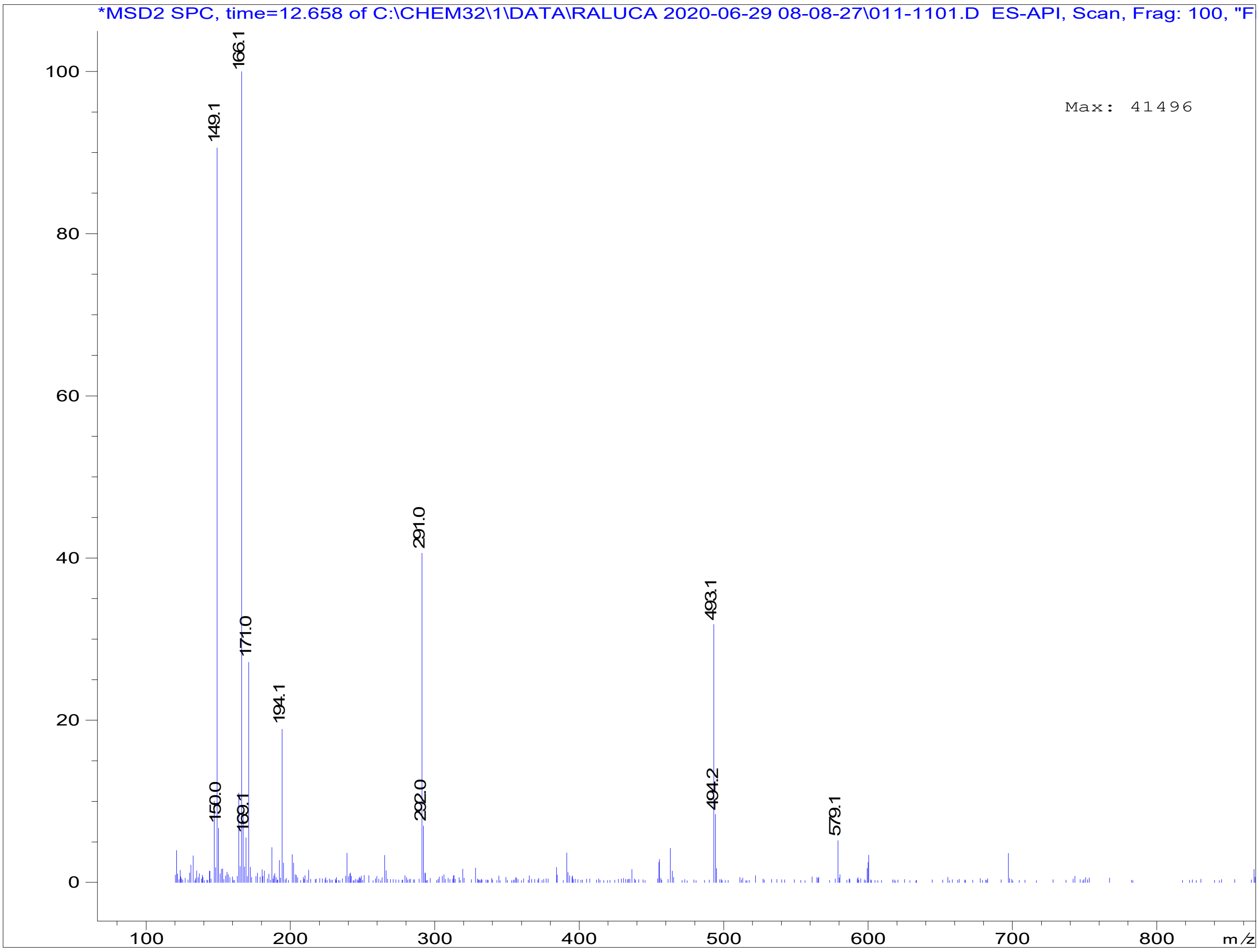

Rt=12.57 min Mlavidin-glucoside

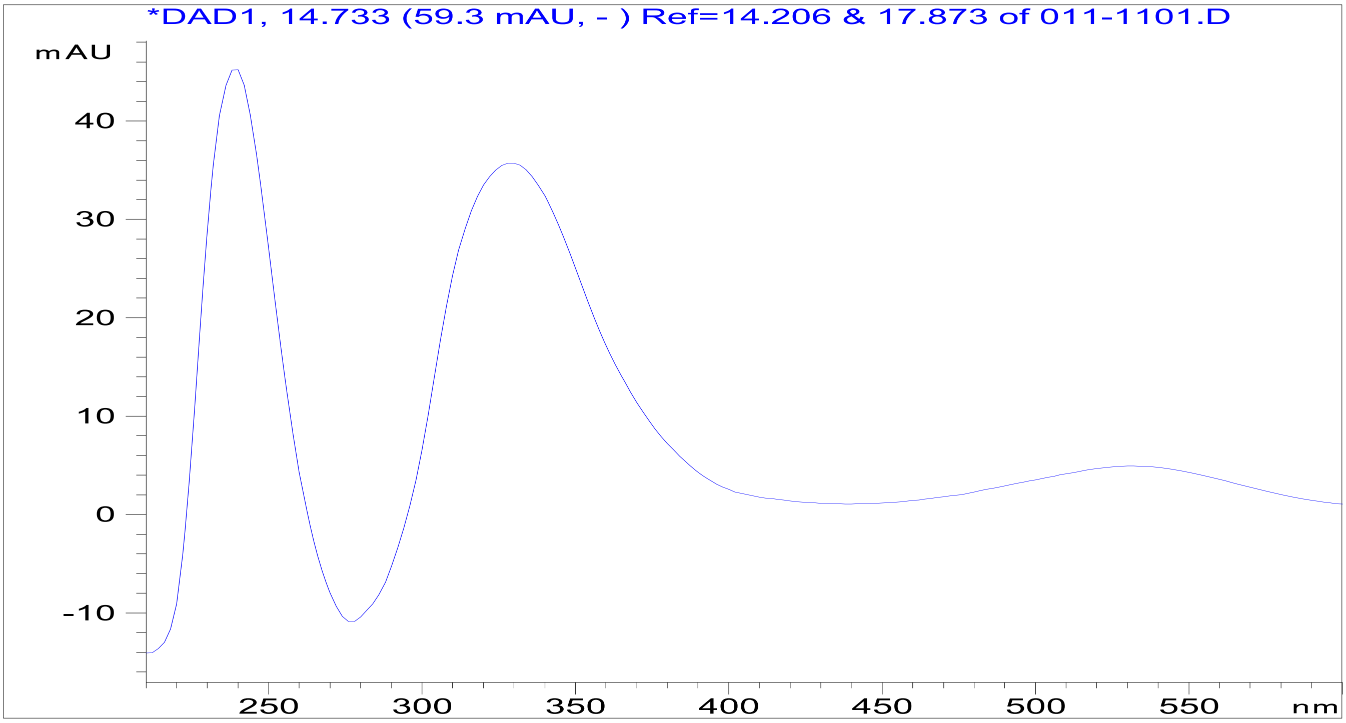

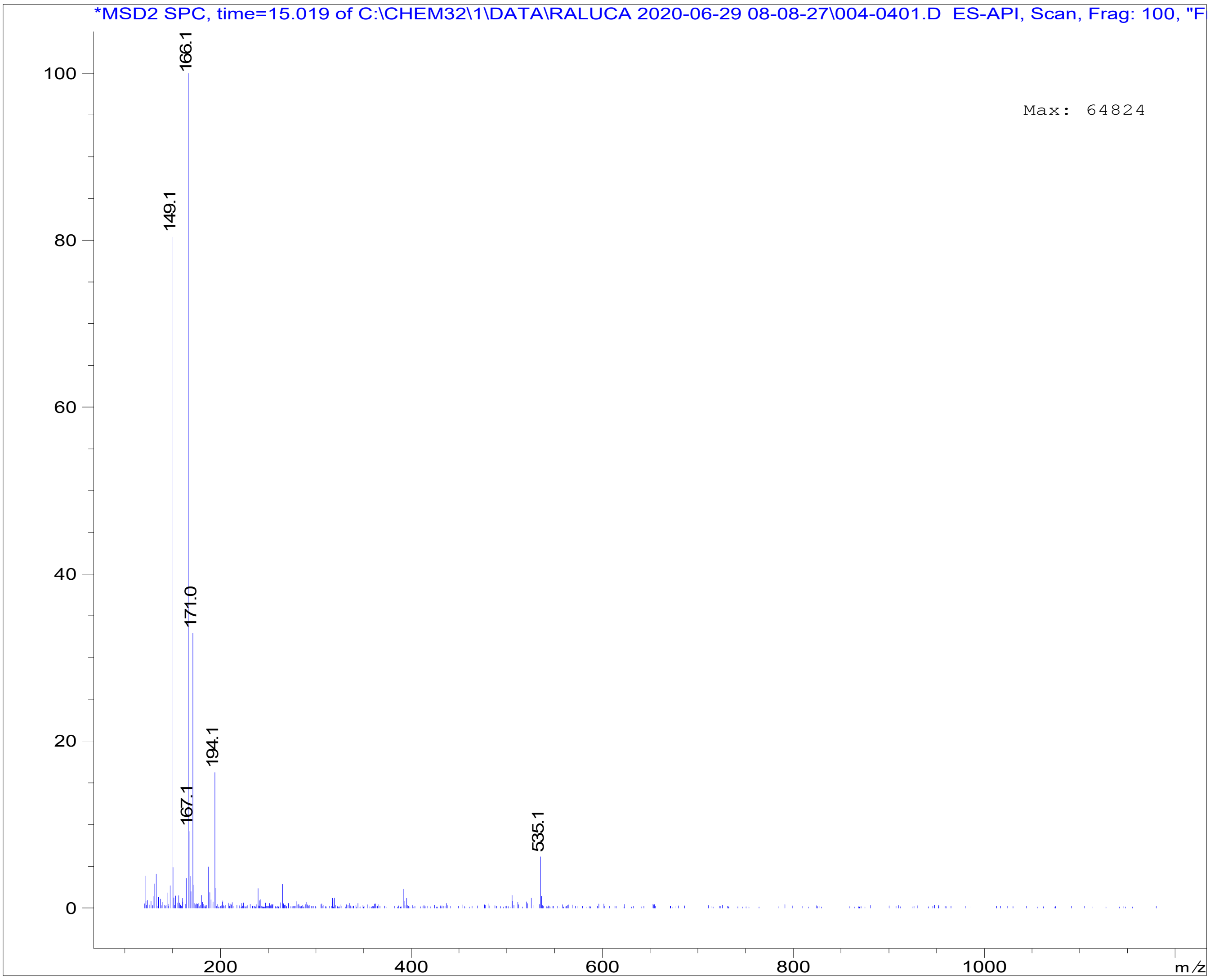

Rt=14.70 min Malvidin-acetyl-glucoside

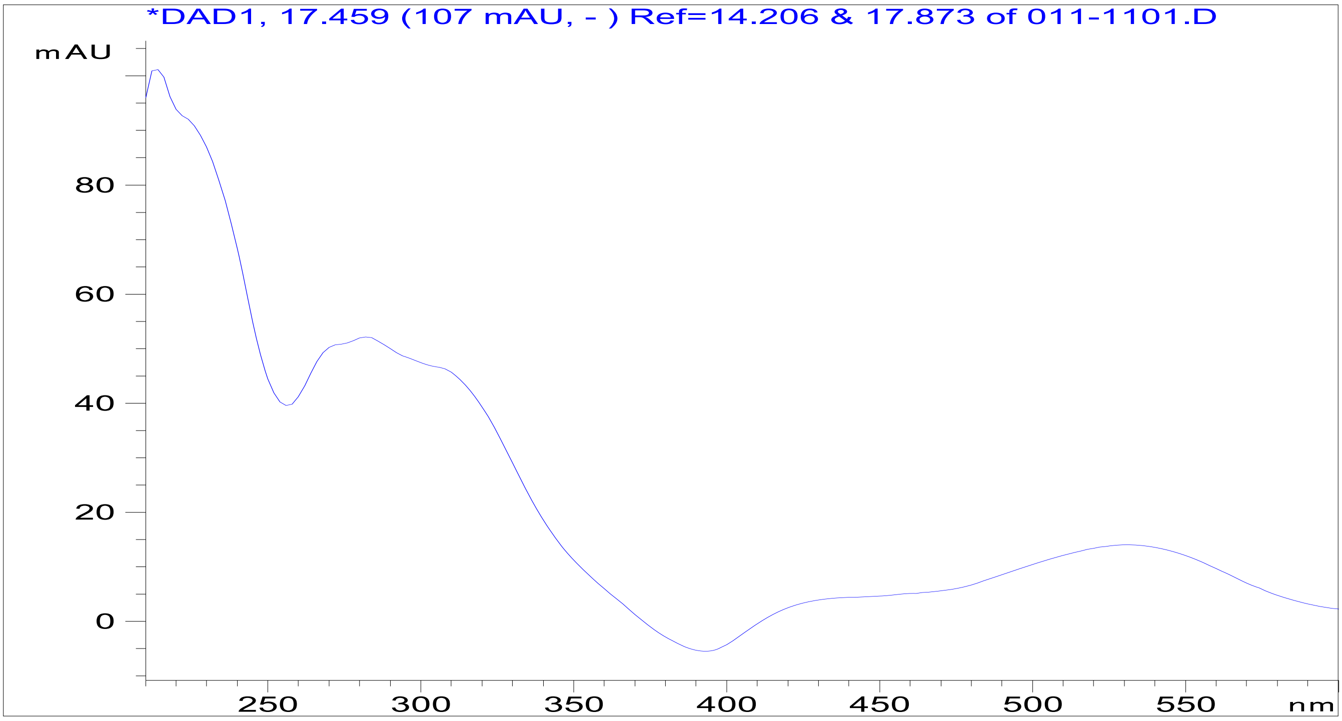

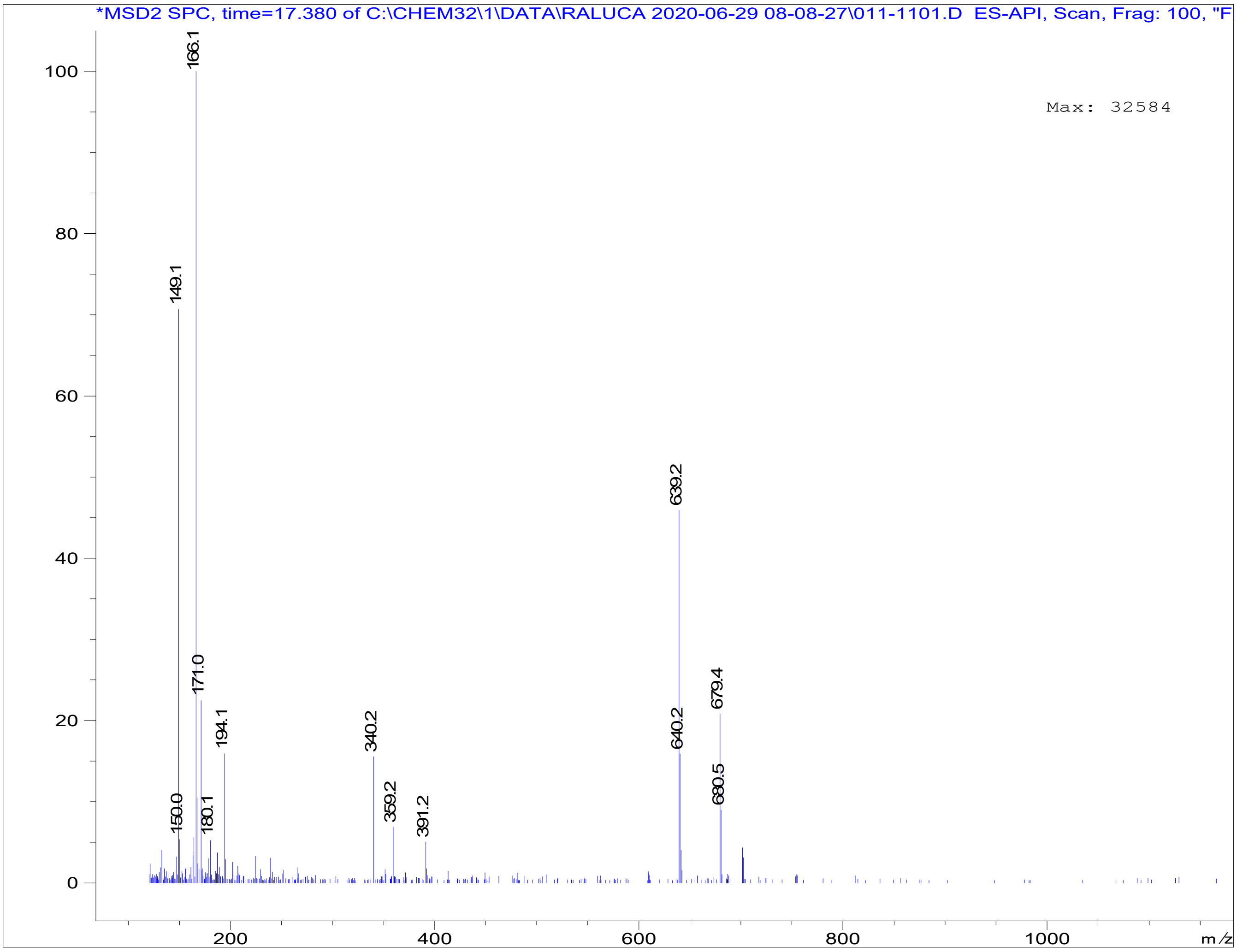

Rt=17.47 min Malvidin-coumaroyl-glucoside

Figure S3. UV-Vis DAD and MS after positive ionization spectra of the identified phenolic compounds

Table S2. Flavanol (µg/mL catechin equivalents) and hydroxybenzoic acid (µg/mL gallic acid equivalents) compounds determined in the grape pomace extracts by LC-MS

Compounds concentration (µg/mL) / Samples	RT (min); $[M+H]^+$ (*m/z*); λ_{max} (nm)										
	Hidroxy-benzoic acid 3.29 (min); 138 (*m/z*); 270 (nm)	Gallic acid 5.96 (min); 171 (*m/z*); 279 (nm)	Total phenolic acids	Procyanidin dimer B1 10.42 (min); 579, 291 (*m/z*); 280 (nm)	Procyanidin dimer B2 11.63 (min); 579, 291 (*m/z*); 280 (nm)	Procyanidin dimer B3 12.28 (min); 579, 291 (*m/z*); 280 (nm)	Catechin 12.61 (min); 291 (*m/z*); 280 (nm)	Epicatechin 13.95 (min); 291 (*m/z*); 280 (nm)	Epicatechin-gallate 16.40 (min); 443 (*m/z*); 280 (nm)	Catechin derivate 22.91 (min); - (*m/z*); 280 (nm)	Total Flavanols
BL	119.1 (118.6-119.6)	14.9 (14.6-14.9)[g]	134 (133.2-134.5)	31.3 (31.2-31.6)	40.4 (40.2-40.6)	37.8 (37.5-38)	56.8 (56.3-56.8)[g]	104.6 (103-105.6)	55.1 (54.7-55.5)	108.3 (107.5-109.3)[g]	434 (431-436.9)
RR	84.3 (82.7-85.8)	14.2 (14.1-14.6)	98.3 (97.1-99.9)[g]	24.2 (24.1-24.6)	30.9 (30.5-31.1)	25.6 (25.3-25.7)	61.1 (61-62)	90.3 (88.6-92.3)	22.1 (22-22.5)[k]	117 (114.6-120.6)	371.4 (366.8-378.6)[g]
RB	93.2 (91.5-95.1)	14.9 (14.4-14.9)	108.1 (105.9-110.1)[g]	24.5 (24.1-24.7)	11.9 (11.8-11.9)[a,h,o]	23.7 (23.6-23.8)	79 (78.1-80.3)	92 (90.1-94.1)	52.2 (50.3-54)	129.5 (124.7-134.5)	413 (403.2-423.4)
AS	106.7 (103.5-109.5)	15.7 (15.6-15.8)	122.5 (119.1-125.3)	21.8 (21.7-21.9)	13.5 (13.3-13.7)[o]	15.7 (15.4-15.8)[p]	108.3 (105.7-110.8)	106.3 (103.7-108.7)	66 (64.8-67.6)	86.8 (84.5-88.5)[g,o]	418.6 (416.4-420.3)
TR	81.4 (78.8-84.7)	21.3 (21.1-21.6)	102.8 (99.9-106.3)	24.4 (24.2-24.6)	19.9 (19.5-19.9)	26.4 (26.2-26.7)	148.9 (145.9-150.5)	116.1 (111.6-120.8)	28.7 (28.2-28.8)	246.1 (242.1-250.5)	610.9 (603.5-616.7)
JO	113.6 (111.9-115.8)	41.9 (41.1-42.5)	155.5 (153-158.4)	19.7 (19.6-19.8)	28.8 (28.8-29)	39 (38.8-39.4)	112.8 (110.7-114.9)	135.1 (133.3-137.2)	57.6 (56.4-58.3)	282.2 (279.2-285.7)	675.8 (667.4-684.4)
NE	144.6 (142.6-146.6)	59.1 (58.1-59.9)	203.7 (200.8-206.5)	27.9 (27.8-28.1)	24.1 (24-24.3)	37.3 (37.1-37.5)	217 (213.9-221)	279.2 (274.2-284.2)	35.8 (35.7-35.8)	398 (393-404.1)	1019.4 (1013.8-1027.4)
RU	87.6 (85.9-88.8)	15.9 (15.7-15.9)	103.6 (101.9-104.5)	21.5 (21.4-21.6)	40.1 (39.2-41.4)	26.5 (26-27.1)	73.9 (72.1-75.3)	96.1 (94.7-97.7)	31.2 (30.6-32.2)	176.3 (173.8-179)	466 (458.1-474.5)
SB	82.3 (81.3-83.8)	18.4 (18.2-18.7)	100.8 (99.6-102.5)	40.8 (39.5-41.6)	22.2 (22-22.6)	16.3 (16.3-16.7)	80.3 (78.3-82.2)	90.5 (88.4-92.3)	36.6 (35.8-36.9)	264.7 (260.5-268.4)	551.7 (541-561.2)
FR	78.1 (76.6-79.6)[o]	19.9 (19.7-20.1)	97.9 (96.3-99.6)	37.3 (37.1-37.4)	33.4 (33.2-33.7)	22.5 (22.4-22.6)	72.5 (71.2-74)	125 (122.9-127.6)	22.1 (21.8-22.6)[k]	281.1 (277.2-285.5)	594.3 (586-603.7)
RA	86.1 (84.6-87.9)	19.9 (19.9-20.1)	106.1 (104.5-108.1)	18.4 (18.4-18.5)	30.3 (30.2-30.5)	24.6 (24.5-24.7)	205.9 (201.1-209)	230.3 (226-234.3)	236.2 (231.4-239.9)	190.1 (187-194.5)	936.2 (918.9-951.7)
BR	94.4 (92.2-96.1)	24.1 (24-24.2)	118.5 (116.3-120.3)	25.8 (25.7-26)	24.9 (24.8-25) 1	25.6 (25.5-25.7)	113.7 (111.7-115.5)	220.1 (216.6-223.6)	112 (110.9-114)	207.9 (204.7-210.8)	730.4 (720.2-740.8)

SE	85.4 (83.8-87.1)	15.1 (15-15.4)	100.5 (98.9-102.6)	17.7 (17.4-17.9)[i]	18 (17.9-18.4)	21.2 (21.1-21.5)	32.8 (32.5-33)[g,k]	58.9 (57.5-59.7)[g]	28.4 (28.2-28.7)	223.6 (220.1-227.4)	400.9 (395-406.8)
MO	51.2 (50.1-52.2)[g,o]	17.8 (17.5-17.9)	69.1 (67.6-70.1)[g,o]	17.4 (17.2-17.6)[i,j]	13.7 (13.5-13.8)	15.9 (15.8-16.1)[p]	70.5 (69.3-71.6)	55.9 (54.6-56.9)[g,k]	18.6 (18.5-18.7)[k,l]	134.2 (131.6-136.6)	326.5 (320.8-331.6)[g,k]
RE	173.3 (170.7-176.1)	26 (25.8-26.2)	199.3 (196.6-202.2)	34.6 (34.3-34.7)	134.3 (132.7-135.8)	40.1 (39.9-40.3)	112.6 (111.1-114.2)	203.1 (199.5-206.6)	48.7 (48.4-49)	293.2 (288.7-297.4)	866.3 (854.9-878)
SH	96.5 (94.8-97.9)	18.8 (18.7-18.9)	115.3 (113.5-116.9)	19.9 (19.8-20)	26.2 (26.1-26.3)	42.1 (41.8-42.4)	85 (83.5-86.6)	77.2 (75.6-78.5)	27.9 (27.8-28)	169 (166.5-171.8)	447.6 (441.4-453.9)
AM	113.1 (111.2-115.2)	15.7 (15.6-15.8)	128.8 (126.8-131.1)	21.9 (21.8-22)	14.7 (14.6-14.8)	15.7 (15.6-15.8)[p]	82.9 (81.4-84.4)	144.5 (142.3-146.9)	68.5 (67.2-70.2)	142.6 (140.4-144.7)	491.3 (483.6-499.1)

[†] Hydroxybenzoic acids were quantified as gallic acid equivalents, and flavanols were quantified as catechin equivalents. The Kruskal-Wallis test, a non-parametric alternative, was applied. Data were presented as medians with interquartile ranges (25th–75th percentiles). The significance of the values was adjusted using Bonferroni correction for multiple tests. A significance level of $p < 0.05$ was considered statistically significant.

Where: [a] represents statistically significant compared with BL; [g] represents statistically significant compared with NE; [h] represents statistically significant compared with RU; [i] represents statistically significant compared with SB; [j] represents statistically significant compared with FR; [k] represents statistically significant compared with RA; [l] represents statistically significant compared with BR; [o] represents statistically significant compared with RE; [p] represents statistically significant compared with SH.

The white grape cultivars are Blasius (BL), Rhine Riesling (RR), Roze Blaj (RB), Astra (AS), Traminer roz (TR), Johaniter (JO), Neuburger (NE), Rubin (RU), Sauvignon Blanc (SB), Fetească Regală (FR), Radames (RA), Brumăriu (BR), Selena (SE) Muscat Ottonel (MO) and the red grape cultivars included Regent (RE), Shiraz (SH), and Amurg (AM).

The analyzed solutions were obtained from 0.1 g of grape pomace (GP) powder which were extracted in a 3 mL solvent mixture of water : ethanol (30:70, v/v).

Table S3. Flavonols (μg/mL rutin equivalents) and anthocyanins (μg/mL cyanidin equivalents) determined in the grape pomace extracts by LC-MS

Compounds concentration (μg/mL) / Samples	RT (min); $[M+H]^+$ (*m/z*); λ_{max} (nm)							
	Quercetin-glucoside 16.23 (min); 465 (*m/z*); 263, 355 (nm)	Kaempferol-glucoside 17.33 (min); 449 (*m/z*); 253, 350 (nm)	Total Flavonols	Malvidin-caffeoyl-glucoside 11.00 (min); 655 (*m/z*); 278, 523 (nm)	Malvidin-glucoside 12.57 (min); 493 (*m/z*); 278, 520 (nm)	Malvidin-acetyl-glucoside 14.70 (min); 535 (*m/z*); 278, 521 (nm)	Malvidin-coumaroyl-glucoside 17.47 (min); 639 (*m/z*); 278, 522 (nm)	Total Anthocyanins
BL	4.21 (4.2-4.2)	0.03 (0.03-0.03)[d,q]	4.2 (4.2-4.3)	n.d.	n.d.	n.d.	n.d.	n.d.
RR	3.1 (3-3.1)	0.09 (0.09-0.09)[d]	3.2 (3.1-3.2)	n.d.	n.d.	n.d.	n.d.	n.d.
RB	2.6 (2.6-2.7)	1.03 (1.02-1.03)	3.7 (3.6-3.7)	n.d.	n.d.	n.d.	n.d.	n.d.
AS	12.4 (12.2-12.5)	5.9 (5.5-6.1)	18.4 (17.8-18.6)	n.d.	n.d.	n.d.	n.d.	n.d.
TR	5.6 (5.5-5.7)	0.63 (0.63-0.63)	6.2 (6.1-6.3)	n.d.	n.d.	n.d.	n.d.	n.d.
JO	11.5 (11.3-11.7)	4.3 (4.3-4.3)	15.9 (15.7-16.1)	n.d.	n.d.	n.d.	n.d.	n.d.
NE	10 (10-10)	2.2 (2.2-2.2)	12.3 (12.3-12.3)	n.d.	n.d.	n.d.	n.d.	n.d.
RU	4.2 (4.1-4.3)	0.91 (0.90-0.92)	5.1 (5-5.3)	n.d.	n.d.	n.d.	n.d.	n.d.
SB	3.6 (3.6-3.8)	0.27 (0.26-0.27)	3.9 (3.7-4)	n.d.	n.d.	n.d.	n.d.	n.d.
FR	4.3 (4.2-4.5)	0.79 (0.79-0.80)	5.1 (5-5.3)	n.d.	n.d.	n.d.	n.d.	n.d.
RA	1.7 (1.7-1.7)[o]	0.42 (0.41-0.43)	2.2 (2.1-2.2)[o]	n.d.	n.d.	n.d.	n.d.	n.d.
BR	8 (7.9-8.1)	2.4 (2.4-2.4)	10.4 (10.3-10.5)	n.d.	n.d.	n.d.	n.d.	n.d.
SE	1.7 (1.7-1.7)[d,g]	0.35 (0.25-0.35)	2 (2-2)[d,o]	n.d.	n.d.	n.d.	3.2 (3.12-3.37)	3.2 (3.12-3.37)

MO	5.7 (5.6-5.8)	0.25 (0.25-0.25)	5.9 (5.8-6.1)	n.d.	n.d.	n.d.	n.d.	n.d.
RE	16.5 (16.3-16.7)	4.5 (4.4-4.7)	21.1 (20.8-21.4)	6.2 (6.13-6.42)	6.1 (5.93-6.17)	5.0 (4.92-5.14)	10.1 (9.90-10.20)	27.5 (26.89-27.95)
SH	5.8 (5.7-5.9)	2.2 (2.1-2.3)	8.1 (7.8-8.30	n.d.	3.4 (3.25-3.55)	3.1 (3.04-3.29)	3.0 (2.89-3.11)	9.5 (9.19-9.96)
AM	8.1 (8-8.2)	5.7 (5.5-5-8)	13.9 (13.6-14.1)	n.d.	4.9 (4.79-5.06)	3.6 (3.50-3.70)	4.5 (4.35-4.64)	13.0 (12.66-13.42)

[ǂ] Flavonols were quantified as rutin equivalents and anthocyanins as cyanidin equivalents (μg/mL). The Kruskal-Wallis test, a non-parametric alternative, was applied. Data were presented as medians with interquartile ranges (25th–75th percentiles). The significance of the values was adjusted using Bonferroni correction for multiple tests. A significance level of $p < 0.05$ was considered statistically significant.

Where: [d] represents statistically significant compared with AS; [g] represents statistically significant compared with NE; [o] represents statistically significant compared with RE; [q] represents statistically significant compared with AM; n.d. – non determined

The white grape cultivars are Blasius (BL), Rhine Riesling (RR), Roze Blaj (RB), Astra (AS), Traminer roz (TR), Johaniter (JO), Neuburger (NE), Rubin (RU), Sauvignon Blanc (SB), Fetească Regală (FR), Radames (RA), Brumăriu (BR), Selena (SE) Muscat Ottonel (MO) and the red grape cultivars included Regent (RE), Shiraz (SH), and Amurg (AM).

The analyzed solutions were obtained from 0.1 g of grape pomace (GP) powder which were extracted in a 3 mL solvent mixture of water : ethanol (30:70, v/v).

Table S4. Fatty acids composition (g/100 g of dried sample) of the studied grape pomaces by GC-FID

Sample/ Fatty acid (g FAME/ 100 g Total FAME)	BL	RR	AM	RB	AS	SH	TR	BR	JO	RE	SE	NE	MO	RU	RA	SB	FR
								Saturated Fatty Acids (SFA)									
Caprilic (C 8:0)	0.06 (0.05-0.08)	0.50 (0.46-0.54)	0.06 (0.04-0.07)	0.58 (0.55-0.620	0.01 (0.01-0.01)	0.14 (0.14-0.14)	0.04 (0.04-0.04)	0.00 (0.00-0.00)	0.02 90.01-0.03)	0.92 (0.89-0.95)	0.05 (0.04-0.05)	0.06 (0.05-0.07)	0.02 (0.01-0.03)	0.05 (0.03-0.06)	0.05 (0.04-0.06)	0.00 (0.00-0.00)	0.06 (0.04-0.07)
Capric (C 10:0)	0.12 (0.11-0.13)	0.09 (0.09-0.10)	0.11 (0.10-0.12)	0.04 (0.04-0.05)	0.04 (0.03-0.05)	0.15 (0.14-0.15)	0.07 (0.06-0.07)	0.07 (0.07-0.08)	0.14 (0.13-0.15)	0.09 (0.08-0.10)	0.06 (0.05-0.06)	0.07 (0.06-0.07)	0.04 (0.04-0.05)	0.10 (0.10-0.10)	0.13 (0.13-0.13)	0.08 (0.08-0.08)	0.07 (0.06-0.08)
Lauric (C12:0)	0.04 (0.04-0.05)	0.04 (0.04-0.05)	0.02 (0.01-0.02)	0.01 (0.01-0.01)	0.02 (0.01-0.02)	0.03 (0.03-0.04)	0.02 (0.02-0.02)	0.06 (0.06-0.07)	0.06 (0.05-0.07)	0.04 (0.04-0.04)	0.04 (0.03-0.05)	0.06 (0.05-0.08)	0.02 (0.02-0.03)	0.03 (0.02-0.03)	0.01 (0.00-0.02)	0.03 (0.02-0.04)	0.03 (0.02-0.03)
Miristic (C 14:0)	0.45 (0.43-0.46)	0.30 (0.29-0.31)	0.28 (0.27-0.29)	0.15 (0.14-0.17)	0.13 (0.13-0.13)	0.36 (0.35-0.36)	0.24 (0.22-0.25)	0.24 (0.24-0.25)	0.59 (0.53-0.65)	0.22 (0.21-0.23)	0.22 (0.21-0.24)	0.34 (0.33-0.36)	0.18 (0.18-0.19)	0.24 (0.23-0.24)	0.27 (0.27-0.27)	0.26 (0.26-0.26)	0.26 (0.25-0.28)
Pentadecanoic (C15:0)	0.13 (0.13-0.14)	0.10 (0.10-0.10)	0.09 (0.09-0.09)	0.03 (0.02-0.03)	0.02 (0.02-0.03)	0.15 (0.14-0.16)	0.08 (0.07-0.08)	0.03 (0.03-0.04)	0.06 (0.05-0.08)	0.05 (0.05-0.06)	0.04 (0.04-0.04)	0.18 (0.16-0.20)	0.06 (0.06-0.07)	0.10 (0.09-0.12)	0.10 (0.09-0.11)	0.04 (0.04-0.05)	0.04 (0.03-0.06)
Palmitic (C16:0)	15.53 (15.49-15.58)	12.86 (12.85-12.87)	12.43 (12.33-12.53)	9.07 (9.04-9.10)	8.92 (8.89-8.95)	11.55 (11.51-11.59)	12.02 (11.98-12.06)	11.32 (11.31-11.33)	12.22 (12.19-12.26)	11.08 (11.05-11.12)	10.40 (10.36-10.44)	13.82 (13.78-13.87)	11.68 (11.67-11.68)	10.83 (10.82-10.84)	11.99 (11.96-12.03)	11.76 (11.74-11.78)	12.28 (12.27-12.30)
Heptadecanoic (C17:0)	0.01 (0.00-0.02)	0.10 (0.10-0.10)	0.03 (0.03-0.04)	0.02 (0.02-0.02)	0.02 (0.02-0.02)	0.02 (0.02-0.03)	0.10 (0.09-0.11)	0.09 (0.09-0.10)	0.10 (0.09-0.11)	0.03 (0.03-0.04)	0.04 (0.00-0.08)	0.07 (0.06-0.09)	0.11 (0.10-0.12)	0.08 (0.07-0.10)	0.08 (0.07-0.09)	0.10 (0.09-0.10)	0.07 90.05-0.09)
Stearic (C18:0)	4.45 (4.33-4.57)	4.12 (4.09-4.14)	3.67 (3.64-3.69)	3.47 (3.44-347)	4.68 (4.67-4.69)	4.08 (4.06-4.10)	5.03 (5.02-5.04)	3.20 (3.19-3.20)	4.21 (4.20-4.22)	3.96 (3.94-3.97)	3.59 (3.52-3.65)	4.83 (4.79-4.87)	3.93 (3.91-3.96)	5.24 (5.19-5.30)	4.33 (4.31-4.35)	4.76 (4.72-4.80)	4.10 (4.09-4.11)
Arahidic (C 20:0)	0.00 (0.00-0.00)	0.00 (0.00-0.00)	0.01 (0.00-0.01)	0.05 (0.05-0.05)	0.01 (0.01-0.01)	0.00 (0.00-0.00)	0.04 (0.04-0.05)	0.03 (0.03-0.04)	0.07 (0.07-0.08)	0.06 (0.06-0.07)	0.03 (0.02-0.03)	0.03 (0.03-0.03)	0.02 (0.02-0.03)	0.03 (0.03-0.04)	0.05 (0.04-0.06)	0.03 (0.03-0.04)	0.43 (0.41-0.44)
Lignoceric (C 24:0)	0.23 (0.22-0.24)	0.19 (0.19-0.20)	0.14 (0.13-0.14)	0.13 (0.12-0.14)	0.16 (0.15-0.17)	0.27 (0.27-0.28)	0.24 (0.24-0.25)	0.16 (0.15-0.17)	0.15 (0.14-0.16)	0.21 (0.21-0.22)	0.18 (0.15-0.20)	0.21 (0.20-0.22)	0.18 (0.18-0.19)	0.22 (0.21-0.23)	0.19 (0.19-0.20)	0.24 (0.22-0.26)	0.22 (0.21-0.22)

Total SFA	21.03 (20.69-21.11)	17.85 (17.85-17.86)	16.84 (16.77-16.91)	13.55 (13.47-13.63)	14.01 (13.99-14.03)	16.76 (16.69-16.83)	17.87 (17.82-17.93)	15.22 (15.18-15.26)	17.63 (17.56-17.71)	16.68 (16.68-16.68)	14.64 (14.51-14.76)	19.69 (19.67-19.71)	16.26 (16.18-16.34)	16.93 (16.87-16.99)	17.21 (17.13-17.28)	17.30 (17.28-17.32)	18.16 (18.14-18.18)
Unsaturated Fatty Acids (UFA)																	
Monounsaturated Fatty Acids (MUFA)																	
Pentadecenoic (C15:1)	0.05 (0.04-0.05)	0.02 (0.02-0.03)	0.02 (0.02-0.03)	0.02 (0.02-0.02)	0.01 (0.01-0.02)	0.05 (0.05-0.05)	0.03 (0.02-0.03)	0.05 (0.04-0.06)	0.04 (0.04-0.04)	0.03 (0.03-0.03)	0.04 (0.02-0.05)	0.12 (0.12-0.12)	0.02 (0.02-0.03)	0.10 (0.09-0.12)	0.13 (0.11-0.15)	0.05 (0.05-0.06)	0.04 (0.04-0.04)
Palmitoleic (C16:1)	1.81 (1.80-1.83)	1.17 (1.16-1.18)	1.14 (1.12-1.15)	0.46 (0.44-0.49)	0.73 (0.72-0.74)	1.05 (1.04-1.06)	0.97 (0.96-0.99)	1.54 (1.53-1.54)	1.36 (1.35-1.36)	0.78 (0.77-0.78)	0.56 (0.54-0.59)	1.23 (1.22-1.25)	0.73 (0.72-0.74)	0.87 (0.86-0.87)	1.36 (1.31-1.40)	0.75 (0.73-0.78)	0.95 (0.92-0.97)
Oleic cis (C18:1)	17.71 (17.71-17.72)	17.01 (16.97-17.04)	18.39 (18.34-18.44)	16.48 (16.46-16.49)	15.82 (15.79-15.86)	13.63 (13.62-13.64)	16.53 (16.50-16.56)	18.72 (18.66-18.78)	14.78 (14.71-14.86)	14.22 (14.20-14.24)	16.48 (16.41-16.54)	17.86 (17.85-17.87)	17.16 (17.13-17.19)	15.81 (15.80-15.83)	16.28 (16.21-16.35)	15.68 (15.63-15.73)	16.15 (16.08-16.21)
Total MUFA	19.57 (19.56-19.59)	18.20 (18.17-18.23)	19.55 (19.49-19.61)	16.96 (16.95-16.97)	16.57 (16.52-16.61)	14.73 (14.72-14.74)	17.53 (17.52-17.54)	20.30 (20.23-20.38)	16.18 (16.10-16.26)	15.02 (15.00-15.05)	17.08 (17.00-17.15)	19.21 (19.18-19.23)	17.91 (17.87-17.95)	16.77 (16.77-16.78)	17.76 (17.76-17.77)	16.49 (16.41-16.56)	17.13 (17.09-17.18)
Polyunsaturated Fatty Acids (PUFA)																	
Polyunsaturated Fatty Acids omega 3 (PUFA ω-3)																	
a-Linoleic (C18:3n3)	1.60 (1.60-1.610	1.30 (1.30-1.31)	1.87 (1.87-1.87)	0.70 (0.69-0.70)	0.64 (0.63-0.650	1.11 (1.11-1.11)	1.17 (1.16-1.170	1.49 (1.49-1.50)	1.74 (1.74-1.75)	1.85 (1.84-1.86)	1.01 (1.01-1.01)	1.50 (1.50-1.50)	1.06 (1.06-1.07)	1.15 (1.14-1.17)	1.15 (1.14-1.15)	1.30 (1.30-1.310	1.11 (1.10-1.11)
Octadecatetraenoic (C18:4n3)	0.51 (0.51-0.52)	0.44 (0.42-0.46)	0.28 (0.28-0.29)	0.17 (0.15-0.19)	0.40 (0.39-0.42)	0.33 (0.31-0.35)	0.62 (0.62-0.63)	0.50 (0.50-0.51)	0.50 (0.48-0.52)	0.26 (0.25-0.28)	0.43 (0.39-0.46)	1.12 (1.10-1.14)	0.52 (0.50-0.55)	1.27 (1.23-1.31)	1.28 (1.26-1.30)	0.55 (0.52-0.57)	0.28 (0.28-0.28)
Eicosatrienoic (C20(3n3))	0.04 (0.03-0.04)	0.05 (0.05-0.05)	0.11 (0.11-0.12)	0.07 (0.07-0.08)	0.11 (0.11-0.12)	0.16 (0.14-0.19)	0.11 (0.11-0.11)	0.06 (0.06-0.06)	0.07 (0.07-0.08)	0.00 (0.00-0.00)	0.05 (0.04-0.06)	0.07 (0.06-0.09)	0.06 (0.06-0.07)	0.07 (0.07-0.08)	0.08 (0.08-0.09)	0.07 (0.07-0.08)	0.06 (0.06-0.06)
Eicosapentaenoic (C 20:5n3)	0.18 (0.17-0.18)	0.14 (0.13-0.14)	0.12 (0.12-0.13)	0.06 (0.05-0.07)	0.08 (0.08-0.08)	0.10 (0.10-0.10)	0.18 (0.18-0.19)	0.13 (0.12-0.13)	0.14 (0.13-0.15)	0.11 (0.11-0.11)	0.14 (0.12-0.16)	0.20 (0.20-0.20)	0.14 (0.14-0.14)	0.20 (0.19-0.20)	0.18 (0.16-0.19)	0.16 (0.14-0.19)	0.16 (0.14-0.18)
Total PUFA ω-3	2.33 (2.32-2.34)	1.93 (1.91-1.95)	2.39 (2.39-2.39)	1.00 (0.99-1.01)	1.24 (1.23-1.25)	1.70 (1.69-1.71)	2.08 (2.07-2.10)	2.18 (2.17-2.18)	2.45 (2.44-2.47)	2.23 (2.23-2.23)	1.62 (1.61-1.63)	2.90 (2.89-2.90)	1.78 (1.76-1.81)	2.69 (2.66-2.72)	2.69 (2.68-2.69)	2.08 (2.02-2.14)	1.60 (1.60-1.61)
Polyunsaturated Fatty Acids omega 6 (PUFA ω-6)																	

Linoleic cis (C 18:2n6)	55.68 (55.60-55.76)	61.04 (61.00-61.07)	60.47 (60.43-60.52)	67.86 (67.83-67.90)	67.29 (67.28-67.31)	66.13 (66.12-66.14)	60.70 (60.69-60.71)	60.80 (60.66-60.93)	61.58 (61.48-61.68)	65.61 (65.57-65.65)	65.49 (65.38-65.59)	56.95 (56.92-56.99)	62.60 (62.58-62.62)	61.63 (61.60-61.65)	60.56 (60.51-60.62)	62.76 (62.74-62.77)	62.12 (62.10-62.15)
Eicosadienoic (C20(2n6))	0.30 (0.29-0.31)	0.13 (0.12-0.14)	0.13 (0.12-0.13)	0.17 (0.16-0.18)	0.19 (0.18-0.20)	0.07 (0.07-0.07)	0.15 (0.14-0.15)	0.52 (0.50-0.55)	0.22 (0.22-0.23)	0.11 (0.11-0.12)	0.30 (0.28-0.33)	0.24 (0.20-0.28)	0.11 (0.10-0.11)	0.57 (0.52-0.630	0.61 (0.61-0.620	0.24 (0.22-0.27)	0.07 (0.07-0.08)
Eicosatrienoic (C20(3n6))	0.04 (0.04-0.05)	0.16 (0.16-0.16)	0.19 (0.19-0.19)	0.06 (0.05-0.06)	0.08 (0.07-0.09)	0.07 (0.06-0.07)	0.11 (0.11-0.11)	0.07 (0.06-0.08)	0.11 (0.10-0.11)	0.00 (0.00-0.00)	0.04 (0.04-0.05)	0.17 (0.16-0.18)	0.06 (0.05-0.06)	0.13 (0.10-0.16)	0.14 (0.11-0.18)	0.10 (0.09-0.11)	0.14 (0.13-0.14)
Arachidonic (C20(4n6))	0.26 (0.25-0.27)	0.04 (0.03-0.05)	0.04 (0.04-0.04)	0.07 (0.06-0.07)	0.05 (0.05-0.05)	0.04 (0.04-0.05)	0.39 (0.37-0.40)	0.11 (0.10-0.11)	0.27 (0.26-0.29)	0.08 (0.08-0.08)	0.10 (0.09-0.11)	0.27 (0.24-0.31)	0.18 (0.17-0.18)	0.22 (0.18-0.27)	0.20 (0.17-0.23)	0.11 (0.11-0.12)	0.10 (0.10-0.11)
Docosadienoic (C 22:2n6)	0.06 (0.06-0.07)	0.06 (0.06-0.07)	0.06 (0.05-0.06)	0.03 (0.03-0.04)	0.06 (0.05-0.06)	0.09 (0.08-0.09)	0.55 (0.53-0.58)	0.55 (0.55-0.55)	0.64 (0.63-0.66)	0.13 (0.13-0.13)	0.57 (0.56-0.58)	0.35 (0.34-0.36)	0.40 (0.40-0.40)	0.74 (0.66-0.81)	0.59 (0.53-0.650	0.65 (0.61-0.68)	0.06 (0.05-0.07)
Total PUFA ω-6	56.35 (56.24-56.45)	61.42 (61.38-61.46)	60.89 (60.83-60.940	68.19 (68.12-68.25)	67.67 (67.64-67.70)	66.40 (66.38-66.41)	61.89 (61.88-61.90)	62.04 (61.93-62.16)	62.83 (62.74-62.92)	65.93 (65.90-65.97)	66.50 (66.39-66.61)	57.98 (57.97-58.00)	63.34 (63.33-63.35)	63.29 (63.26-63.32)	62.10 (61.99-62.20)	63.86 (63.86-63.86)	62.50 (62.47-62.53)
Total PUFA	58.67 (58.58-58.77)	63.35 (63.33-63.37)	63.28 (63.22-63.33)	69.19 (69.14-69.24)	68.91 (68.89-69.93)	68.09 (68.09-68.10)	63.98 (63.95-64.00)	64.22 (64.11-64.33)	65.28 (65.18-65.39)	68.16 (68.12-68.19)	68.12 (68.00-68.24)	60.88 (60.88-60.89)	65.12 (65.09-65.150	65.98 (65.92-66.04)	64.78 (64.68-64.89)	65.94 (65.88-66.00)	64.10 (64.07-64.14)
Total UFA (MUFA+PUFA)	78.25 (78.14-78.36)	81.55 (81.50-81.60)	82.83 (82.71-82.95)	86.15 (86.11-86.19)	85.47 (85.41-85.54)	82.82 (82.80-82.85)	81.51 (81.47-81.54)	84.52 (84.49-84.56)	81.46 (81.44-81.49)	83.18 (83.17-83.19)	85.20 (85.15-85.24)	80.09 (80.06-80.12)	83.04 (82.97-83.11)	82.75 (82.69-82.82)	82.55 (82.45-82.64)	82.43 (82.41-82.45)	81.23 (81.22-81.25)
Other fatty acids	0.72 (0.69-0.76)	0.60 (0.55-0.650	0.34 (0.29-0.390	0.30 (0.26-0.34)	0.52 (0.47-0.57)	0.42 (0.37-0.46)	0.62 (0.60-0.64)	0.26 (0.26-0.26)	0.91 (0.86-0.96)	0.14 (0.13-0.15)	0.17 (0.09-0.25)	0.22 (0.21-0.24)	0.70 (0.70-0.71)	0.32 (0.31-0.33)	0.24 (0.23-0.26)	0.27 (0.27-0.27)	0.61 (0.60-0.62)
SFA/UFA	0.27 (0.27-0.27)	0.22 (0.22-0.22)	0.20 (0.20-0.20)	0.16 (0.16-0.16)	0.16 (0.16-0.16)	0.20 (0.20-0.20)	0.22 (0.22-0.22)	0.18 (0.18-0.18)	0.22 (0.22-0.22)	0.20 (0.20-0.20)	0.17 (0.17-0.17)	0.25 (0.25-0.25)	0.20 (0.20-0.20)	0.20 (0.20-0.20)	0.21 (0.21-0.21)	0.21 (0.21-0.21)	0.22 (0.22-0.22)
PUFA/MUFA	3.00 (3.00-3.00)	3.48 (3.48-3.48)	3.24 (3.23-3.24)	4.08 (4.07-4.09)	4.16 (4.15-4.17)	4.62 (4.62-4.63)	3.65 (3.65-3.650	3.16 (3.15-3.18)	4.04 (4.01-4.06)	4.54 (4.53-4.55)	3.99 (3.96-4.01)	3.17 (3.17-3.17)	3.64 (3.63-3.64)	3.93 (3.93-3.94)	3.65 (3.64-3.65)	4.00 (3.98-4.02)	3.74 (3.73-3.75)
ω-6 / ω-3	24.19 (24.05-24.33)	31.87 (31.48-32.25)	25.50 (25.50-25.51)	68.11 (67.17-69.06)	54.69 (54.29-55.10)	39.10 (38.94-39.26)	29.69 (29.50-29.88)	28.52 (28.35-28.69)	25.61 (25.48-25.74)	29.63 (29.62-29.65)	41.06 (40.82-41.30)	20.01 (19.96-20.06)	35.55 (35.11-36.00)	23.51 (23.28-23.75)	23.12 (23.04-23.20)	30.71 (29.86-31.56)	38.95 (38.83-39.07)

UFA/SFA	3.72 (3.70-3.74)	4.57 (4.57-4.57)	4.92 (4.89-4.95)	6.36 (6.32-6.40)	6.10 (6.09-6.120	4.94 (4.92-4.96)	4.56 (4.55-4.58)	5.55 (5.54-5.57)	4.62 (4.60-4.62)	4.99 (4.99-4.99)	5.82 (5.77-5.87)	4.07 (4.06-4.07)	5.11 (5.08-5.14)	4.89 (4.87-4.91)	4.80 (4.77-4.82)	4.76 (4.76-4.77)	4.47 (4.47-4.48)
PUFA/SFA	2.79 (2.78-2.81)	3.55 (3.55-3.55)	3.76 (3.74-3.78)	5.11 (5.07-5.14)	4.92 (4.91-4.93)	4.06 (4.05-4.08)	3.58 (3.57-3.59)	4.22 (4.20-4.24)	3.70 (3.68-3.73)	4.09 (4.08-4.09)	4.65 (4.61-4.70)	3.09 (3.09-3.10)	4.01 (3.98-4.03)	3.90 (3.88-3.920	3.77 (3.74-3.79)	3.81 (3.81-3.81)	3.53 (3.53-3.53)
COX	6.26 (6.25-6.27)	6.74 (6.74-6.74)	6.82 (6.81-6.82)	7.31 (7.30-7.31)	7.23 (7.23-7.23)	7.19 (7.19-7.19)	7.67 (7.67-7.67)	6.77 (6.76-6.78)	6.87 (6.86-6.88)	7.30 (7.30-7.30)	7.13 (7.12-7.14)	6.37 (6.37-6.37)	6.85 (6.85-6.85)	6.76 (6.75-6.76)	6.65 (6.64-6.65)	6.90 (6.90-6.90)	6.80 (6.80-6.80)
AI	0.22 (0.22-0.22)	0.17 (0.17-0.17)	0.16 (0.16-0.17)	0.11 (0.11-0.11)	0.11 (0.11-0.11)	0.16 (0.16-0.16)	0.16 (0.16-0.16)	0.15 (0.15-0.15)	0.18 (0.18-0.18)	0.14 (0.14-0.14)	0.13 (0.13-0.13)	0.19 (0.19-0.19)	0.15 (0.15-0.15)	0.14 (0.14-0.14)	0.16 (0.16-0.16)	0.16 (0.16-0.16)	0.16 (0.16-0.17)
TI	0.45 (0.45-0.46)	0.38 (0.38-038)	0.35 (0.34-0.35)	0.28 (0.28-0.28)	0.30 (0.30-0.30)	0.35 (0.35-0.35)	0.38 (0.37-0.380	0.31 (0.31-0.31)	0.36 (0.36-0.36)	0.32 (0.32-0.32)	0.30 (0.30-0.31)	0.40 (0.40-0.40)	0.34 (0.34-0.35)	0.34 (0.34-0.34)	0.35 (0.34-0.35)	0.36 (0.36-0.36)	0.37 (0.37-0.37)
H/H	4.69 (4.69-4.70)	6.03 (6.02-6.04)	6.35 (6.30-6.41)	9.22 (9.17-9.27)	9.25 (9.22-9.29)	6.79 (6.76-6.82)	6.40 (6.37-6.43)	7.00 (7.00-7.01)	6.10 (6.08-6.11)	7.23 (7.21-7.24)	7.82 (7.77-7.86)	5.39 (5.38-5.40)	6.81 (6.80-6.83)	7.10 (7.10-7.110	6.36 (6.34-6.38)	6.63 (6.63-6.64)	6.33 (6.33-6.33)
%	100	100	100	100	100	100	100	100	100	100	100	100	100	100	100	100	100

The Kruskal-Wallis test, a non-parametric alternative, was applied. Fatty acid methyl esters (FAME) (g/100 g of dried sample) data were presented as medians with interquartile ranges (25th–75th percentiles). The significance of the values was adjusted using Bonferroni correction for multiple tests. A significance level of $p < 0.05$ was considered statistically significant.

The white grape cultivars are Blasius (BL), Rhine Riesling (RR), Roze Blaj (RB), Astra (AS), Traminer roz (TR), Johaniter (JO), Neuburger (NE), Rubin (RU), Sauvignon Blanc (SB), Fetească Regală (FR), Radames (RA), Brumăriu (BR), Selena (SE) Muscat Ottonel (MO) and the red grape cultivars included Regent (RE), Shiraz (SH), and Amurg (AM).

FAME-fatty acids methyl esters, SFA-saturated fatty acids. MUFA-monounsaturated fatty acids, PUFA-polyunsaturated fatty acids,

Total SFA (total saturated fatty acids) = C14:0 + C16:0 + C17:0+C18:0; Total MUFA (total monounsaturated fatty acids) = C14:1; C16:1 + C17:1; C18:1n-9; C18:1n-11; Total PUFA (total polyunsaturated fatty acids) = C18:2n-6 + C18:3n-3 + C18:4 n-3; C20:2n-6 + CLA+ C20:4n-6 + C20:5n-3 + C22-5 n-3 + C22:6n-3; Total n-6 PUFA = C18:2n-6 + C20:2n-6, + CLA+ C20:4n-6; total n-3 PUFA = C18:3n-3 + C18:4 n-3 + C20:5n-3 + C22-5 n-3 + C22:6n-3.)

Table S5. Antiradical activity of the samples evaluated using the capacity of scavenging $DPPH^{\cdot}$ and $ABTS^{\cdot+}$, the electron donation capacity using the FRAP, RP and CUPRAC methods and the ferrous and cupric ions chelation capacity

Sample/ Antioxidant activity	DPPH	ABTS	CUPRAC	FRAP	RP	Ferrous ions chelation	Cupric ions chelation
	µM TE / mL extract	mg GAE / mL extract	mg GAE / mL extract	mg GAE / mL extract	mg GAE/ mL extract	µM EE / mL extract	µM EE / mL extract
BL	36.93 (36.40-37.18) g	1.67 (1.65-1.69)	0.42 (0.41-0.43)	1.17 (1.13-1.17)	0.42 (0.41-0.43)	0.22 (0.21-0.22)	0.41 (0.40-0.42)
RR	46.72 (46.46-46.92)	1.99 (1.96-2)	0.53 (0.52-0.54)	1.16 (1.14-1.18)	0.41 (0.41-0.41)	0.24 (0.24-0.24)	0.33 (0.33-0.33)
RB	45.69 (45.6-46.02)	1.87 (1.84-1.89)	0.51 (0.50-051)	1.21 (1.19-1.22)	0.48 (0.47-0.48)	0.26 (0.25-0.27)	0.45 (0.44-0.45)
AS	36.23 (36.08-36.33) g, j	1.49 (1.47-1.5) g, j	0.38 (0.38-0.38) e, g	0.96 (0.95-0.99) g	0.37 (0.36-0.38) g	0.09 (0.09-0.09)	0.28 (0.26-0.28)
TR	77 (76.85-77.17)	2.97 (2.95-3.02)	1.08 (1.07-1.08)	1.64 (1.63-1.68)	1.01 (1.00-1.02)	0.24 (0.24-0.24)	0.37 (0.37-0.39)
JO	80.24 (80.17-80.44)	2.98 (2.95-3)	1.05 (1.03-1.05)	1.79 (1.77-1.81)	1.13 (1.10-1.14)	0.25 (0.24-0.25)	0.41 (0.40-0.42)
NE	115.45 (114.9-115.88)	3.87 (3.85-3.91)	1.28 (1.28-1.29)	2.02 (2.00-2.07)	1.47 (1.44-1.48)	0.26 (0.26-0.27)	0.46 (0.44-0.47)
RU	43.98 (43.43-44.43)	1.54 (1.51-1.58) g	038 (0.37-0.39) e,g	0.91 (0.89-0.91) g,j	0.35 (0.32-0.37) g,j	0.08 (0.07-0.08) g	0.25 (0.23-0.25) c,g
SB	56.88 (56.59-57.17)	2.01 (1.99-2.03)	0.59 (0.58-0.60)	1.17 (1.15-1.18)	0.77 (0.76-0.78)	0.19 (0.17-0.19)	0.41 (0.39-0.42)
FR	90/76 (90.21-90.98)	3.09 (3.07-3.13)	0.99 (0.97-1.00)	1.86 (1.81-1.87)	1.26 (1.25-1.28)	0.26 (0.24-0.26)	0.39 (0.39-0.41)
RA	79.67 (79.61-79.89)	2.94 (2.93-2.97)	0.80 (0.78-0.80)	1.43 (1.41-1.43)	0.87 (0.86-0.88)	0.25 (0.24-0.26)	0.35 (0.34-0.36)
BR	51.12 (51.04-51.16)	2.32 (2.31-2.33)	0.52 (0.51-0.53)	1.09 (1.08-1.09)	0.49 (0.48-0.50)	0.22 (0.21-0.23)	0.34 (0.34-0.34)
SE	41.71 (41.51-41.91)	1.62 (1.61-1.64)	0.51 (0.50-0.51)	1.16 (1.14-1.19)	0.58 (0.57-0.60)	0.21 (0.21-0.21)	0.32 (0.31-0.32)
MO	60.42 (59.99-60.93)	2.1 (2.8-2.12)	0.55 (0.55-0.55)	1.12 (1.10-1.12)	0.73 (0.72-0.75)	0.22 (0.21-0.23)	0.36 (0.35-0.37)
RE	59.96 (59.65-60.22)	2.78 (2.78-2.79)	0.73 (0.72-0.75)	1.55 (1.52-1.56)	0.83 (0.82-0.85)	0.19 (0.19-0.20)	0.41 (0.40-0.42)

SH	50.82 (50.44-51.19)	2.31 (2.3-2.32)	0.53 (0.53-0.54)	1.19 (1.17-1.22)	0.61 (0.58-0.61)	0.17 (0.16-0.17)	0.31 (0.29-0.31)
AM	37.35 (37.04-37.58)	1.51 (1.50-1.53) [g]	0.46 (0.46-0.46)	1.14 (1.11-1.56)	0.51 (0.50-0.51)	0.18 (0.17-0.18)	0.35 (0.34-0.36)

[†] Values represent medians with interquartile ranges (25th–75th percentiles). The Kruskal-Wallis test, a non-parametric alternative, was applied. The significance of the values was adjusted using Bonferroni correction for multiple tests. A significance level of $p < 0.05$ was considered statistically significant.

Where: [c] represents statistically significant compared with RB; [e] represents statistically significant compared with TR; [g] represents statistically significant compared with NE; [j] represents statistically significant compared with FR.

The white grape cultivars are Blasius (BL), Rhine Riesling (RR), Roze Blaj (RB), Astra (AS), Traminer roz (TR), Johaniter (JO), Neuburger (NE), Rubin (RU), Sauvignon Blanc (SB), Fetească Regală (FR), Radames (RA), Brumăriu (BR), Selena (SE) Muscat Ottonel (MO) and the red grape cultivars included Regent (RE), Shiraz (SH), and Amurg (AM).

TE—Trolox equivalent, GAE—gallic acid equivalent, EE—EDTA equivalent; DPPH—2,2-diphenyl-1-picrylhydrazyl assay; ABTS—2,2′-azinobis-(3-ethylbenzthiazolin-6-sulfonic acid) radical-scavenging assay; CUPRAC—Cupric Reducing Antioxidant Capacity; FRAP—Ferric Reducing Antioxidant Potential; RP—Reducing Power

The analyzed solutions were obtained from 0.1 g of grape pomace (GP) powder which were extracted in a 3 mL solvent mixture of water : ethanol (30:70, v/v).
